# Can attraction to and competition for high‐quality habitats shape breeding propensity?

**DOI:** 10.1111/1365-2656.13676

**Published:** 2022-03-11

**Authors:** Paul Acker, Michael Schaub, Aurélien Besnard, Jean‐Yves Monnat, Emmanuelle Cam

**Affiliations:** ^1^ Centre for Biodiversity Dynamics Institutt for Biologi, NTNU Trondheim Norway; ^2^ Laboratoire EDB (UMR 5174) Université Paul Sabatier – CNRS – IRD Toulouse France; ^3^ CEFE, Univ Montpellier, CNRS, EPHE‐PSL University, IRD Montpellier France; ^4^ Swiss Ornithological Institute Sempach Switzerland; ^5^ 6 Pennarun d’An Traon Goulien France; ^6^ Univ Brest, CNRS, IRD, Ifremer, LEMAR Plouzané France

**Keywords:** breeding decision, breeding habitat selection, density dependence, immigration, integrated population model, intraspecific competition, recruitment, reproductive skipping

## Abstract

1. In many animal species, sexually mature individuals may skip breeding opportunities despite a likely negative impact on fitness. In spatio‐temporally heterogeneous environments, habitat selection theory predicts that individuals select habitats where fitness prospects are maximized. Individuals are attracted to high‐quality habitat patches where they compete for high‐quality breeding sites. Since failures in contests to secure a site may prevent individuals from breeding, we hypothesized that attraction to and competition for high‐quality habitats could shape breeding propensity.
2. Under this hypothesis, we predicted the two following associations between breeding propensity and two key population features. (1) When mean habitat quality in the population increases in multiple patches such that availability of high‐quality sites increases across the population, the resulting decrease in competition should positively affect breeding propensity. (2) When the number of individuals increases in the population, the resulting increase in competitors should negatively affect breeding propensity (negative density dependence).
3. Using long‐term data from kittiwakes *Rissa tridactyla*, we checked the prerequisite of prediction (1), that availability of high‐quality sites is positively associated with current mean habitat quality in the population (represented by breeding success). We then applied integrated population modelling to quantify annual fluctuations in population mean breeding success, breeding propensity and number of individuals by breeding status (pre‐breeders, breeders, skippers and immigrants), and tested our predictions.
4. Our results showed that breeding propensity acts as an important driver of population growth. As expected, breeding propensity was positively associated with preceding mean habitat quality in the population, and negatively with the number of competitors. These relationships varied depending on breeding status, which likely reflects status dependence in competitive ability.
5. These findings highlight the importance of competition for high‐quality breeding sites in shaping breeding propensity. Thereby, we draw attention towards alternative and complementary explanations to more standard considerations regarding the energetic cost of reproduction, and point to possible side effects of habitat selection behaviours on individual life histories and population dynamics.

## Graphical abstract

Attraction to and competition for high‐quality breeding sites are critical habitat selection mechanisms. These can be hypothesized to shape breeding propensity, since failure in securing a site may prevent individuals from breeding. Under this hypothesis, the authors predict associations between breeding propensity and key population factors linked to the intensity of competition for high‐quality sites, which they demonstrate in a wild population of kittiwakes. Photo credit: Olivier Normand.

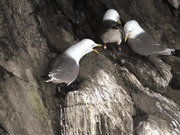

## 1 INTRODUCTION

At each reproductive occasion, sexually mature individuals experience various external and internal constraints that may alter their breeding propensity. This can have major impacts on individual fitness, population growth and demographic structure (Lee et al., 2016; Stearns, 1992). Non‐breeders lose their current reproductive value and are at risk of dying before the next breeding opportunity, yet they often represent a non‐negligible part of the population in long‐lived species. These can be individuals that have not recruited yet (‘pre‐breeders’) or have already bred previously (‘skippers’), as found in a wide range of taxa, spanning fish (e.g. Rideout & Tomkiewicz, 2011), reptiles (e.g. Shine & Brown, 2008), amphibians (e.g. Cayuela et al., 2014), birds (e.g. Bruinzeel, 2007) and mammals (e.g. Desprez et al., 2018). Identifying the factors that lead to non‐breeding is thus critical to understand key eco‐evolutionary processes underlying population dynamics.

Individuals may not breed simply because they do not fulfil essential requirements, that is they did not accumulate sufficient energy reserves (Giudici et al., 2010; Meijer & Drent, 1999) or failed to acquire a mate or a *breeding site* (Box 1; Danchin & Cam, 2002; Bruinzeel, 2007). Meeting these breeding requirements is costly, and subsequent breeding activities are costly too. According to life‐history theory, the proportion of finite resources allocated to current reproduction is traded off against the proportion allocated to survival and/or future reproduction (Stearns, 1992). Consequently, when individuals face high costs of current reproduction, non‐breeding could allow maximizing long‐term fitness prospects and be selectively advantageous (Desprez et al., 2018; Erikstad et al., 1998). This can explain why unfavourable environmental conditions experienced by a population (e.g. lower overall food availability) are associated with lower subsequent *breeding propensity* (Cayuela et al., 2014; Hoy et al., 2016; Rideout & Tomkiewicz, 2011; Shine & Brown, 2008).

Yet environmental conditions are typically varying not only across time, but also across space. In species moving actively, mechanisms of *breeding habitat selection* (Box 1) that allow individuals to assess *habitat quality* (Box 1) and occupy the best possible habitats are expected to have evolved, on condition that environments are temporally autocorrelated (Doligez et al., 2003; Fretwell & Lucas, 1969; Johnson, 2007). Individuals looking for a breeding site should be attracted to *breeding patches* (Box 1) that they perceive as high‐quality ones, and therefore by high‐quality breeding sites that are likely already occupied or targeted by others, generating competition (Acker et al., 2017; Fretwell & Lucas, 1969; Lima & Zollner, 1996; Pulliam & Danielson, 1991). Consequently, non‐breeding could result from failure in the contest for dominance on a high‐quality site if individuals do not have enough time and energy to secure another —potentially lower quality— site or mate while competing for a high‐quality one (Bruinzeel, 2007; Danchin & Cam, 2002; Kokko et al., 2004). Non‐breeding could also result from queueing behaviour. Indeed, waiting for vacancy of a high‐quality site may offer better long‐term fitness prospects than breeding on a lower quality site (Ens et al., 1995; Zack & Stutchbury, 1992). Territorial competition for high‐quality breeding sites can therefore be hypothesized to contribute to shaping breeding propensity and hence contribute to population dynamics (Kokko & Sutherland, 1998; Newton, 1992), but empirical evidence is lacking.

Under this hypothesis, breeding propensity would vary over time with the intensity of competition for high‐quality sites in the population —which depends on the availability of disputed resources (high‐quality sites) and the number of competitors (individuals already occupying a site or looking for a site). Two predictions can be made regarding how breeding propensity is associated with two key population factors linked to competition for breeding sites: mean breeding habitat quality (hereafter ‘population habitat quality’) and number of conspecific individuals. (1) If increased population habitat quality occurs through increased habitat quality across multiple patches (i.e. decreased spatial heterogeneity and decreased variation in attractivity among patches), this would imply higher availability of high‐quality sites (whether they are occupied or not). Number of individuals being equal, the resulting competition decrease should be associated with increased breeding propensity. (2) If the number of individuals in the population increases, this would imply a higher number of competitors. Spatial heterogeneity of habitat quality being equal, the resulting competition increase should be associated with decreased breeding propensity.

Under prediction (1), we expect a positive relationship between mean reproductive success of breeders in the population (hereafter ‘population breeding success’, representing population habitat quality) and subsequent breeding propensity. Such a relationship would be detected while controlling for the confounding effect of the number of individuals in the population. This relationship could also result from spatially homogeneous temporal variation in environmental conditions —and thus in habitat quality— affecting the energetic cost of reproduction (e.g. food availability or weather conditions; Cayuela et al., 2018; Hoy et al., 2016; Nur & Sydeman, 1999). But this would contrast with situations where temporal variation in population habitat quality is spatially heterogeneous and where the prerequisite to prediction (1) is fulfilled: a tight negative relationship between population habitat quality and the degree of spatial heterogeneity of habitat quality. Under prediction (2), we expect a negative relationship between the numbers of individuals in the population and subsequent breeding propensity, that is negative density dependence in breeding propensity. Here again, such a relationship could also result from competition for food independent of competition for breeding sites. But if so, one would also expect competition for food to underlie a concomitant negative correlation between the number of individuals and population breeding success (e.g. Layton‐Matthews et al., 2019). By controlling for the confounding effect of population breeding success when testing for a negative relationship between the number of individuals and breeding propensity, one will thus detect the distinctive effect of competition for high‐quality breeding sites.

We tested our predictions in a population of black‐legged kittiwakes *Rissa tridactyla*, using 28 years of monitoring data of all active nests (~1000 each year) and capture‐resighting histories of >12,000 individuals. In this system, habitat selection for breeding at time *t* involves attraction to and intense competition for sites located in high‐quality patches, and individuals identify such patches via the reproductive success of conspecifics at the end of the previous breeding season *t* − 1 (Acker et al., 2017; Cadiou et al., 1994; Danchin et al., 1998; Appendix S1.2). We first quantified the relationship between the degree of spatial heterogeneity and the mean breeding success in the population to check the prerequisite to prediction (1) that population habitat quality is positively associated with the availability of high‐quality sites in the population. We then designed an integrated population model (‘IPM’) to jointly quantify fluctuations in population‐wide numbers of pre‐breeders, breeders, skippers and immigrants (‘breeding status’), their breeding propensity and population breeding success. We used IPM estimates to quantify the relationships between breeding propensity and numbers of competitors or population habitat quality, which allowed us to test predictions (1) and (2). More precisely, we assessed whether breeding propensity at *t* was positively correlated with population breeding success at *t* − 1 and negatively correlated with the numbers of breeders or non‐breeders at *t* − 1 (in each case, controlling for the other covariates using *partial correlations*, Box 1).

BOX 1 **Glossary**

*Breeding habitat selection*: The choice made by an individual to occupy a given breeding habitat. This choice typically involves the use of cues allowing an organism to assess habitat quality (e.g. conspecific breeding success). Such a choice may not be attained, for example if competitive inferiority prevents the individual from acquiring a breeding site.

*Breeding patch*: The space containing a contiguous set of breeding sites. Breeding patches can be considered at various spatial scales: for example, in kittiwakes, a patch can be a subpart of a cliff wall, an entire cliff wall, a cove consisting of several cliff walls or a colony consisting of contiguous coves.

*Breeding propensity*: The tendency of individuals to breed at a given occasion. In a population, breeding propensity is typically measured using breeding probability, independent of the patch where individuals will breed (since individuals may disperse between patches). For immigrants, it is represented by the proportion of individuals in the local population (the immigration rate), because the source population is unknown.

*Breeding site*: Space that is used by an individual (or a pair) to reproduce (e.g. in the kittiwake, where a pair builds a nest to lay eggs and rear chicks). It is also termed ‘breeding territory’ in species where individuals defend a delimited location against intruders.

*Habitat quality*: The expected fitness prospects offered to an individual by a given habitat (i.e. a breeding site or a breeding patch or the full set of patches in the population), and that varies according to spatio‐temporally heterogeneous factors (e.g. climate, vegetation, predation, food availability and parasitism). In temporally autocorrelated environments, it is best approximated by the preceding mean fitness of individuals in the habitat.

*Partial correlation*: Value of the correlation between two variables when other covariates are held constant in the sample (i.e. controlling for the confounding effect of the other covariates). If two processes corresponding to non‐mutually exclusive hypotheses are responsible for a relationship (e.g. the number of competitors and population habitat quality both influence breeding propensity), partial correlations allow a process to be detected while the other is also operating. This approach is not designed to discount the hypothesis corresponding to the process that is controlled for.

## 2 MATERIALS AND METHODS

### 2.1 Population monitoring

The data were collected in the Cap Sizun kittiwake population (48°03′N, 4°39′W; Brittany, France), where thousands of chicks have been individually marked with colour rings since 1979 (Appendix S1.1). Our analyses are based on data from 1985 to 2012. Monitoring was carried out throughout each breeding season by visiting all colonies weekly from first arrivals to the fledging period (January–June), and then daily until bird departures (July–August). During visits, the content of every nest site was recorded to determine breeding success, and the location and behaviour of ringed birds were recorded to determine breeding status (Cam et al., 1998). All fieldwork was licensed by the Centre de Recherches sur la Biologie des populations d’Oiseaux (‘CRBPO’, Muséum National d’Histoire Naturelle, Paris, France), and carried out in accordance with standard animal care protocols approved by the CRBPO

Resighting probability is virtually equal to one once an individual is recruited to the breeding population (age 3 at the earliest; Cam et al., 1998; see also Section 3). Whether they breed or not, the intensive resighting effort allows individuals that attend the breeding cliffs to be detected. Known‐age individuals are considered ‘pre‐breeders’ before their first breeding attempt in the population (recruitment), ‘breeders’ when they completed nest building in the current year (Cullen, 1957) or ‘skippers’ when they bred in the past but did not complete nest building in the current year. Pre‐breeders not always show up at the colonies in the breeding season, and those that attend the colonies may enter territorial contests for nest sites mainly at the end of the season. Skippers attend the breeding colonies, and their behaviour ranges from aterritorial floating to consistent territory holding throughout the season, including territorial contests for occupied and non‐occupied sites.

The breeding success of each nest was assessed using the number of chicks that reached at least fledging age (35 days or more). Breeding population was counted using the annual number of breeders, derived as twice the number of nests. Pairs very rarely build two nests successively; for marked individuals, successive nests were assigned to a unique pair.

### 2.2 Spatio‐temporal variation in habitat quality

The breeding habitat consists of multiple patches, which can be considered at various spatial scales: geographically distinct colonies (2–5, distant from each other by 0.5–12 km) composed of contiguous coves (5–18) including cliff walls (20–44; separated from each other by rocky ridges or coastal segments without nesting birds), themselves divisible into smaller heterogeneous patches (Acker et al., 2017; Bled et al., 2011; Danchin et al., 1998; Appendix S1.1,2). There is substantial among‐ and within‐patch heterogeneity in habitat quality, and this spatial heterogeneity is dynamic across years (Acker et al., 2017; Danchin et al., 1998; Appendix S1.1,2). At every spatial scale, patches are connected through large natal and breeding dispersal flows, which are typically directed from current low‐quality patches to high‐quality ones (Appendix S1.2).

During the study period, spatio‐temporal variation in breeding success is believed to have been mostly caused by predation on eggs by corvids and predation on young chicks by herring gulls in one colony, which eventually led to massive dispersal within the population and desertion of entire colonies (Acker et al., 2017; Cam et al., 2004; Danchin et al., 1998; Appendix S1.1,2). Ticks (*Ixodes uriae*) have also been suggested as a potential driver of variation in breeding success (Danchin et al., 1998). Food availability is unlikely to have caused the large spatial heterogeneity in breeding habitat quality that we observed, since kittiwakes feed on non‐defendable resources of which the availability varies at much larger spatial scales than within‐population foraging destinations (Christensen‐Dalsgaard et al., 2018; Oro & Furness, 2002; Suryan et al., 2002).

**FIGURE 1:**
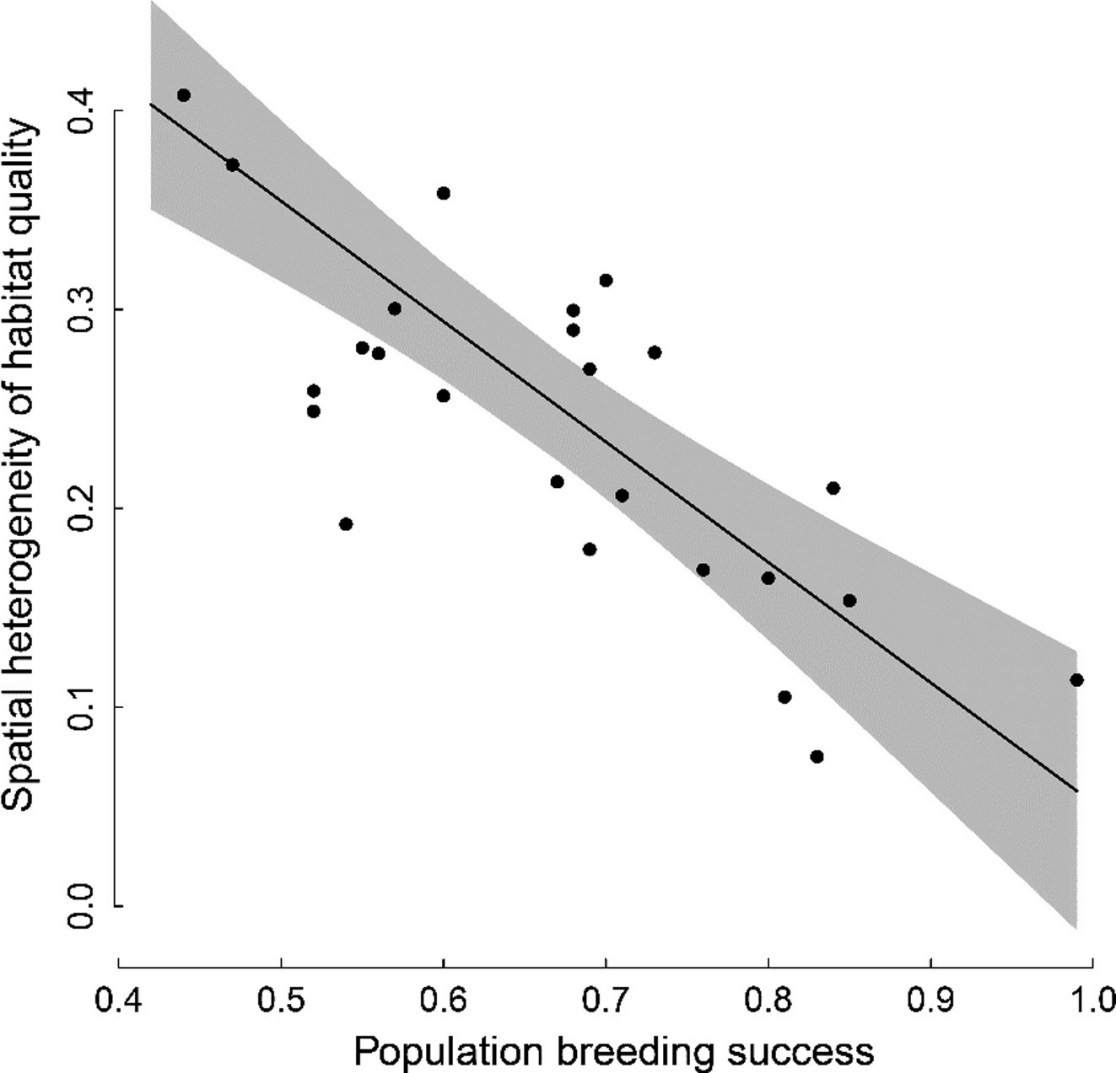
Relationship between the degree of spatial heterogeneity of habitat quality (measured by the Gini coefficient, at the cliff scale) and population breeding success (mean number of fledglings per nest). Grey background: 95% confidence interval of regression line

Prediction (1) relies on the prerequisite that increased population habitat quality arises from decreased spatial heterogeneity of habitat quality among patches, implying increased availability of high‐quality sites. To check this prerequisite, we evaluated spatial heterogeneity of habitat quality every year by inspecting the distribution of mean breeding success among patches (weighted by the number of sites occupied by breeders in each patch) and measuring its dispersion via the Gini coefficient (Appendix S1.3). We show our results at the cliff scale (we found similar patterns at the cove and colony scale, but no smaller scale was investigated; Appendix S1.3). Specifically, low Gini coefficients (low heterogeneity) corresponded to distributions packed around the mean, and high Gini coefficients (high heterogeneity) corresponded to years when a large proportion of patches had very low breeding success and only a few had high breeding success (Appendix S1.3). We found a strong negative correlation between the degree of spatial heterogeneity of habitat quality (measured by the Gini coefficient) and population breeding success (Figure 1; Pearson’s *r* = −0.79). Such a pattern demonstrates that our prerequisite is fulfilled.

### 2.3 Modelling population dynamics

To test the population‐level predictions from our hypothesis linking competition for high‐quality habitats to breeding propensity, we required robust quantification of numbers of individuals, breeding propensity in each breeding status and population breeding success. We developed an IPM (Schaub & Abadi, 2011; Schaub & Kéry, 2021) to model population dynamics from the joint analyses of population counts, individual resightings and breeding success observations. Such a model allows the estimation of key demographic parameters while fully propagating uncertainty across the different types of observations. Our IPM notably allowed estimating the numbers of immigrants, unmarked skippers and pre‐breeders that cannot be directly counted in the field or directly estimated from a single dataset.

**FIGURE 2:**
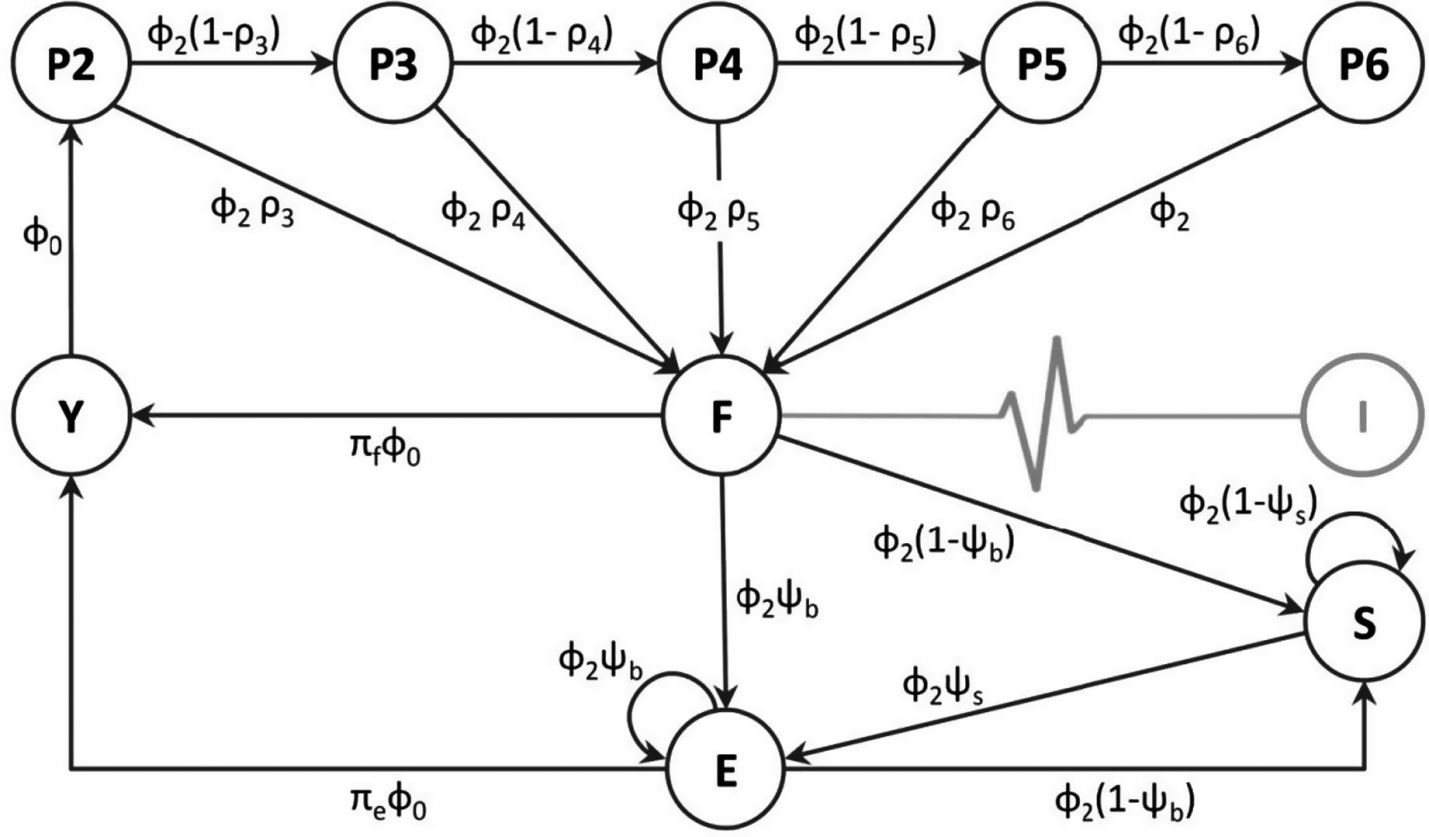
Kittiwake life cycle graph underlying the integrated population model. Life‐history states (black circles): yearlings (Y), pre‐breeders of age *i* (P*i*), first‐time breeders (F), experienced breeders (E) and skippers (S). Black arrows: state transitions; subscripts: transition rates. Demographic parameters: survival at age 0 and 1 ($\phi_{0}$) and from age 2 ($\phi_{2}$), recruitment rate at age *i* ($\rho_{i}$), breeding rate of former breeders ($\psi_{b}$) and former skippers ($\psi_{s}$), per capita breeding success of first‐time breeders ($\pi_{f}$) and experienced breeders ($\pi_{e}$). In grey is the annual pulse of immigrants (I) into first‐time breeders

The core of the IPM is a matrix population model (Caswell, 2001) depicting changes in the number of individuals in each state in year *t* as a function of demographic rates and the number of individuals in each state in year *t* − 1. We designed the life cycle (Figure 2) and population matrix (pre‐breeding census; Appendix S2.1) using prior knowledge of the population (Cam et al., 1998, 2002; Link et al., 2002). We defined nine life‐history states: yearlings, pre‐breeders of age 2, 3, 4, 5 and 6, first‐time breeders (locals and immigrants), experienced breeders and skippers (Figure 2). Demographic rates were modelled as time dependent. To account for demographic stochasticity, the number of individuals in each state was modelled using a Poisson or binomial distribution (Appendix S2.1).

We considered two age classes for survival probability (ϕ_0_ at age 0–1 and ϕ_2_ at age ≥ 2; Figure 2; Link et al., 2002). Given the complexity of the IPM, we made simplifying assumptions regarding heterogeneity in survival from age 2 to achieve reasonable computing times. We assumed equal survival of immigrants and locals, which is necessary because immigrants are not individually monitored. If this assumption does not hold, the estimated number of immigrants could be negatively (or positively) biased when immigrants have lower (or higher) survival than locals. We have no a priori hypothesis concerning this point, but there is no indication that major differences are likely (Appendix S2.2). Even if our estimates were systematically biased, temporal variation in the number of immigrants relative to the mean should still be correctly inferred, and derived relationships between immigration and demographic features would be properly assessed.

We considered four age classes for recruitment rate (ρ_*i*_, i.e. probability that a pre‐breeder at age *i* − *1* is a breeder at age *i*, *i* ∈ [[3, 6]], conditional on survival from *i* − *1* to *i*; Figure 2; Link et al., 2002). We assumed no recruitment before age 3 and after age 7 (recruitment rate at age 7 was fixed to 1), since no individual was ever recorded breeding at age 1, and we ignored the very few cases of recruitment at age 2 (~0.05% of individuals) and between ages 8 and 14 (~0.4%; Appendix S2.1). We considered status‐dependent breeding rates (Ψ_b_ and Ψ_s_, i.e. probability of breeding at *t*, respectively for individuals that bred and skipped breeding at *t‐1*; Figure 2; Cam et al., 1998).

We considered experience‐dependent per capita breeding success rates (i.e. half the number of fledglings produced in the nest) of first‐time breeders (π_f_; Figure 2) and experienced breeders whatever their number of previous breeding attempts (π_e_; Figure 2; Link et al., 2002). We assumed equal breeding success for immigrants and local first‐time breeders. This assumption has been shown to have a negligible impact on estimates of immigrant numbers and demographic rates in the common tern (*Sterna hirundo*), which has a very similar life cycle to the kittiwake (Szostek et al., 2014). This is expected because the population growth rate is not very sensitive to variation in fecundity parameters in long‐lived species (Caswell, 2001).

### 2.4 IPM datasets

**FIGURE 3:**
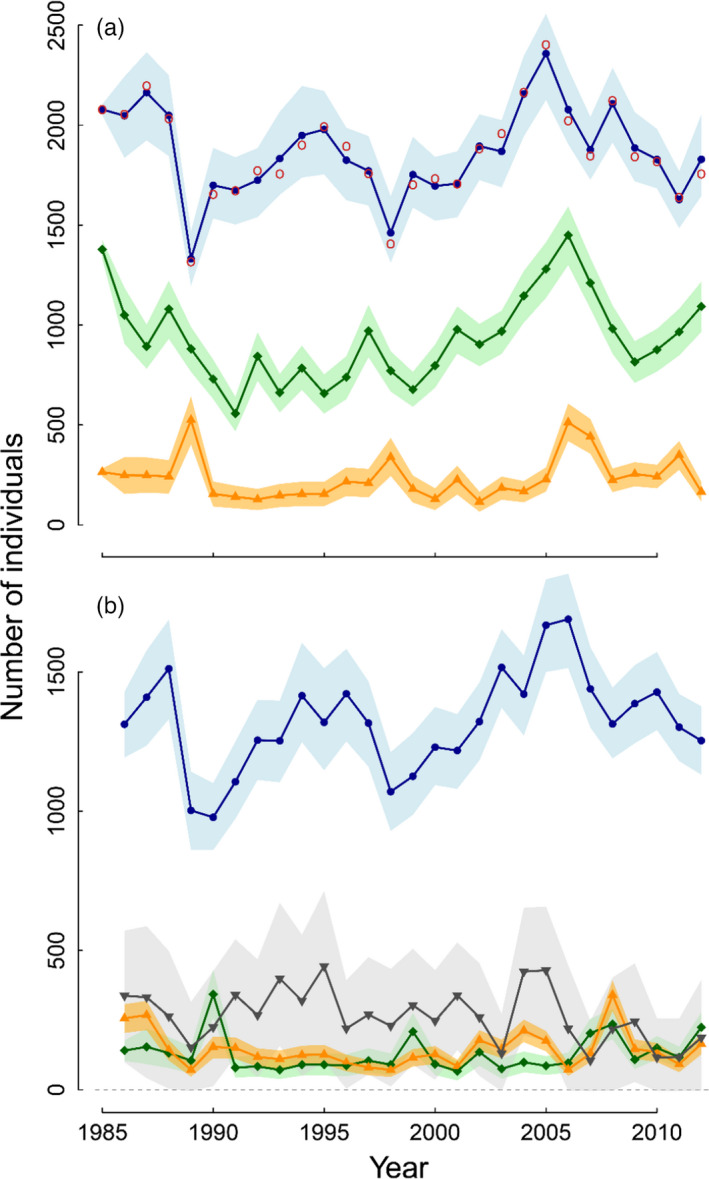
Population dynamics over 1985–2012. (a) Estimates of the numbers of pre‐breeders (orange triangles), skippers (green diamonds) and breeders (blue dots), and nest count data (red circles). (b) Breakdown of the numbers of breeders into immigrants (grey downward triangles), local first‐time breeders (orange upward triangles), former skippers (green diamonds) and former breeders (blue dots). Points: posterior means; shaded areas: 95%CRIs. In 1989, 2003, 2006–2008 and 2010–2012, 95%CRIs of the number of immigrants included negative values, suggesting that immigration may have been absent in these years

We analysed three datasets with the IPM: population count data, capture–recapture data and reproduction data. Population count data consisted of annual numbers of breeders, ranging 1316–2402 with large fluctuations (Figure 3a). Capture–recapture data consisted of capture‐resighting histories of ringed birds (12,091 individuals, of which 642 were marked in 1979–1984, before the period modelled here) indicating age and breeding status (pre‐breeder, breeder or skipper) at resighting. Reproduction data consisted of annual numbers of fledglings and corresponding numbers of nests belonging to pairs of either (a) first‐time breeders (both mates ringed, 1962 breeding attempts), (b) experienced breeders (both mates ringed, 8785 breeding attempts) or (c) a first‐time breeder mated with an experienced breeder (both ringed) or at least one unringed mate (25,366 breeding attempts).

### 2.5 Likelihood

The IPM likelihood is the product of likelihoods of three models for the three datasets, assuming independence between datasets. In practice, this assumption of independence is not completely fulfilled, but simulations have shown that its violation has a very limited effect on parameter estimates (Schaub & Fletcher, 2015; Weegman et al., 2021). The likelihood given the population count data was formulated using a state‐space model (Appendix S2.3). The state process was defined by the matrix population model in which fluctuations in class‐specific population sizes are described. We assumed a log‐normal distribution for the observation process with time‐independent standard deviation. The likelihood given the individual capture‐resighting histories was formulated using a state‐space formulation of a multistate capture–recapture model (Appendix S2.4). We assumed different time‐varying resighting rates for yearlings and pre‐breeders, equal and temporally constant resighting rates for breeders and skippers, and no error in state assignment at resighting (Cam et al., 1998, 2002). The likelihood given the reproduction data was formulated using three Poisson regressions for fledgling numbers as a function of the number of nests and experience‐dependent breeding success (Appendix S2.5). The three regressions were for pairs of (a) first‐time breeders, (b) experienced breeders and (c) individuals of unknown or different categories of experience. For the latter, we ignored pair characteristics and assumed that their breeding success rate was the population breeding success, that is mean breeding success rate of inexperienced and experienced breeders weighted by their proportions among breeders.

### 2.6 Inference and model assessment

To estimate model parameters, the joint likelihood was analysed in the Bayesian framework. We specified vague prior distributions with reasonable bounds for all parameters (Appendix S2.6). We used the uniform distribution over [−5 to 1,000] as prior for the number of immigrants; the inclusion of negative values enables testing whether there is immigration at all (Schaub & Fletcher, 2015). We performed Markov Chain Monte Carlo simulation for posterior sampling using JAGS 3.4.0 (Plummer, 2003; model code and full details of sampling are in Appendices S2.7 and S3.1 respectively). While the capture–recapture data had already been analysed with similar multistate model structures (e.g. Cam et al., 1998, 2002; Link et al., 2002; yielding similar estimates, see Section 3), the additional analysis of population counts and reproduction data allowed estimating parameters that had not yet been estimated. We used posterior predictive checks (Gelman & Hill, 2006) to evaluate the fit of our IPM to the population count data and the reproduction data (Appendix S4). Overall, these checks indicated a good fit (Appendix S4).

### 2.7 Derived quantities

We derived the posterior distribution of key quantities from model parameters, synthesizing compound biological effects of interest while accounting for their uncertainty (Appendix S5). Specifically, to characterize population dynamics with respect to breeding propensity, we derived the breeding population growth rate as the number of breeders in year *t* divided by the number of breeders in year *t* − 1 (Appendix S5.4). To characterize population composition, we derived the among‐breeder proportions of former breeders (individuals that bred at *t* − 1), former skippers (individuals that skipped breeding at *t* − 1), local first‐time breeders and immigrants. To synthesize the breeding propensity of all pre‐breeders, we derived the age‐independent ‘integrative recruitment rate’, that is the proportion of first‐time breeders at *t* among the individuals of all age classes (3 to 6) alive and available for recruitment in the current year *t* (i.e. that have never bred before). Breeding propensity of immigrants was represented by the immigration rate, that is the proportion of immigrants among breeders in the current year (note that similar results were obtained using the absolute number of immigrants; Appendix S5.4). We also derived the number of non‐breeders (i.e. pre‐breeders plus skippers) present at the breeding colonies by correcting the number of non‐breeders in the population by their resighting rate (Appendix S5.3).

### 2.8 Correlates of demographic features and test of hypothesis regarding breeding propensity

Before testing our predictions, and to place our working hypothesis in the general demographic context, we assessed the contribution of breeding propensity at *t* of each breeding status at *t* − 1 to population dynamics. Specifically, we assessed the contributions of demographic rates to population fluctuations using estimates from the IPM. We derived posterior distributions of partial correlations between breeding population growth rate and survival rate, breeding rates of former breeders and former skippers, integrative local recruitment rate and immigration rate (while controlling for each other’s effects; Appendix S5.4; Szostek et al., 2014). Because information was insufficient in the first year to properly estimate the number of individuals that could not be counted in the field, we considered all parameters from the second year onwards to estimate partial correlations.

We then tested the population‐level predictions of our hypothesis linking competition for high‐quality habitats to breeding propensity using estimates from the IPM. We derived posterior distributions of partial correlations to test for relationships between breeding propensity (at *t*), and population habitat quality as well as abundance of competitors (at *t* − 1; Appendix S5.4). We assessed the relationship between breeding propensity at *t* in each breeding status (i.e. breeding rates of former breeders and former skippers, integrative recruitment rate and immigration rate) and population breeding success, number of breeders or number of present non‐breeders at *t* − 1 (while controlling for each other’s effects). We assessed the evidence for a partial correlation by computing the proportion of its posterior distribution that had the same sign as its posterior mean (‘*P*’). Values of *P* close to 1 indicate strong evidence for a correlation with a given sign, while values close to 0.5 indicate no clear evidence (i.e. similar evidence for a negative or positive correlation).

## 3 RESULTS

The estimates of breeding population size from the IPM closely matched the population count data (Figure 3a). Detailed posterior summaries of IPM parameters and derived quantities are given in Appendices S3 and S5 respectively. Hereafter, estimates are reported as the posterior mean with 95% credible interval (‘95%CRI’) in brackets.

### 3.1 General demographic context

**FIGURE 4:**
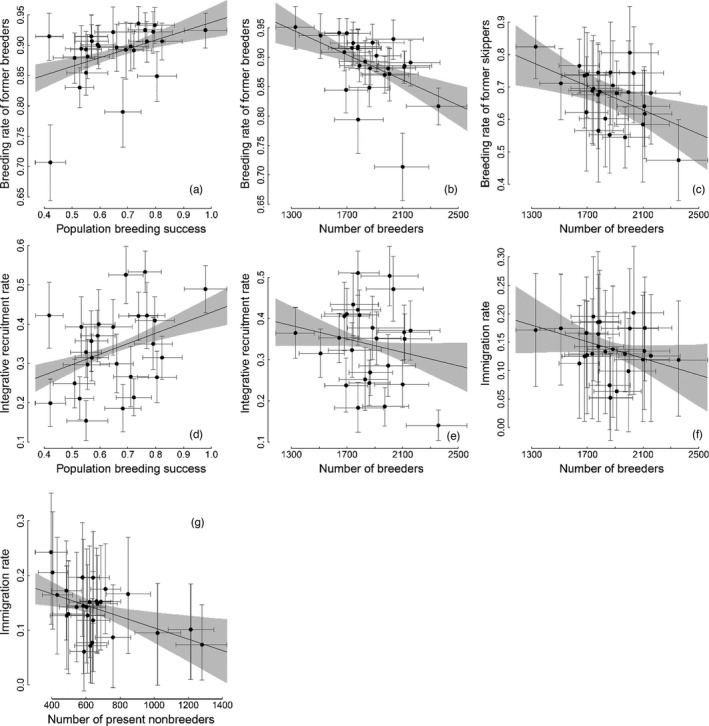
Associations between breeding propensity in year *t* (*y*‐axis) and key population features in year *t* − 1 (*x*‐axis). Different breeding propensities were considered depending on the individual’s status in year *t* − 1: breeding rate for former breeders (a, b) and skippers (c), integrative recruitment rate for pre‐breeders (d, e) and the immigration rate for immigrants (f, g). The key population features considered were population breeding success (mean number of fledglings per nest (a, e)) and number of competitors (number of breeders, b, c, d, f, or number of present non‐breeders, g). Relationships presented here are those with strong evidence for a positive or negative partial correlation (Table 1; see Appendix S5.4 for other relationships). These are partial residual plots representing partial correlations controlling for the remaining population features (e.g. in panel (a) the partial correlation between the integrative recruitment rate and number of breeders controls for population breeding success and the number of present non‐breeders); residuals were centred on the variable mean to rescale variation within the original range. Points: posterior means of rescaled residuals; segments: 95%CRIs. Solid line: posterior mean of regression line; grey background: 95%CRI

At the scale of the study period, breeding population size was stationary or nearly so (average growth rate: 1.001 [0.999, 1.004]; Appendix S5.1), despite large annual fluctuations (Figure 3a). The estimates of the demographic rates were consistent with those reported in previous studies not using an IPM (e.g. Cam et al., 1998, 2002; Link et al., 2002). Mean breeding success across years was 0.16 [0.14, 0.19] fledglings per capita for first‐time breeders and 0.36 [0.33, 0.40] fledglings per capita for experienced breeders, resulting in population breeding success of 0.65 [0.64, 0.66] fledglings per nest, with large annual fluctuations indicating pronounced temporal variability in population habitat quality (Appendix S3.2; Figure 4). Mean local survival probability was 0.65 [0.59, 0.71] at ages 0 and 1, and 0.81 [0.78, 0.83] afterwards. Mean resighting probability, indicative of presence at the breeding grounds, was 0.05 [0.04, 0.07] for yearlings, 0.81 [0.78, 0.84] for older pre‐breeders and 0.998 [0.997, 0.999] for recruited individuals. Mean recruitment rate at ages 3, 4, 5 and 6 was 0.13 [0.08, 0.18], 0.41 [0.34, 0.47], 0.53 [0.48, 0.59] and 0.67 [0.58, 0.76] respectively. The resulting mean integrative recruitment rate (i.e. breeding propensity of pre‐breeders) was 0.34 [0.33, 0.35]. Mean breeding rate was 0.90 [0.87, 0.92] for former breeders and 0.69 [0.62, 0.75] for former skippers. These breeding propensities were clearly lower than 1, and highly variable across years (Appendix S3.2; Figure 4), indicating the demographic importance of breeding propensity.

### 3.2 Population composition

Among local individuals (i.e. locally born or already established in the population), there was a prominent proportion of breeders (1985–2012 mean: 62.1% [61.3, 62.8]), a moderate proportion of pre‐breeders (30.2% [29.4, 31.0]) and a small proportion of skippers (7.7% [7.2, 8.3]), with large fluctuations (Figure 3a). There was a high turnover among breeders, with a mean of 30% of current breeders that had not bred in the population in the previous year (Figure 3b). Across years, the breeding population was composed on average of 7.6% [7.2, 8.0] local first‐time breeders, 7.0% [6.5, 7.5] former skippers and 14.0% [12.9, 15.0] immigrants, versus 71.4% [70.4, 72.3] former breeders (Figure 3b). These results highlight how status‐dependent breeding propensity shaped the highly dynamic compositions of the breeding and non‐breeding segments of the population.

### 3.3 Contribution of breeding propensity to population dynamics

The partial correlation with breeding population growth rate was 0.59 [0.29, 0.87] for immigration rate, 0.56 [0.36, 0.75] for breeding propensity of former breeders, 0.32 [0.05, 0.59] for breeding propensity of former skippers and 0.08 [−0.20, 0.36] for the integrative recruitment rate. For comparison, this partial correlation was 0.47 [0.27, 0.67] for local survival probability from age 2, that is the rate responsible for permanent disappearance of individuals from the breeding population. These values indicate that breeding propensity in all statuses except pre‐breeders had non‐negligible effects on temporal variation in breeding population growth, and these effects were especially high (and higher than the effect of survival) for immigrants and former breeders.

### 3.4 Correlates of breeding propensity

We found positive associations between population habitat quality and breeding propensity in former pre‐breeders and breeders, but not in former skippers and immigrants (Table 1; Figure 4). There was evidence of positive partial correlations between population breeding success in year *t* − 1 and both the breeding rate of former breeders in year *t* and the integrative recruitment rate (Table 1; Figure 4a,d). We also found negative associations between the numbers of competitors and breeding propensity (independently of population habitat quality) in all breeding statuses, with some status‐dependent modulation in strength (Table 1; Figure 4). This is shown by negative partial correlations between the number of breeders at *t* − 1 and the breeding rates of former breeders and skippers, the integrative recruitment rate and the immigration rate at *t*  — which was of lower magnitude for the latter two (Table 1, Figure 4b,c,e,f). There was also evidence of a negative partial correlation between the immigration rate at *t* and the number of non‐breeders (pre‐breeders plus skippers) present at *t* − 1 (Table 1; Figure 4g).

**TABLE 1:** Summary of the associations between status‐specific breeding propensity (rows) and key population features: population breeding success or numbers of competitors (columns)

Breeding propensity (year *t*)		Population feature (year *t* − 1)		
Former status (year *t* − 1)	Parameter (year *t*)	Population breeding success	Number of breeders	Number of present non‐breeders
Breeder	Breeding rate of former breeders	**0.38 [0.21, 0.55] (1.00)**	**−0.46 [−0.64, −0.27] (1.00)**	0.00 [−0.18, 0.18] (0.51)
Skipper	Breeding rate of former skippers	−0.09 [−0.39, 0.21] (0.72)	**−0.40 [−0.64, −0.15] (1.00)**	−0.11 [−0.37, 0.15] (0.80)
Pre‐breeder	Integrative recruitment rate	**0.34 [0.20, 0.48] (1.00)**	**−0.18 [−0.37, 0.01] (0.97)**	−0.04 [−0.18, 0.10] (0.72)
Immigrant	Immigration rate	0.07 [−0.21, 0.35] (0.70)	**−0.26 [−0.52, 0.01] (0.96)**	**−0.35 [−0.59, ‐0.09] (0.99)**

*Notes*: coefficients are partial correlations controlling for the confounding effect of the remaining population features (e.g. the partial correlation between immigration rate and population breeding success controls for the number of breeders and number of present non‐breeders). Estimates are posterior means with 95%CRI between brackets, and the proportion of the posterior distribution that had the same sign as the posterior mean (*P*) between parentheses. Relationships with strong evidence of correlation with a given sign (*P* > 0.95) are highlighted in bold.

## 4 DISCUSSION

Reproduction in animals is often contingent on acquisition or retention, or simply access to a breeding site (except when fertilization is external, or in non‐territorial species). Accordingly, we hypothesized that habitat selection processes, including attraction to and competition for high‐quality breeding sites, could influence whether individuals will breed or not. Given the finite availability of high‐quality sites, we predicted that larger numbers of competitors generate lower breeding propensity due to competition for breeding sites, regardless of temporal variation in population habitat quality. We also predicted that higher habitat quality across a population, if realized through greater availability of high‐quality sites across patches, relaxes competition in each high‐quality patch, generating higher breeding propensity. Our integrated population model applied to long‐term kittiwake monitoring data allowed us to evidence relationships that match these predictions, in addition to demonstrating the critical role of breeding propensity for population growth and composition. Overall, in complement to standard energy‐cost views on the achievement of reproductive careers, our study sheds light on the importance of competition for high‐quality sites in shaping breeding propensity, individual life histories and population dynamics.

Previous studies have used the occurrence of delayed or skipped breeding and territorial behaviour in heterogeneous habitats to hypothesize that intense competition for high‐quality breeding sites can drive non‐breeding (Zack & Stutchbury, 1992), implying density dependence in breeding propensity (Kokko & Sutherland, 1998). This is corroborated by many studies that have shown that non‐breeding is associated with subordination in territorial contests for high‐quality breeding sites, from behavioural observations to experiments in taxa spanning fish, reptiles, birds, mammals and arthropods (e.g. Baird & Timanus, 1998; Gołąb et al., 2013; Kokko et al., 2004; Newton, 1992; Piper et al., 2000; Stiver et al., 2006; Wauters & Lens, 1995). Other studies have matched age dependence in recruitment patterns with expectations of adaptive queuing for high‐quality sites (Ens et al., 1995; van de Pol et al., 2007). While no empirical studies investigating the role of competition for high‐quality sites in breeding propensity have previously demonstrated negative density dependence in breeding propensity, such a mechanism has been suggested by several studies that found high recruitment rates subsequent to high adult mortality (e.g. Porter & Coulson, 1987; Pradel et al., 1997; Sæther et al., 2002; Votier et al., 2008). Our study unifies and generalizes these previous findings by providing evidence of negative density dependence in breeding propensities (Table 1; Figure 4), and highlights the importance of competition in shaping breeding propensity at the population level.

In general, negative density dependence of breeding propensity can be mediated through competition for other resources than breeding space, namely food resources. This is an inherent part of competition for breeding sites when food resources are spatially heterogeneous and defended in the breeding territory (e.g. Aho et al., 1999; Ens et al., 1995; Wauters & Lens, 1995). Alternatively, the link between competition for food resources and for breeding habitat is loose or inexistent when food resources are limited but non‐defendable (as in central place foragers such as kittiwakes) or spatially homogeneous across the breeding habitat. Nonetheless, if food is a limiting resource for which individuals compete independently of the breeding site, density should also be negatively associated with population breeding success (e.g. Arcese & Smith, 1988; Layton‐Matthews et al., 2019; Wauters et al., 2004). Here we controlled for the effect of population breeding success when estimating the correlation between the number of competitors and breeding propensity (Table 1; Figure 4), which is why the observed relationship is to be explained by competition for breeding habitats, not for food.

The energetic requirements of reproduction and food intake remain a major potential determinant of breeding propensity. And indeed, it has been shown that improved environmental conditions implying lower energetic demand or simply increased food availability are associated with both increased breeding propensity and breeding success (e.g. Hoy et al., 2016; Nur & Sydeman, 1999). Following this view, previous studies documenting positive relationships between population habitat quality and subsequent breeding propensity have referred to physiological condition or perceived chances to overcome reproductive costs (e.g. Cayuela et al., 2018; Frederiksen & Bregnballe, 2001). However, competition for breeding sites on its own can also generate a positive association between population breeding success and breeding propensity, as found in our study (Table 1; Figure 4). Increased mean population breeding success can reflect increased habitat quality in multiple patches across the population, which results in decreased spatial heterogeneity of habitat quality across space (Figure 1; Appendix S1) and decreased competition for high‐quality sites. Competition for high‐quality breeding sites would appear to better explain temporal variation in breeding propensity than energetic requirements in systems where temporal variation in habitat quality is spatially heterogeneous rather than homogeneous. Where possible in the future, the relative importance and joint contribution of these two explanations could be addressed by analyses that explicitly distinguish between these two forms of variation (e.g. using unambiguous measures of food availability or experiments relying on supplementary feeding).

Spatial heterogeneity of the environment, attraction to high‐quality habitats and competition for breeding space are commonplace in animal taxa and are the basis of theory on spatial distribution of individuals (Fretwell & Lucas, 1969; Pulliam & Danielson, 1991). Our hypothesis linking habitat selection mechanisms and breeding propensity should thus be of general relevance, but its importance should depend on key factors underlying competition intensity. For example, the use of information on habitat quality emanating from conspecifics (e.g. their breeding success) is a common habitat selection mechanism that necessarily makes individuals aggregate and covet the same sites, enhancing competition (Danchin et al., 1998; Doligez et al., 2003; Schmidt et al., 2010). However, such a process depends on predictability and spatial heterogeneity in habitat quality (Acker et al., 2018; Doligez et al., 2003): the more predictable (i.e. temporally autocorrelated) and heterogeneous the habitats (i.e. stronger site‐dependent differences in fitness prospects), the higher the competition for high‐quality breeding sites. The strength of competition will also depend on the degree to which the availability of high‐quality sites is limited. The limitation as perceived by individuals will be conditioned by the type of information used to assess habitat quality and the overall strategy for habitat search (Acker et al., 2017; Lima & Zollner, 1996; Piper, 2011; Rushing et al., 2021; Schmidt et al., 2010), as well as by any physical limitation in the number of breeding sites (Kokko & Sutherland, 1998). Further, competition has led to the evolution of territorial behaviour characterized by costly defence and active contests for exclusive space suitable for breeding (Adams, 2001; Stamps, 1994). By modulating the benefits of occupying a high‐quality site versus a low‐quality one through related costs of site acquisition and retention in face of competitors, key features of territorial behaviour like territory size and reducibility or risk of injury should modulate the influence of competition for high‐quality sites on breeding propensity (Kokko & Sutherland, 1998; López‐Sepulcre & Kokko, 2005).

Our study system provides a valuable example of the behavioural and environmental characteristics leading to strong competition for high‐quality sites and of its consequences for breeding propensity. Several studies have shown that kittiwakes breed in spatio‐temporally heterogeneous but predictable habitats, use public information to target high‐quality habitats at all spatial scales, devote substantial time and energy to acquiring and defending breeding sites and show positive associations between breeding propensity and competitive behaviour claiming territorial dominance (Acker et al., 2017; Aubry et al., 2009; Boulinier et al., 2008; Cadiou et al., 1994; Cam et al., 2002; Danchin et al., 1998; Appendices S1.2 and S5.5). Kittiwakes feed on non‐defendable resources that vary at regional scales, and although food availability can affect their reproductive success (Frederiksen et al., 2005; Golet et al., 2004; Suryan et al., 2002), previous studies have not found evidence of effects of food availability on breeding propensity (Golet et al., 2004; Oro & Furness, 2002) or of density dependence mediated by food limitation (Frederiksen et al., 2005). Studies of breeding propensity in other taxa would be valuable to further clarify the role that competition for high‐quality breeding sites could play in shaping breeding propensity.

In our study population, competitive asymmetries among individuals in different breeding status likely modulate how competition for high‐quality sites influences breeding propensity. In general, the most competitive individuals are assumed to occupy the highest quality habitats and force others to settle in lower quality habitats (Fretwell & Lucas, 1969; Pulliam & Danielson, 1991) or to skip breeding (Ens et al., 1995; Piper et al., 2000). Our results suggest that breeders and skippers might benefit from a lower number of breeding competitors in the population to a greater extent than pre‐breeders and immigrants, and that immigrants might be the only status affected by competition with non‐breeders (Table 1; Figure 4). This probably reflects the lack of behavioural maturity of pre‐breeders compared to experienced individuals, which would lead to inferiority of many pre‐breeders under any competitive intensity (Aubry et al., 2009; Cam et al., 2002), and to an even greater inferiority of immigrants due to their lack of knowledge and familiarity with the local competitive context (e.g. Germain et al., 2017). Our results suggest that decreased competition for high‐quality sites when population breeding success increased benefited pre‐breeders and breeders, while this was not clear for skippers and immigrants (Table 1; Figure 4). This could be because skippers and immigrants tend to target less attractive sites (located in patches of lower quality; e.g. Bruinzeel, 2007) where their chances of acquiring a site are not (or less) impacted by variation in the availability of high‐quality sites across the population.

Overall, the process of competition for high‐quality breeding sites emphasized here may explain some major variations in individual life histories. Through despotism exercised by some individuals that manage to breed in high‐quality habitats, less competitive ones are forced to poorer reproductive careers (e.g. Bruinzeel, 2007; van de Pol et al., 2007). In our study population, out‐competed kittiwakes could skip breeding opportunities (Cadiou et al., 1994; Cam et al., 2002) or access lower quality breeding sites (Aubry et al., 2009), where they are likely to fail and then disperse to avoid failing again (Acker et al., 2017), re‐enter competition to obtain a new site and repeat this cycle (‘the spiral of failure’; Cam et al., 2004, 2013). However, our results suggest that higher population habitat quality or lower density may soften competition by offering better breeding opportunities or enhanced access to good opportunities. The same mechanisms should also affect population dynamics. The positive association between previous population breeding success and breeding propensity should accentuate the impacts of temporal variation in habitat quality on population growth (Brown et al., 2000; Danchin et al., 1998). Yet, given the negative association between breeding propensity and the number of competitors, the impact of habitat quality is likely to be counteracted by the variation in competition intensity arising from breeding density changes. These results open valuable future opportunities to evaluate the relative importance of competition for high‐quality breeding sites in amplifying or buffering population dynamics via breeding propensity.

## CONFLICT OF INTEREST

The authors have no conflict of interest to declare.

## AUTHORS' CONTRIBUTIONS

P.A. and E.C. formulated the ideas; J.‐Y.M. conceived the monitoring study; E.C. and J.‐Y.M. collected the data; P.A. and M.S. designed the modelling methodology; P.A. analysed the data and led the writing of the manuscript, assisted by E.C. and A.B. All authors contributed critically to the drafts and gave final approval for publication.

## Supporting information


Appendix S1 to S5

## Data Availability

Data are available from the Zenodo Repository https://doi.org/10.5281/zenodo.6009808 (Acker et al., 2022).
