# Supplementary material for: Can attraction to and competition for high‐quality habitats shape breeding propensity?
Source: J Anim Ecol. 2022 Mar 11;91(5):933–45. doi: 10.1111/1365-2656.13676 (PMC9314844; doi:10.1111/1365-2656.13676)
Supplement: Supplementary file 1 — Appendix S1 to S5 [file JANE-91-933-s001.docx]

## Supporting information to:

**Can attraction to and competition for high-quality habitats shape breeding propensity?**

Paul Acker^1,2,3,^*, Michael Schaub^4^, Aurélien Besnard^3^, Jean-Yves Monnat^5^, Emmanuelle Cam^2,6^

^1^ Centre for Biodiversity Dynamics, Institutt for Biologi, NTNU, Trondheim, Norway

^2^ Laboratoire EDB (UMR 5174), Université Paul Sabatier – CNRS – IRD; 118 route de Narbonne, F-31062 Toulouse, France

^3^ CEFE, Univ Montpellier, CNRS, EPHE-PSL University, IRD, Montpellier, France

^4^ Swiss Ornithological Institute; CH-6204 Sempach, Switzerland

^5^ 6 Pennarun d'An Traon, F-29770 Goulien, France

^6^ Univ Brest, CNRS, IRD, Ifremer, LEMAR, F-29280 Plouzané, France

* Corresponding author; [paul.acker@ntnu.no](mailto:paul.acker@ntnu.no)

**Contents:**

**Appendix S1: Details of spatial-temporal variability in habitat quality (p. 2)**

**Appendix S2: Details of the integrated population model (p. 18)**

**Appendix S3: Summaries of the posterior distributions (p. 36)**

**Appendix S4: Posterior checks (p. 44)**

**Appendix S5: Specification and results of the derived analyses (p. 54)**

## APPENDIX S1

## Details of spatio-temporal variability in habitat quality

**Contents:**

**S1.1 History of the kittiwake population (p. 2)**

**S1.2 Spatio-temporal heterogeneity of habitat quality (p. 5)**

**S1.3 Relationship between spatial heterogeneity and population breeding success (p. 8)**

**S1.4 References (p. 15)**

### S1.1 History of the kittiwake population

Kittiwakes have been ringed in the Cap Sizun since 1979 (Fig. S1). Birds are ringed using three small colour DARVIC-plastic rings on one leg, and two colour rings along with one individually-coded inscribed metal ring on the other leg. For the plastic rings, we used eight possible colours that are highly distinguishable, allowing a very large number of ordered combinations to generate an individual colour code for each ringed bird. There is no issue of mark loss. Colours can be a bit washed after a while, but observers have always been able to distinguish them (there is no “worn” unreadable mark such as happens with inscribed alphanumeric colour rings). It happens in rare cases that individuals lose one ring, but these ones could be verified by checking their metal rings, and they have always continued to be individually identifiable with their remaining colours rings that still formed a unique colour combination within the population. Further, the metal ring is virtually unlosable, and when an individual is seen bearing a metal ring only, we carry on specific effort to read the inscribed individual code; this has concerned a handful of individuals since 1979, that were all immigrants.

At the beginning of the monitoring program, the study area hosted 4 colonies, which were relatively close to one another (colony 1, 2, 3 and 4, in or near the nature reserve of Goulien; Fig. S1) but only colony 1 was subject to intensive survey and individual monitoring. In 1981 the program was extended to colony 3, and then to colony 2 and colony 4 in 1983 (Fig. S1, S2). A few individuals colonized the Pointe du Raz in 1982, thus establishing colony 5 (Fig. S1, S2) which was included in the program in 1984 and which is still intensively monitored today. The number of nests in colony 5 first increased (1984−1987) then declined towards quasi-extinction (1988−1991) and finally increased to concentrate most of the breeding population. At the same time, the other colonies were progressively deserted (Fig. S2) and a “new” colony was established between Goulien and the Pointe du Raz (colony 6). There are historical records of presence of kittiwakes in colony 6 before the study started (Guermeur & Monnat, 1980). The formerly largest colony (colony 1) went extinct in 1999. A pioneer pair built a complete nest in the Pointe du Van in 2004, thus re-establishing colony 6 (Fig. S1, S2) which was included into the monitoring program. Colony 2 went extinct in 2008, colony 3 and colony 4 contained very few nests in 2012 (17 and 8, respectively) and went extinct in 2013 (Fig. S2). Additional information concerning kittiwake repartition and dynamics in France between 1960 and 2000 (with further details concerning the history of the study population in Brittany) can be found in Guemeur & Monnat (1980), Cadiou (1993) and Monnat & Cadiou (2004). Further details on the associated dynamics at smaller spatial scales are available in Acker et al. (2017).


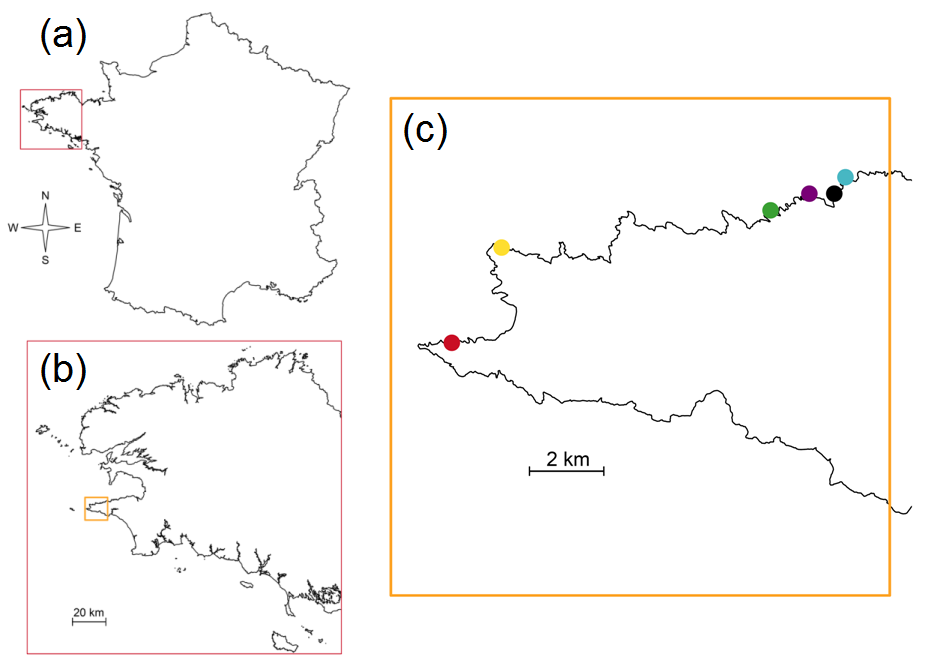


**Figure S1**. Location of the study area and colony sites. (a) The study population is located in Brittany, northwestern France (red square), (b) in the Cap Sizun (orange square). Dots indicate colony sites (c): colony 1 in green, colony 2 in purple, colony 3 in black, colony 4 in blue, colony 5 in red and colony 6 in yellow.

**
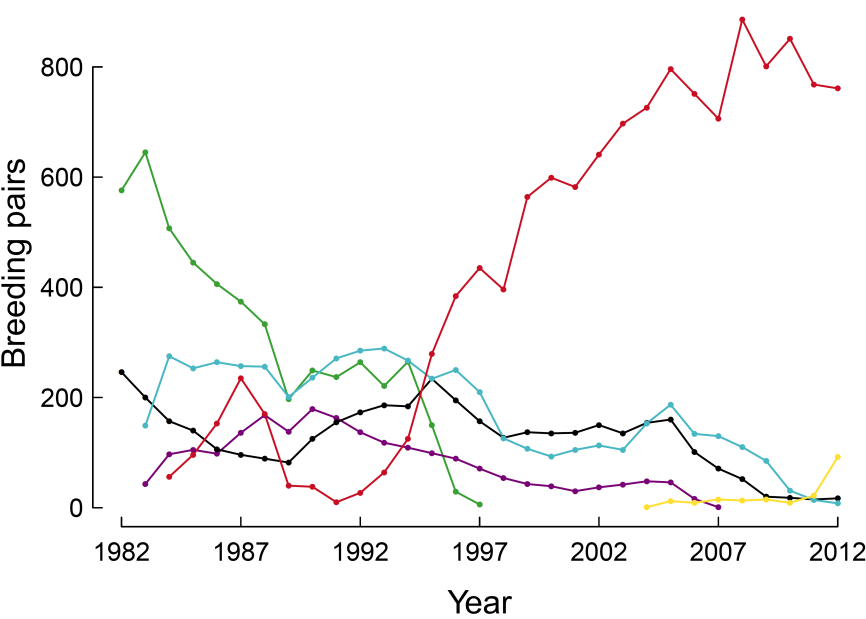
**

**Figure S2.** Size of the colonies across 1982-2012. Colony size is expressed in number of breeding pairs, which was approximated by the number of nests that have reached the completion criterion (see *Materials and Methods*). Each time series starts in either 1982 or at the beginning of the monitoring in the given colony. The size is not plotted for colony sites once they have gone extinct. Colony 1 is plotted in green, colony 2 in purple, colony 3 in black, colony 4 in blue, colony 5 in red and colony 6 in yellow.

### S1.2 Spatio-temporal heterogeneity of habitat quality and within-population dispersal

Our hypotheses relating competition for high-quality sites to breeding propensity are valid in the context of spatio-temporal heterogeneity of the environment (with some degree of predictability), and hence spatio-temporal heterogeneity of reproductive prospects that define habitat quality. In our study system, previous publications have demonstrated and detailed heterogeneity (and predictability) of habitat quality among spatial units at multiple spatial scales: colonies, coves, cliffs, smaller spatial units within cliffs, and nests (see Danchin et al., 1998; Aubry et al., 2009; Bled et al., 2011; Acker et al., 2017). As mentioned in *Methods*, this heterogeneity implied large variations in mean breeding success per nest among habitat patches at all spatial scales (see details in publications cited above). Here we illustrate this variation at the cliff scale, which is the smallest scale at which a detailed description of spatial heterogeneity in the quality of habitat patches is manageable, and which has proven relevant to depict fine habitat selection mechanisms underlying the distribution of individuals within the population (Danchin et al., 1998; Aubry et al., 2009; Acker et al., 2017). We provide graphs of temporal variation of habitat quality expressed as the mean breeding success (fledglings per nest) by patch (cliffs within colonies) across the study period (Fig. S3).


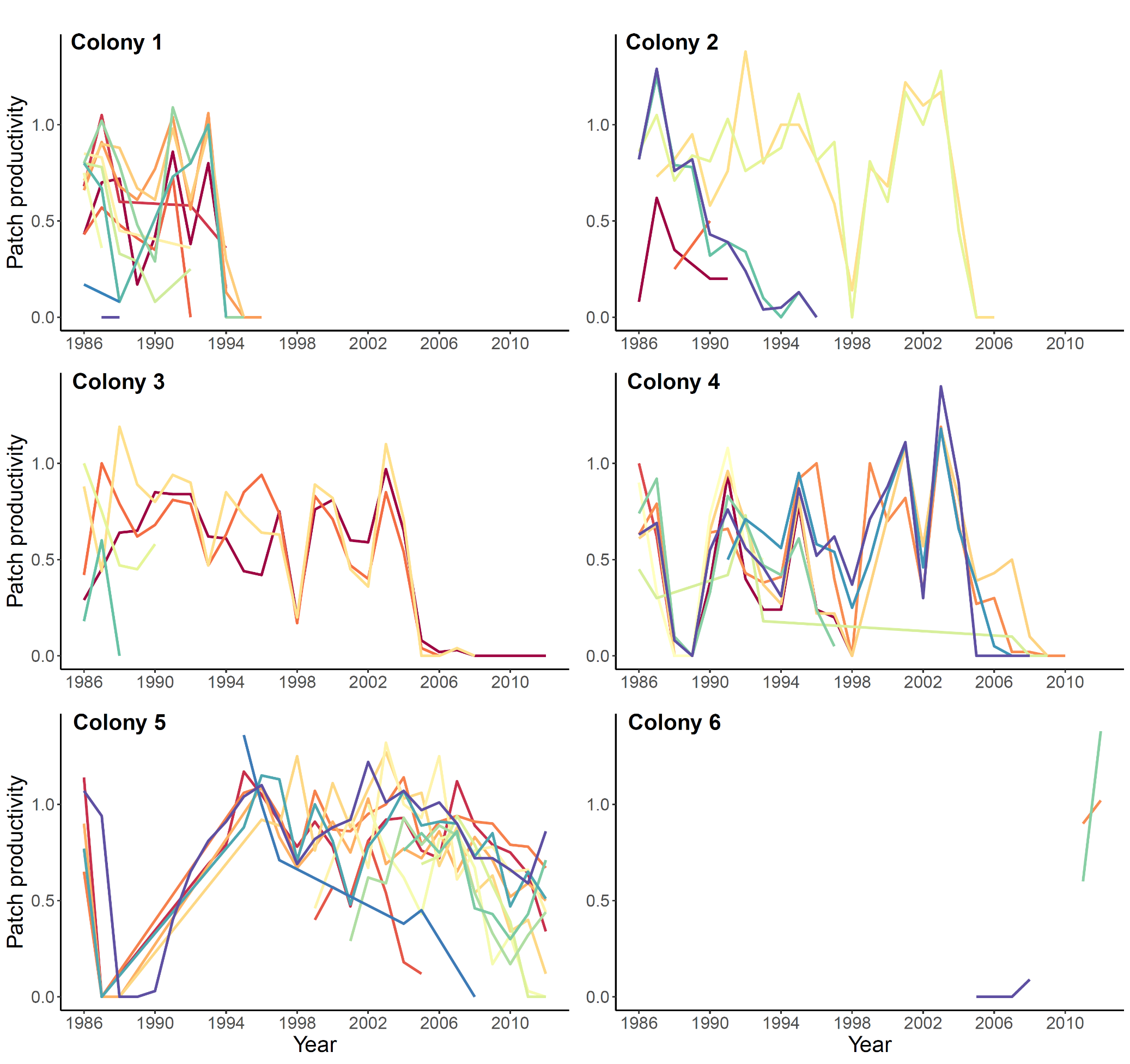


**Figure S3**. Cliff breeding success (divided graphically by colonies) across the 1986-2012 period. Each cliff is represented by a different colour.

This large spatio-temporal heterogeneity in habitat quality has been shown to underly the dynamics of spatial distribution of breeding individuals, via the processes of natal dispersal (for location at recruitment) and breeding dispersal (after recruitment). In our study population, the distribution of individuals is realized by means of habitat selection mechanisms which imply the use of public information in the form of conspecific breeding success, and harsh competition for securing preferred breeding sites (Cadiou et al., 1994; Danchin et al., 1998; Aubry et al., 2009; Bled et al., 2011; Acker et al., 2017). Similar individual-based mechanisms have been shown in another population of kittiwakes, located in the northeast of Norway (Boulinier et al. 2008). Pre-breeders obviously need to acquire a new site to breed, and thus will likely actively contest site dominance in face of other individuals. Further, breeders or skippers may not only defend their previous site against competitors, but they may also disperse (or seek to do so) and thus contest dominance on another breeding site (Danchin et al., 1998; Acker et al., 2017). Notably, an average of ca. 37% of first-time breeders bred in another colony than their natal one), ca. 48% bred in another cove in their natal colony, and ca. 28% bred in another cliff in their natal cove. Further, an average of ca. 26% of individuals that bred in a given year *t* did change breeding site in the next year *t+1* (range 18−43% across 1985−2012). Among these dispersers, ca. 67% (31−84%) changed site within the same cliff, ca. 11% (4−21%) changed cliff within the same cove, ca. 5% (0−10%) changed cove within the same colony, and ca. 17% (4−57%) changed colony. Overall, these breeding dispersal events typically occurred in failed breeders and were more likely to occur from patches of lower quality and towards patches of high quality (Danchin et al. 1998; Bled et al. 2011; Acker et al., 2017).

The intensity of dispersal observed in our study population implies a very high degree of panmixia with no spatial variation in the genetic structure to be expected within the population (McCoy et al. 2005). And indeed, dispersal distances observed within this population are negligible in comparison with daily foraging movements, large-scale prospecting movements, or migratory movements documented in kittiwakes (e.g. McCoy et al. 2005; Bogdanova et al. 2011; Ponchon et al. 2015, 2017). Overall, our study population could hardly be termed a narrow-sense ‘metapopulation’ since it does not meet the criterion of dispersal rarity among metapopulation patches (Hanski & Gilpin 1991, Hanski 1999, Fronhofer et al. 2012). Yet, at all spatial scales within our study population, the spatio-temporal heterogeneity (and geographical discontinuities) that makes the habitat patchy, and connexion between patches through dispersal (which are both pivotal to our working hypotheses and critical features of our study system), are typical attributes that define broad-sense ‘metapopulations’ (e.g. Hanski & Gaggiotti 2004, Ronce 2007). Our study population would even meet the other key criteria of narrow-sense definitions of ‘metapopulation’: dynamics of extinctions and (re)colonizations, and some asynchrony among patches preventing the metapopulation from complete extinction (Fronhofer et al. 2012).

### S1.3 Relationship between spatial heterogeneity and population breeding success

According to prediction (1) formulated in *Introduction*, population habitat quality (represented by population breeding success) should be positively correlated with breeding propensity. A pre-requisite to this prediction (see *Introduction* and *Methods*), is that increased population breeding success reflects increased habitat quality across the population (i.e. increased breeding success over a large proportion of patches), resulting in increased availability of high-quality habitats and hence competition relaxation within the population.

One possible way for measuring heterogeneity in the repartition of a quantitative feature among units is to use the Gini coefficient, which had originally been developed to measure the income inequality within and among countries (Gastwirth, 1972). Many studies in ecology have used the Gini coefficient to measure inequalities among individuals and hence analyse competition processes (e.g. Weiner, 1985; Keeley, 2001; Cordonnier & Kunstler, 2015). This measure of statistical dispersion is one half of the relative mean difference among units under comparison, that is the arithmetic mean of the difference between any two units (calculated across all pairs of units).

Because the number of nests may differ widely among patches (range: 1−261, mean ± standard deviation: 30.10 ± 36.50 among cliffs across years; Acker et al., 2017), we calculated a Gini coefficient weighted by the number of nests in the focal patches (Portnov & Felsenstein, 2010). Indeed, nests are the elementary units from which differences in habitat quality arise, based on which individuals assess patch quality, and for which they compete within a patch.

The weighted Gini coefficient *G_(w)_* is given by the following formula:

$$\text{G}_{\text{(w)}}\text{=}\frac{\sum_{\text{i=1}}^{\text{P}} \sum_{\text{j=1}}^{\text{P}} \text{n}_{\text{i}}\text{n}_{\text{j}}\text{|}\text{r}_{\text{i}}\text{-}\text{r}_{\text{j}}\text{|}}{\text{2}{\bar{\text{r}}}_{\text{(w)}}}$$

where P is the number of patches, *r_i_* is patch breeding success (mean number of fledglings produced per nest) in patch *i*, *n_i_* is the number of nests in patch *i*, and ${\bar{\text{r}}}_{\text{(w)}}$ is the arithmetic mean of patch breeding success weighted by the number of nests in the focal patches.

With this weighted version of the Gini coefficient, between two patches of equal mean breeding success, the patch with the largest number of nests is thus considered to represent a greater proportion of the habitats of high-quality. This is particularly relevant to our hypothesis. Indeed, according to our hypothesis, for the same number of competitors, between two patches of equal breeding success, the patch with more nests should result in greater spatial dilution of competition within the patch (because competitors can spread across more attractive nest sites). In other words, the relaxation of competition occurring when there are more patches of high quality also holds across sub-units within patches (e.g. within cliffs) at lower spatial scales (up to the nest site). Nevertheless, our results were generally robust to such considerations since the non-weighted Gini coefficient did not lead to fundamentally different conclusions.

In the main text, we presented results regarding the inequality in breeding success per nest among cliffs (taken as the patch unit). Visual inspection of the distribution of cliff quality in each year clearly shows that years with low Gini coefficients corresponded to distributions packed around the mean (Fig. S4), i.e. low spatial heterogeneity of habitat quality (which was expected, by definition). Further, high Gini coefficients corresponded to years with a large proportion of cliffs with a very low breeding success (but not the opposite; Fig. S4), i.e. high spatial heterogeneity of habitat quality with a low availability of high-quality habitats. Similar patterns were found at the cove and colony scale (Fig. S5, S6), yet, the colony scale is probably less appropriate to illustrate the importance of spatial heterogeneity in habitat quality, since there were very few colonies every year, and large disparities in colony size in a number of years (Fig. S2).

These distributions translated into a strong negative relationship between the Gini coefficient (measuring the degree of spatial heterogeneity in habitat quality) and mean population habitat quality (Pearson’s correlation coefficient was -0.79; Fig. 1 of main text). Similar results were found when considering patches at larger spatial scales (i.e. coves and colonies). This confirmed our pre-requisite assumption that increases in population breeding success occurs through increases in habitat quality across the population, resulting in increased availability of high-quality sites. Again, similar patterns were found at the cove and colony scale (Pearson’s correlation coefficient was -0.78 and -0.59, respectively; Fig. S7, S8).


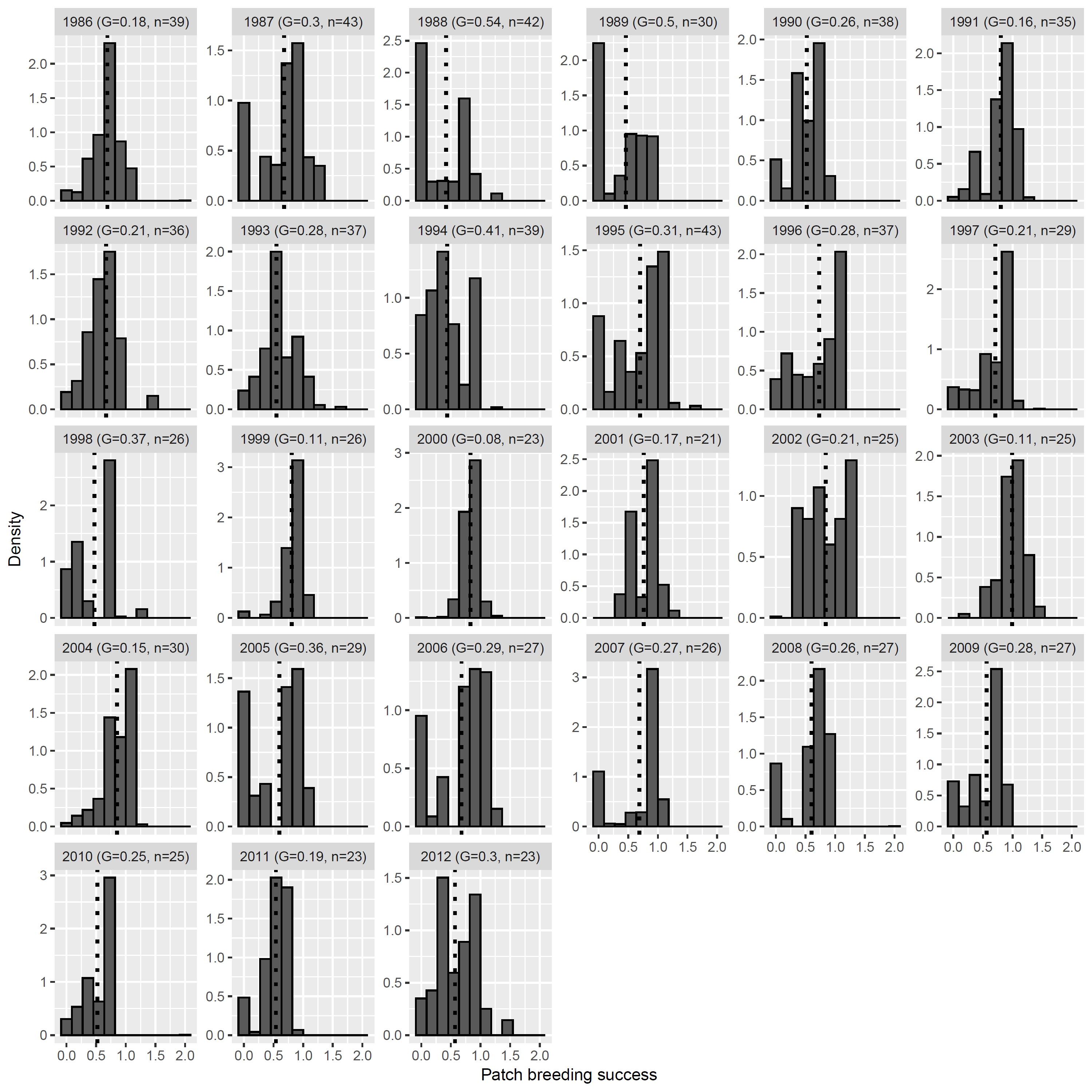


**Figure S4**. Histograms of the distribution of breeding success (number of fledglings produced per nest) among cliffs in each year across the study period. In headings, ‘G’ is the weighted Gini coefficient and ‘n’ the number of cliffs. Dotted line indicates the mean. As for the Gini coefficient, densities are weighted by the number of nests in each cliff. The time series starts in 1986, i.e. the year from which the relationship between mean population habitat quality and breeding propensity was evaluated based on estimates of the IPM.


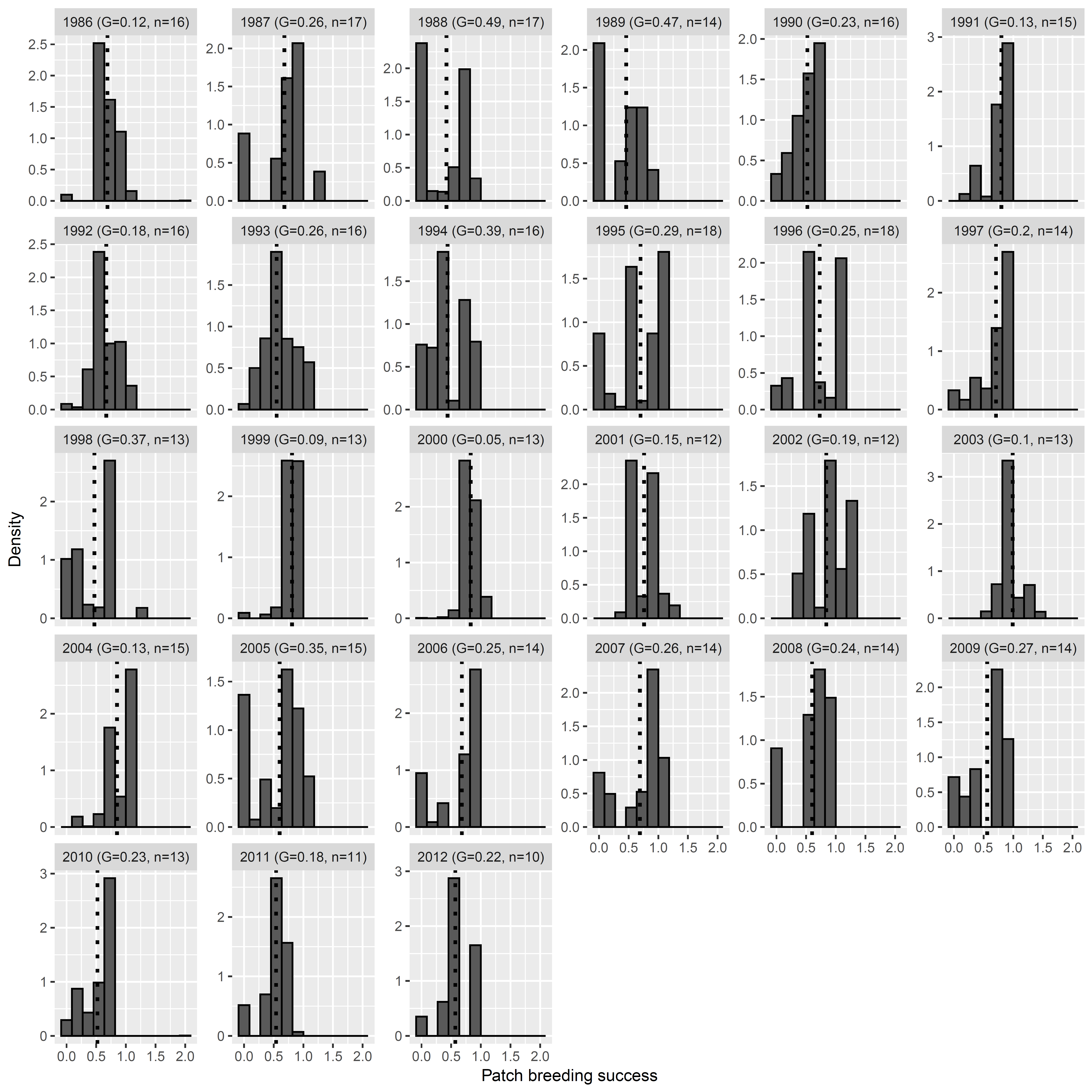


**Figure S5**. Histograms of the distribution of breeding success (number of fledglings produced per nest) among coves in each year across the study period. See Fig. S4 for further specifications.


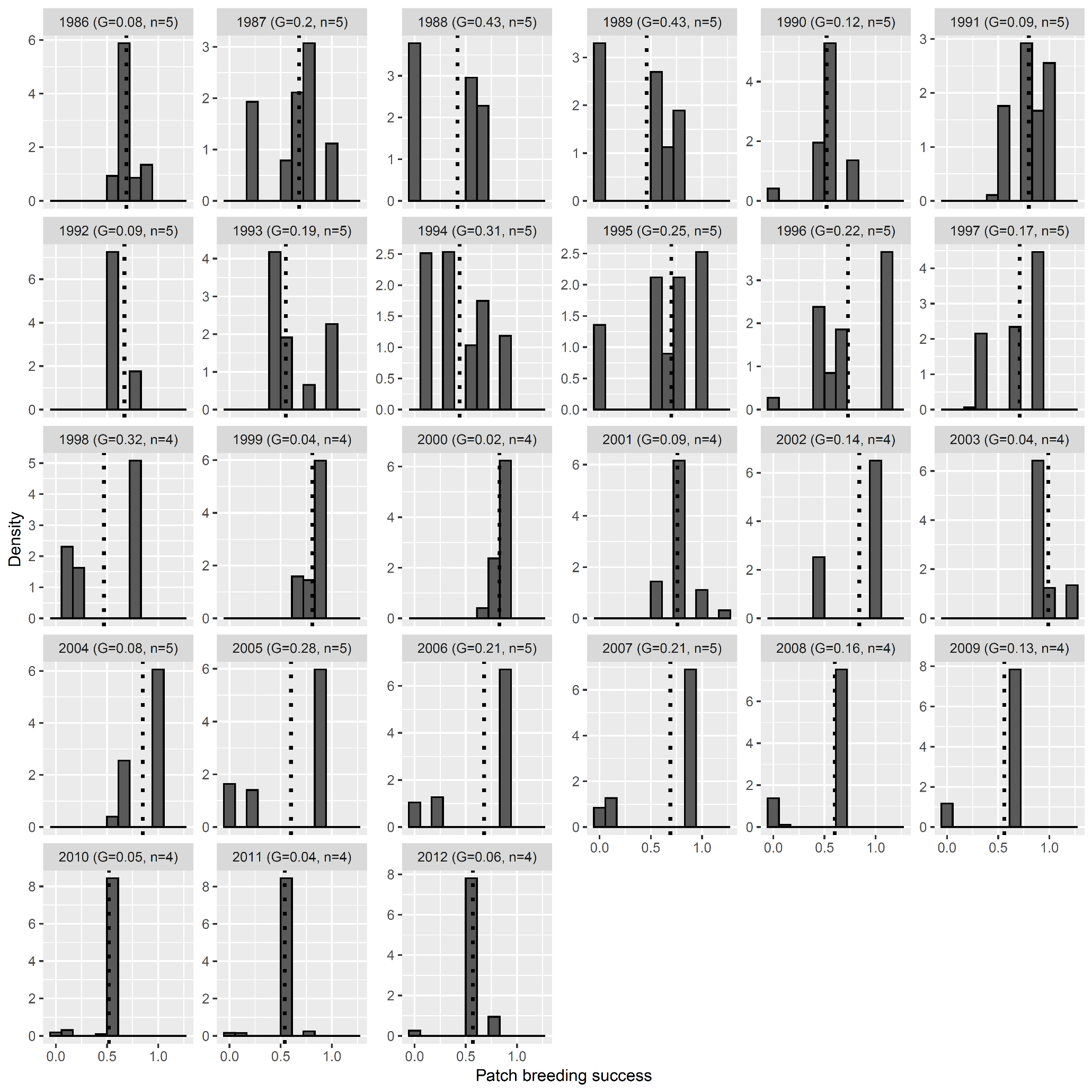


**Figure S6**. Histograms of the distribution of breeding success (number of fledglings produced per nest) among colonies in each year across the study period. See Fig. S4 for further specifications.


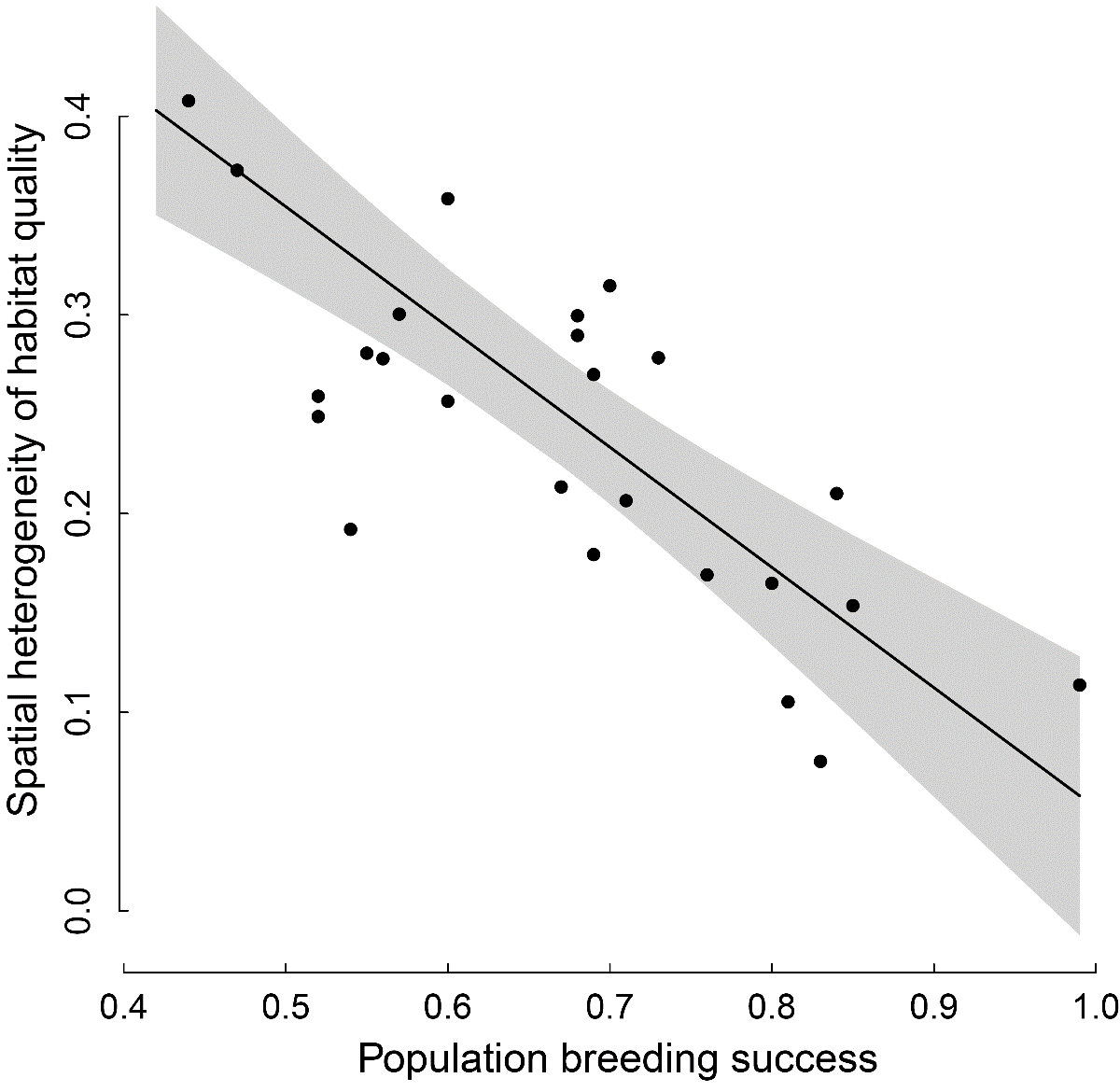


**Figure S7.** Relationship between the degree of spatial heterogeneity of habitat quality (measured by the Gini coefficient at the cove scale) and population breeding success (mean number of fledglings per nest). Grey background: 95% confidence interval of regression line.


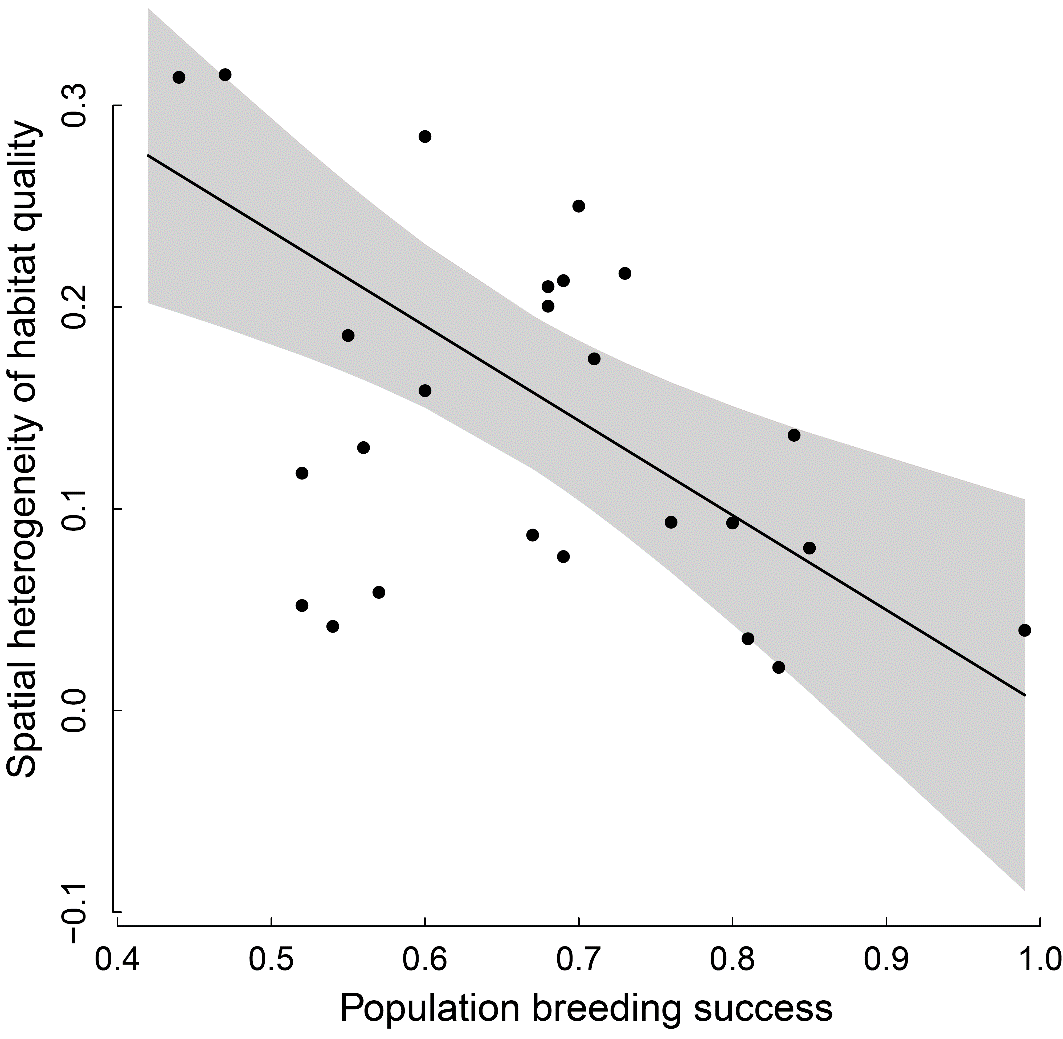


**Figure S8.** Relationship between the degree of spatial heterogeneity of habitat quality (measured by the Gini coefficient at the cliff scale) and population breeding success (mean number of fledglings per nest). See Fig. S7 for further specifications.

### S1.4 References

Acker, P., Besnard, A., Monnat, J.-Y., & Cam, E. (2017). Breeding habitat selection across spatial scales: is grass always greener on the other side? *Ecology*, *98*(10), 2684–2697. <https://doi.org/10.1002/ecy.1962>

Aubry, L. M., Cam, E., & Monnat, J.-Y. (2009). Habitat selection, age-specific recruitment and reproductive success in a long-lived Seabird. In D. L. Thomson, E. G. Cooch, & M. J. Conroy (Eds.), *Modeling Demographic Processes In Marked Populations* (pp. 365–392). New York, NY: Springer. <https://doi.org/10.1007/978-0-387-78151-8_16>

Bled, F., Royle, J. A., & Cam, E. (2011). Assessing hypotheses about nesting site occupancy dynamics. *Ecology, 92(4)*, 938-951. <https://doi.org/10.1890/10-0392.1>

Bogdanova, M. I., Daunt, F., Newell, M., Phillips, R. A., Harris, M. P., & Wanless, S. (2011). Seasonal interactions in the black-legged kittiwake, Rissa tridactyla: Links between breeding performance and winter distribution. Proceedings of the Royal Society B: Biological Sciences, 278(1717), 2412–2418. <https://doi.org/10.1098/rspb.2010.2601>

Boulinier, T., McCoy, K. D., Yoccoz, N. G., Gasparini, J., & Tveraa, T. (2008). Public information affects breeding dispersal in a colonial bird: Kittiwakes cue on neighbours. Biology Letters, 4(5), 538–540. <https://doi.org/10.1098/rsbl.2008.0291>

Cadiou, B., Monnat, J. Y., & Danchin, E. (1994). Prospecting in the kittiwake, *Rissa tridactyla*: different behavioural patterns and the role of squatting in recruitment. *Animal Behaviour*, *47(4)*, 847–856. <https://doi.org/10.1006/anbe.1994.1116>

Cadiou, B. 1993. *L’accession à la reproduction: un processus social d’ontogenèse*. PhD Dissertation, Université de Rennes 1, France.

Cordonnier, T., & Kunstler, G. (2015). The Gini index brings asymmetric competition to light. Perspectives in Plant Ecology, Evolution and Systematics, 17(2), 107-115. <https://doi.org/10.1016/j.ppees.2015.01.001>

Danchin, Etienne, Boulinier, T., & Massot, M. (1998). Conspecific reproductive success and breeding habitat selection: implications for the study of coloniality. *Ecology*, *79*(7), 2415–2428. <https://doi.org/10.2307/176832>

Fronhofer, E. A., Kubisch, A., Hilker, F. M., Hovestadt, T., & Poethke, H. J. (2012). Why are metapopulations so rare? Ecology, 93(8), 1967–1978. <https://doi.org/10.1890/11-1814.1>

Gastwirth, J. L. (1972). The estimation of the Lorenz curve and Gini index. The review of economics and statistics, 306-316. <https://doi.org/10.2307/1937992>

Guermeur, Y., & Monnat, J.-Y. (1980). Histoire et géographie des oiseaux nicheurs de Bretagne. Ministère de l'environnement et du cadre de vie, Direction de la protection de la nature.

Hanski, I., & Gilpin, M. (1991). Metapopulation dynamics: Brief history and conceptual domain. Biological Journal of the Linnean Society, 42(1–2), 3–16. <https://doi.org/10.1111/j.1095-8312.1991.tb00548.x>

Hanski, I. (1999). Habitat connectivity, habitat continuity, and metapopulations in dynamic landscapes. Oikos, 209–219. <https://doi.org/10.2307/3546736>

Hanski, I. A., & Gaggiotti, O. E. (2004). Ecology, genetics and evolution of metapopulations. Academic Press.

Keeley, E. R. (2001). Demographic responses to food and space competition by juvenile steelhead trout. *Ecology, 82*(5), 1247-1259. [https://doi.org/10.1890/0012-9658(2001)082[1247:DRTFAS]2.0.CO;2](https://doi.org/10.1890/0012-9658(2001)082%5b1247:DRTFAS%5d2.0.CO;2)

McCoy, K. D., Boulinier, T., & Tirard, C. (2005). Comparative host–parasite population structures: Disentangling prospecting and dispersal in the black-legged kittiwake Rissa tridactyla. Molecular Ecology, 14(9), 2825–2838. <https://doi.org/10.1111/j.1365-294X.2005.02631.x>

Monnat, J.-Y., & Cadiou, B. 2004. Mouette tridactyle. Pages 140-147 *in* B. Cadiou, J. Pons, and P. Yésou, editors. Oiseaux marins nicheurs de France métropolitaine. Biotope, Mèze, France.

Ponchon, A., Chambert, T., Lobato, E., Tveraa, T., Grémillet, D., & Boulinier, T. (2015). Breeding failure induces large scale prospecting movements in the black-legged kittiwake. Journal of Experimental Marine Biology and Ecology, 473, 138–145. <https://doi.org/10.1016/j.jembe.2015.08.013>

Ponchon, A., Iliszko, L., Grémillet, D., Tveraa, T., & Boulinier, T. (2017). Intense prospecting movements of failed breeders nesting in an unsuccessful breeding subcolony. Animal Behaviour, 124, 183–191. <https://doi.org/10.1016/j.anbehav.2016.12.017>

Portnov, B. A., & Felsenstein, D. (2010). On the suitability of income inequality measures for regional analysis: Some evidence from simulation analysis and bootstrapping tests. Socio-Economic Planning Sciences, 44(4), 212-219. <https://doi.org/10.1016/j.seps.2010.04.002>.

Ronce, O. (2007). How Does It Feel to Be Like a Rolling Stone? Ten Questions About Dispersal Evolution. Annual Review of Ecology, Evolution, and Systematics, 38(1), 231–253. <https://doi.org/10.1146/annurev.ecolsys.38.091206.095611>

Weiner, J. (1985). Size hierarchies in experimental populations of annual plants. Ecology, 66(3), 743-752. <https://doi.org/10.2307/1940535>

## APPENDIX S2

## Details of the integrated population model

This appendix provides additional details to the description of the integrated population model (see *Materials and Methods*) and the BUGS code to fit this model in JAGS.

**Contents:**

**S2.1 Population matrix and projection equation (p. 18)**

**S2.2 On the assumption of equal survival in immigrants and natives (p. 20)**

**S2.3 Likelihood for the count data (p. 22)**

**S2.4 Likelihood for the capture-recapture data (p. 23)**

**S2.5 Likelihood for the reproduction data (p. 24)**

**S2.6 Prior distributions (p. 26)**

**S2.7 BUGS code (p. 27)**

**S2.8 References (p. 34)**

### S2.1 Population matrix and projection equation

The equation below describes changes in the vector of population sizes as a function of the population matrix (deterministic version). The vector of population sizes contains the number of individuals *N_X_* in each life-history state *X* (see *Materials and Methods*, Fig. 1). The number of immigrants *N_I_* is added up to the number of local first-time breeders generated locally to lead to the number of first-time breeders *N_F_* in the population (see *Materials and Methods*, Fig. 2). All numbers and demographic rates are year-dependent.

$$\left[ \begin{matrix} \text{N}_{\text{Y}} \\ \text{N}_{\text{P2}} \\ \text{N}_{\text{P3}} \\ \text{N}_{\text{P4}} \\ \text{N}_{\text{P5}} \\ \text{N}_{\text{P6}} \\ \text{N}_{\text{F}} \\ \text{N}_{\text{E}} \\ \text{N}_{\text{S}} \end{matrix} \right]_{\text{t}}\text{=}\left[ \begin{matrix} \text{0} & \text{0} & \text{0} & \text{0} & \text{0} & \text{0} & \text{ϕ}_{\text{0}}\text{π}_{\text{f}} & \text{ϕ}_{\text{0}}\text{π}_{\text{e}} & \text{0} \\ \text{ϕ}_{\text{0}} & \text{0} & \text{0} & \text{0} & \text{0} & \text{0} & \text{0} & \text{0} & \text{0} \\ \text{0} & \text{ϕ}_{\text{2}}\text{(1-}\text{ρ}_{\text{3}}\text{)} & \text{0} & \text{0} & \text{0} & \text{0} & \text{0} & \text{0} & \text{0} \\ \text{0} & \text{0} & \text{ϕ}_{\text{2}}\text{(1-}\text{ρ}_{\text{4}}\text{)} & \text{0} & \text{0} & \text{0} & \text{0} & \text{0} & \text{0} \\ \text{0} & \text{0} & \text{0} & \text{ϕ}_{\text{2}}\text{(1-}\text{ρ}_{\text{5}}\text{)} & \text{0} & \text{0} & \text{0} & \text{0} & \text{0} \\ \text{0} & \text{0} & \text{0} & \text{0} & \text{ϕ}_{\text{2}}\text{(1-}\text{ρ}_{\text{6}}\text{)} & \text{0} & \text{0} & \text{0} & \text{0} \\ \text{0} & \text{ϕ}_{\text{2}}\text{ρ}_{\text{3}} & \text{ϕ}_{\text{2}}\text{ρ}_{\text{4}} & \text{ϕ}_{\text{2}}\text{ρ}_{\text{5}} & \text{ϕ}_{\text{2}}\text{ρ}_{\text{6}} & \text{ϕ}_{\text{2}} & \text{0} & \text{0} & \text{0} \\ \text{0} & \text{0} & \text{0} & \text{0} & \text{0} & \text{0} & \text{ϕ}_{\text{2}}\text{ψ}_{\text{b}} & \text{ϕ}_{\text{2}}\text{ψ}_{\text{b}} & \text{ϕ}_{\text{2}}\text{ψ}_{\text{s}} \\ \text{0} & \text{0} & \text{0} & \text{0} & \text{0} & \text{0} & \text{ϕ}_{\text{2}}\text{(1-}\text{ψ}_{\text{b}}\text{)} & \text{ϕ}_{\text{2}}\text{(1-}\text{ψ}_{\text{b}}\text{)} & \text{ϕ}_{\text{2}}\text{(1-}\text{ψ}_{\text{s}}\text{)} \end{matrix} \right]_{\text{t-1}}\left[ \begin{matrix} \text{N}_{\text{Y}} \\ \text{N}_{\text{P2}} \\ \text{N}_{\text{P3}} \\ \text{N}_{\text{P4}} \\ \text{N}_{\text{P5}} \\ \text{N}_{\text{P6}} \\ \text{N}_{\text{F}} \\ \text{N}_{\text{E}} \\ \text{N}_{\text{S}} \end{matrix} \right]_{\text{t-1}}\text{+}\left[ \begin{matrix} \text{0} \\ \text{0} \\ \text{0} \\ \text{0} \\ \text{0} \\ \text{0} \\ \text{N}_{\text{I}} \\ \text{0} \\ 0 \end{matrix} \right]_{\text{t}}$$

where *Y* is for yearlings, *Pi* is for prebreeders of age *i* (2 ≤ *i* ≤ 6), *F* is for first-time breeders, *E* is for experienced breeders, *S* is for skippers, *I* is for immigrants; *t* is the year; $\text{π}_{\text{f}}$ is the *per capita* breeding success of first-time breeders, $\text{π}_{\text{e}}$ is the *per capita* breeding success of experienced breeders, $\text{ϕ}_{\text{0}}$ is the annual survival rate at age 0 and age 1, $\text{ϕ}_{\text{2}}$ is the annual survival from age 2, $\text{ρ}_{\text{3}}$ is the recruitment rate at age 3, ..., $\text{ρ}_{\text{6}}$ is the recruitment rate at age 6 (recruitment rate at age 7 is 1), $\text{ψ}_{\text{b}}$ is the breeding rate of former breeders, $\text{ψ}_{\text{s}}$ is the breeding rate of former skippers.

As specified in the main text, we assumed no recruitment before age 3 and after age 7. However, a few individuals have been recorded breeding for the first time at age 2 (ca. 0.5‰) or between age 8 and 14 (ca. 4‰). We ignored these cases by considering the first ones (who bred at age 2) as still nonbreeders at age 2 and breeding for the first time at their next reproductive attempt, and by discarding the entire capture-recapture histories of the second ones (who bred for the first time after age 7).

This deterministic version of the matrix population model was extended to include demographic stochasticity. Demographic stochasticity was considered by using binomial and Poisson distributions to describe the link between state-specific numbers in year *t*+1 and *t*. Environmental stochasticity is represented by year-dependence in demographic rates and state-specific numbers (index *t* for year). We specified the following relationships:

$$\text{N}_{\text{Y,t}\text{+1}}\text{ \textasciitilde}\text{Poisson}\text{ (}\text{ϕ}_{\text{0,t}}{\text{ }\text{π}}_{\text{f,t}}\text{ N}_{\text{F,t}}\text{ +}{\text{ }\text{ϕ}}_{\text{0,t }}\text{π}_{\text{e,t}}\text{ N}_{\text{E,t}}\text{)}$$

$$\text{N}_{\text{P2,t}\text{+1}}\text{ \textasciitilde}\text{Binomial}\text{ (}\text{ϕ}_{\text{0,t}}\text{ , }\text{Y}_{\text{t}}\text{)}$$

$$\text{N}_{\text{P3,t}\text{+1}}\text{ \textasciitilde}\text{Binomial}\text{ (}\text{ϕ}_{\text{2,t}}\text{ (1-}\text{ρ}_{\text{3,t}}\text{), }\text{N}_{\text{P2,t}}\text{)}$$

$$\text{N}_{\text{P4,t}\text{+1}}\text{ \textasciitilde}\text{Binomial}\text{ (}\text{ϕ}_{\text{2,t}}\text{ (1-}\text{ρ}_{\text{4,t}}\text{), }\text{N}_{\text{P3,t}}\text{)}$$

$$\text{N}_{\text{P5,t}\text{+1}}\text{ \textasciitilde}\text{Binomial}\text{ (}\text{ϕ}_{\text{2,t}}\text{ (1-}\text{ρ}_{\text{5,t}}\text{), }\text{N}_{\text{P4,t}}\text{)}$$

$$\text{N}_{\text{P6,t}\text{+1}}\text{ \textasciitilde}\text{Binomial}\text{ (}\text{ϕ}_{\text{2,t}}\text{ (1-}\text{ρ}_{\text{6,t}}\text{), }\text{N}_{\text{P5,t}}\text{)}$$

$\text{N}_{\text{F,t}\text{+1}}\text{ \textasciitilde}\sum_{\text{i}\text{=3}}^{\text{6}} \left( \text{Binomial} (\text{ϕ}_{\text{2,t}}\text{ }\text{ρ}_{\text{i,t}} \text{,} \text{N}_{\text{P(i}\text{-1}\text{),t}}\text{)} \right)\text{+ }\text{Binomial}\text{ (}\text{ϕ}_{\text{2,t}} \text{, }\text{N}_{\text{P6,t}}\text{) +}{\text{ }\text{N}}_{\text{I,t+1}}\text{ }$

$$\text{N}_{\text{E,t}\text{+1}}\text{ \textasciitilde}\text{Binomial}\text{ (}\text{ϕ}_{\text{2,t}}\text{ }\text{ψ}_{\text{b,t}}\text{ , }\text{N}_{\text{F,t}}\text{) + }\text{Binomial}\text{ (}\text{ϕ}_{\text{2,t}}\text{ }\text{ψ}_{\text{b,t}}\text{ , }\text{N}_{\text{E,t}}\text{) + }\text{Binomial}\text{ (}\text{ϕ}_{\text{2,t}}\text{ }\text{ψ}_{\text{s,t}}\text{ , }\text{N}_{\text{S,t}}\text{)}$$

$$\text{N}_{\text{S,t}\text{+1}}\text{ \textasciitilde}\text{Binomial}\text{ (}\text{ϕ}_{\text{2,t}}{\text{(1-}\text{ψ}}_{\text{b,t}}\text{), }\text{N}_{\text{F,t}}\text{) + }\text{Binomial}\text{ (}\text{ϕ}_{\text{2,t}}{\text{(1-}\text{ψ}}_{\text{b,t}}\text{)}\text{, }\text{N}_{\text{E,t}}\text{)}\text{ }\text{+ }\text{Binomial}\text{ (}\text{ϕ}_{\text{2,t}}{\text{(1-}\text{ψ}}_{\text{s,t}}\text{)}\text{, }\text{N}_{\text{S,t}}\text{)}$$

and $\text{N}_{\text{I,t+1}}\text{ }\text{\textasciitilde}\text{ Uniform }\text{(-5,}$1000)

### S2.2 On the assumption of equal survival in immigrants and natives

Recall that our population integrated model relies on the assumption of equal apparent survival between natives and immigrants once established in the population. This assumption is necessary if immigrants are not individually monitored. Indeed, because immigrants are not marked, they cannot be distinguished from locals. If this assumption does not hold, the estimated number of immigrants would be negatively biased in the case of a lower survival of the immigrants than the natives, while it would be positively biased in the case of a higher survival in natives than immigrants. One may thus regard our estimated numbers of immigrants as *effective immigration sizes,* i.e. numbers of individuals identical to natives necessary to yield the observed dynamics.

Several processes might lead to a survival difference between natives and immigrants, but we currently have no evidence to specifically support one hypothesis or another. We are lacking empirical and theoretical bases to move forwards in the discussion of survival differences between natives and immigrants in kittiwakes. This lack of evidence is due to the current impossibility to mark all individuals in our population, or to monitor immigration in such a highly mobile species with a large geographic range. In the future, electronic devices for individual monitoring might help answer this question (see e.g. Ponchon et al., 2013).

Immigrants might be inferior survivors if they pay a cost of long-distance transfer, lack of familiarity, or maladaptation (Baker & Rao, 2004; Burgess et al., 2012; Bonte et al., 2012). Conversely, such dispersal costs could prevent frailer individuals from achieving immigration, which would result in higher survival in immigrants than natives. However, kittiwakes are highly mobile (they winter thousands of kilometers from breeding colonies; Frederiksen et al. 2012); it makes little sense that they would pay direct survival costs of moving between breeding areas. Further, large effective immigration (as found in our study, see *Results*) should prevent local adaptation in natives (Lenormand, 2002). This tends to make the hypothesis of a difference in survival probability due to a survival cost in immigrants relatively fragile.

We could also argue that immigrants might express a dispersal syndrome (Clobert et al., 2012), and therefore would have a higher probability of emigrating permanently than natives. However, heritable components of dispersal propensity would be transmitted to natives by the large effective number of immigrants (Phillips et al., 2008): natives might also exhibit a high dispersal probability. This would be in line with the hypothesis that the low survival in the Cap Sizun population might stem from a high level of permanent emigration. Finally, natives might be attracted to more favorable habitats outside the study area while immigrants have been attracted to the study area, which might also translate into lower local survival in natives. Nonetheless, it would be hard to conceive that immigrants once attending the population would not be attracted to the same habitats outside the study area as do locals.

Based on these considerations, major survival differences between immigrant and locals do not seem especially likely. Moreover, the few existing estimations in four other species of colonial seabirds have not shown any strong survival differences in immigrants and locals (Oro et al., 2011; Barbraud & Delord, 2021). These studies reported estimations from samples pooled across years only, showing only (very) small differences in survival probability, with weak statistical support, if any. Such small differences do not suggest that inferences from IPMs would yield flawed conclusions, given typical imprecision in demographic parameters and resulting immigration estimates. Overall, the currently required IPM assumption of equal survival in immigrants and native thus appears as a realistic one.

### S2.3 Likelihood for the count data

The state-space model is composed of a state process model describing the true fluctuations of the stage-specific population sizes across years, and an observation model describing the link between the true and the observed size of the population (de Valpine & Hastings, 2002). The state process model is described above by the matrix population model, as well as an initial state vector (i.e. vector of population sizes at year *t=*1 in 1985, see priors and BUGS code below). For the observation model we assumed that the observation error was normally distributed on the log scale and constant across years. Since only individuals that are actually breeding in a focal year *t* are counted, the count data *C_t_* were modeled as follows:

$\log\left( \text{C}_{\text{t}} \right)\text{ \textasciitilde}\text{Normal }\left( \log\left( \text{N}_{\text{F,t}}\text{ +}\text{ N}_{\text{E,t}}+\text{ N}_{\text{I,t}} \right)\text{, }\text{σ}_{\text{obs}}^{\text{2}} \right)$.

The likelihood for the complete state-space model was composed of the likelihood for the state-process and the observation process (Kéry & Schaub, 2012).

### S2.4 Likelihood for the capture-recapture data

To estimate survival, recruitment, and breeding rates from the capture-resighting histories, we used a multistate capture-recapture model (Lebreton et al., 2009) with a state-space formulation (Gimenez et al., 2007, Kéry & Schaub, 2012). The state process is readily deductible from the life cycle without immigration (Fig. 2, population matrix above). More precisely, we considered a matrix *Z* with elements *z_i,t_*, indicating the true state of individual *i* at year *t* (*z_i,t_* $\text{∈}$ {1, …, 10}). The ten states were ‘fledgling’, ‘yearling’, ‘prebreeder of age 2’, ..., ‘prebreeder of age 6’, ‘breeder’, ‘skipper’ and ‘dead’. We assumed no error in state assignment. Given the state and year at first observation of the focal individual (which is always known here), we modeled the succession of states across years with a categorical distribution:

$\text{z}_{\text{i,t+1}}\text{|}\text{z}_{\text{i,t}}\text{ }\text{\textasciitilde}\text{Categorical}\text{ }\left( \text{Ω}_{\text{t}\text{,}\text{1}\text{…}\text{10}\text{,}\text{t}} \right)$ .

where Ω is the state-transition matrix, defined as follows (see above for description of the parameters):

$\text{Ω}_{\text{t}}\text{=}\left[ \begin{matrix} \text{0} & \text{ϕ}_{\text{0}} & \text{0} & \text{0} & \text{0} & \text{0} & \text{0} & \text{0} & \text{0} & \text{1-}\text{ϕ}_{\text{0}} \\ \text{0} & \text{0} & \text{ϕ}_{\text{1}} & \text{0} & \text{0} & \text{0} & \text{0} & \text{0} & \text{0} & \text{1-}\text{ϕ}_{\text{0}} \\ \text{0} & \text{0} & \text{0} & \text{ϕ}_{\text{2}}\text{(1-}\text{ρ}_{\text{3}}\text{)} & \text{0} & \text{0} & \text{0} & \text{ϕ}_{\text{2}}\text{ρ}_{\text{3}} & \text{0} & \text{1-}\text{ϕ}_{\text{2}} \\ \text{0} & \text{0} & \text{0} & \text{0} & \text{ϕ}_{\text{2}}\text{(1-}\text{ρ}_{\text{4}}\text{)} & \text{0} & \text{0} & \text{ϕ}_{\text{2}}\text{ρ}_{\text{4}} & \text{0} & \text{1-}\text{ϕ}_{\text{2}} \\ \text{0} & \text{0} & \text{0} & \text{0} & \text{0} & \text{ϕ}_{\text{2}}\text{(1-}\text{ρ}_{\text{5}}\text{)} & \text{0} & \text{ϕ}_{\text{2}}\text{ρ}_{\text{3}} & \text{0} & \text{1-}\text{ϕ}_{\text{2}} \\ \text{0} & \text{0} & \text{0} & \text{0} & \text{0} & \text{0} & \text{ϕ}_{\text{2}}\text{(1-}\text{ρ}_{\text{6}}\text{)} & \text{ϕ}_{\text{2}}\text{ρ}_{\text{6}} & \text{0} & \text{1-}\text{ϕ}_{\text{2}} \\ \text{0} & \text{0} & \text{0} & \text{0} & \text{0} & \text{0} & \text{0} & \text{ϕ}_{\text{2}} & \text{0} & \text{1-}\text{ϕ}_{\text{2}} \\ \text{0} & \text{0} & \text{0} & \text{0} & \text{0} & \text{0} & \text{0} & \text{ϕ}_{\text{2}}\text{ψ}_{\text{b}} & \text{ϕ}_{\text{2}}\text{ψ}_{\text{b}} & \text{1-}\text{ϕ}_{\text{2}} \\ \text{0} & \text{0} & \text{0} & \text{0} & \text{0} & \text{0} & \text{0} & \text{ϕ}_{\text{2}}\text{ψ}_{\text{s}} & \text{ϕ}_{\text{2}}\text{(1-}\text{ψ}_{\text{s}}\text{)} & \text{1-}\text{ϕ}_{\text{2}} \\ \text{0} & \text{0} & \text{0} & \text{0} & \text{0} & \text{0} & \text{0} & \text{0} & \text{0} & \text{1} \end{matrix} \right]_{t}$.

There were six observation events: ‘seen as fledgling’ (i.e. at ringing), ‘seen as prebreeder’, ‘seen as first-time breeder’, ‘seen as experienced breeder’, ‘seen as skipper’, and ‘not seen’. The observations were provided in the matrix *Ο* containing the capture-resighting histories (each element *o_i,t_* is the observation event concerning the individual *i* at year *t*). The observation process links the true states with the observation events. Given the true state, we modeled the sequences of observations events with a categorical distribution, from the year after the year of first observation of the focal individual:

$\text{o}_{\text{i,t}}\text{|}\text{z}_{\text{i,t}}\text{ \textasciitilde}\text{Categorical} \left( \text{Θ}_{\text{t}\text{,}\text{1}\text{…}\text{6}\text{,}\text{t}} \right)$ .

where Θ is the observation matrix, defined as follows:

$$\text{Θ}_{\text{t}}\text{=}\left[ \begin{matrix} \text{1} & \text{0} & 0 & \text{0} & \text{0} & \text{0} \\ 0 & \text{p}_{\text{y}} & 0 & \text{0} & \text{0} & \text{1-}\text{p}_{\text{y}} \\ 0 & \text{0} & \text{p}_{\text{p}} & \text{0} & \text{0} & \text{1-}\text{p}_{\text{p}} \\ 0 & \text{0} & \text{p}_{\text{p}} & \text{0} & \text{0} & \text{1-}\text{p}_{\text{p}} \\ 0 & \text{0} & \text{p}_{\text{p}} & \text{0} & \text{0} & \text{1-}\text{p}_{\text{p}} \\ 0 & \text{0} & \text{p}_{\text{p}} & \text{0} & \text{0} & \text{1-}\text{p}_{\text{p}} \\ 0 & \text{0} & \text{p}_{\text{p}} & \text{0} & \text{0} & \text{1-}\text{p}_{\text{p}} \\ \text{0} & \text{p}_{\text{bs}} & 0 & \text{p}_{\text{bs}} & \text{0} & \text{1-}\text{p}_{\text{bs}} \\ 0 & \text{0} & 0 & \text{0} & \text{p}_{\text{bs}} & \text{1-}\text{p}_{\text{bs}} \\ \text{0} & \text{0} & 0 & \text{0} & \text{0} & \text{1} \end{matrix} \right]_{\text{t}}$$

where *p_y,t_* was the resighting probability of yearlings in year *t*, *p_p,t_* was the resighting probability of prebreeders of age 2 to 6 in year *t*, and *p_bs,t_* was the reencounter probability of breeders and skippers in year *t*.

All the demographic parameters and resighting probabilities were modeled with random year effects. Thus, any parameter *θ_t_* was modeled as follows:

$$\text{logit} \left( \text{θ}_{\text{t}} \right)\text{ \textasciitilde}\text{Normal} \left( \bar{\text{θ}}\text{ ,} \text{σ}_{\text{θ}}^{\text{2}} \right)$$

where $\bar{\theta}$ is the mean parameter across years on the logit scale, and $\text{σ}_{\text{θ}}^{\text{2}}$ is the temporal variance of the parameter. The use of random year effects results in year-specific parameters that are shrunk towards the mean parameter (Burnham & White, 2002). The degree of shrinkage increases when precision decreases, which is a desired property (Kéry & Schaub, 2012).

### S2.5 Likelihood for the reproduction data

We used three Poisson regressions to estimate *per capita* breeding success of breeders from status-specific counts of fledglings per nest. The first regression refers to the breeding success of breeding pairs composed of two first-time breeders. In each year *t*, *J_F,t_* is the total count of fledglings produced by this category of breeding pairs, and *R_F,t_* is the count of breeding pairs that raised these fledglings. For this category, breeding success was thus modelled as:

$\text{J}_{\text{F,t}}\text{ \textasciitilde}\text{Poisson}\text{ (}\text{R}_{\text{F,t }}{\text{2}\text{π}}_{\text{f}}\text{)}$ .

The second regression refers to the breeding success of pairs composed of two experienced breeders. In each year *t*, *J_E,t_* is the total count of fledglings produced by this category of breeding pairs, and *R_E,t_* is the count of breeding pairs that raised these fledglings. For this category, the breeding success was thus modelled as:

$\text{J}_{\text{E,t}}\text{ \textasciitilde}\text{Poisson}\text{ (}\text{R}_{\text{E,t }}{\text{2}\text{π}}_{e}\text{)}$ .

The third regression refers to the breeding success of pairs for which the two individuals were of different experience status, or at least one individual was of unknown status. In each year *t*, *J_U,t_* is the total count of fledglings produced by this category of breeding pairs, and *R_U,t_* is the count of breeding pairs that raised these fledglings. Because we used *per capita* breeding success rates and ignored pair characteristics, we assumed that the breeding success rate was an average of breeding success of inexperienced and experienced breeders weighted by their respective proportion among breeders in the model. For this category of breeding pairs, the breeding success was thus modelled as:

$\text{J}_{\text{U,t}}\text{ \textasciitilde}\text{Poisson}\text{ (}\text{R}_{\text{U,t }}{\text{2(}\text{π}}_{\text{f,t}}\frac{\text{N}_{\text{F,t}}}{\text{N}_{\text{F,t}}\text{+}\text{N}_{\text{E,t}}}\text{+}\text{π}_{\text{e,t}}\frac{\text{N}_{\text{E,t}}}{\text{N}_{\text{F,t}}\text{+}\text{N}_{\text{E,t}}}\text{))}$ .

We modeled breeding success with random year effects, thus we have:

$$\text{log} \left( \text{π}_{\text{f,t}} \right)\text{ \textasciitilde}\text{Normal} \left( \bar{\text{π}_{\text{f}}}\text{ ,} \text{σ}_{\text{π}_{\text{f}}}^{\text{2}} \right)$$

$$\text{log}\text{ }\left( \text{π}_{\text{e,t}} \right)\text{ \textasciitilde}\text{Normal}\text{ }\left( \bar{\text{π}_{\text{e}}}\text{ ,}\text{ }\text{σ}_{\text{π}_{\text{e}}}^{\text{2}} \right)$$

where $\bar{\text{π}_{\text{f}}}$ is the mean breeding success of first-time breeders across years on the log scale, and $\text{σ}_{\text{π}_{\text{f}}}^{\text{2}}$ is the temporal variance of this parameter; $\bar{\text{π}_{\text{e}}}$ is the mean breeding success of experienced breeders across years on the log scale, and $\text{σ}_{\text{π}_{\text{e}}}^{\text{2}}$ is the temporal variance of this parameter.

### S2.6 Prior distributions

The prior distributions for each parameter are the following:

- Mean survival: $\bar{\text{ϕ}_{\text{0}}}\text{ \textasciitilde}$ *Uniform* (0,1); $\bar{\text{ϕ}_{\text{2}}}\text{ \textasciitilde}$ *Uniform* (0,1).
- Temporal variability of survival (priors on the standard deviation, on the logit scale):

$\text{σ}_{\text{ϕ}_{\text{0}}}\text{ \textasciitilde}$ *Uniform* (0,10); $\text{σ}_{\text{ϕ}_{\text{2}}}\text{\textasciitilde}$ *Uniform* (0,10).

- Mean probability of recruitment: $\bar{\text{ρ}_{\text{3}}}\text{ \textasciitilde}$ *Uniform* (0,1); $\bar{\text{ρ}_{\text{4}}}\text{ \textasciitilde}$ *Uniform* (0,1) ;

$\bar{\text{ρ}_{\text{5}}}\text{ \textasciitilde}$ *Uniform* (0,1); $\bar{\text{ρ}_{\text{6}}}\text{ \textasciitilde}$ *Uniform* (0,1).

- Temporal variability of recruitment (priors on the standard deviation, on the logit scale): $\text{σ}_{\text{ρ}_{\text{3}}}\text{ \textasciitilde}$ *Uniform* (0,10); $\text{σ}_{\text{ρ}_{\text{4}}}\text{\textasciitilde}$ *Uniform* (0,10); $\text{σ}_{\text{ρ}_{\text{5}}}\text{ \textasciitilde}$ *Uniform* (0,10) ;

$\text{σ}_{\text{ρ}_{\text{6}}}\text{\textasciitilde}$ *Uniform* (0,10).

- Mean breeding rate: $\bar{\text{ψ}_{\text{b}}}\text{ \textasciitilde}$ *Uniform* (0,1); $\bar{\text{ψ}_{\text{s}}}\text{ \textasciitilde}$ *Uniform* (0,1).
- Temporal variability of breeding rate (priors on the standard deviation, on the logit scale): $\text{σ}_{\text{ψ}_{\text{b}}}\text{ \textasciitilde}$ *Uniform* (0,10) ; $\text{σ}_{\text{ψ}_{\text{s}}}\text{ \textasciitilde}$ *Uniform* (0,10).
- Mean breeding success: $\bar{\text{π}_{\text{f}}}\text{ \textasciitilde}$ *Uniform* (0,2); $\bar{\text{π}_{\text{e}}}\text{ \textasciitilde}$ Uniform(0,2).
- Temporal variability of breeding success (priors on the standard deviation, on the log scale): $\text{σ}_{\text{π}_{\text{f}}}\text{ \textasciitilde}$ *Uniform* (0,10); $\text{σ}_{\text{π}_{\text{e}}}\text{ \textasciitilde}$ *Uniform* (0,10).
- Mean resighting probability: $\bar{\text{p}_{\text{y}}}\text{ \textasciitilde}$ *Uniform* (0,1); $\bar{\text{p}_{\text{p}}}\text{ \textasciitilde}$ *Uniform* (0,1) ;

$\text{p}_{\text{bs}}\text{ \textasciitilde}$ *Uniform* (0,1).

- Temporal variability of resighting probability (priors on the standard deviation, on the logit scale): $\text{σ}_{\text{p}_{\text{y}}}\text{ \textasciitilde}$ *Uniform* (0,10) ; $\text{σ}_{\text{p}_{\text{p}}}\text{ \textasciitilde}$ *Uniform* (0,10).
- Error of the count data (priors on the precision, i.e. inverse of the variance, on the log scale): $\text{τ}_{\text{obs}}\text{ \textasciitilde}$ *Gamma* (0.001,0.001).
- Initial number of individuals in each state (priors were truncated and rounded to positive integers): $\text{N}_{\text{Y,1}}\text{ \textasciitilde}$ *Normal* (506,100); $\text{N}_{\text{P2,1}}\text{ \textasciitilde}$ *Normal* (361,100);

$\text{N}_{\text{P3,1}}\text{ \textasciitilde}$ *Normal* (279,100); $\text{N}_{\text{P4,1}}\text{ \textasciitilde}$ *Normal* (149,100); $\text{N}_{\text{P5,1}}\text{ \textasciitilde}$ *Normal* (63,100); $\text{N}_{\text{P6,1}}\text{ \textasciitilde}$ *Normal* (19,100); $\text{N}_{\text{F,1}}\text{ \textasciitilde}$ *Normal* (264,100); $\text{N}_{\text{E,1}}\text{ \textasciitilde}$ *Normal* (1814,100);

$\text{N}_{\text{S,1}}\text{ \textasciitilde}$ *Normal* (264,100).

Note that for these normal priors, the mean value was selected according to the stable age distribution (see e.g. Szostek et al., 2014).

- Number of immigrants in each year (prior rounded to integer): $\text{N}_{\text{I,t}}\text{ \textasciitilde}$ *Uniform* (-5,1000).

### S2.7 BUGS code

|  |
| --- |
| model {  # --------------------------  # PARAMETERS IN THE MODEL  # --------------------------  # phi.0: first and second year survival probability  # phi.2: adult survival probability  # rho.3: probability to start breeding when 3 years old  # rho.4: probability to start breeding when 4 years old  # rho.5: probability to start breeding when 5 years old  # rho.6: probability to start breeding when 6 years old  # probability to start breeding when 7 years old is 1  # psi.b: probability that a breeder at t breeds at t+1  # psi.s: probability that a skipper at t breeds at t+1  # pi.f: breeding success of first-time breeders  # pi.e: breeding success of experienced breeders  # p.y: recapture probability of yearlings  # p.p: recapture probability of pre-breeders (age > 1)  # p.bs: recapture probability of breeders and skippers (very close to 1)  # ----------------------  # STATES & OBSERVATIONS  # ----------------------  # States (S):  # 1: fledgling  # 2: yearling  # 3: not yet breeding at age 2 years  # 4: not yet breeding at age 3 years  # 5: not yet breeding at age 4 years  # 6: not yet breeding at age 5 years  # 7: not yet breeding at age 6 years  # 8: first-time breeders  # 9: experienced breeders  # 10: sabbatical individuals  # 11: dead individuals  # Observations (O):  # 1: seen as fledgling  # 2: seen as prebreeder  # 3: seen as first-time breeder  # 4: seen as experienced breeder  # 5: seen as skipper  # 6: not seen  # ----------------------------------  # ----------------------------  # PRIORS AND CONSTRAINTS  # ----------------------------  for (t in 1:(n.occasions-1)) {    logit(phi.0[t]) <- ep.phi.0[t]  logit(phi.2[t]) <- ep.phi.2[t]  logit(rho.3[t]) <- ep.rho.3[t]  logit(rho.4[t]) <- ep.rho.4[t]  logit(rho.5[t]) <- ep.rho.5[t]  logit(rho.6[t]) <- ep.rho.6[t]  logit(psi.b[t]) <- ep.psi.b[t]  logit(psi.s[t]) <- ep.psi.s[t]  logit(p.y[t]) <- ep.p.y[t]  logit(p.p[t]) <- ep.p.p[t]    ep.phi.0[t] ~ dnorm(mu.phi.0, tau.phi.0)T(-10,10)  ep.phi.2[t] ~ dnorm(mu.phi.2, tau.phi.2)T(-10,10)  ep.rho.3[t] ~ dnorm(mu.rho.3, tau.rho.3)T(-10,10)  ep.rho.4[t] ~ dnorm(mu.rho.4, tau.rho.4)T(-10,10)  ep.rho.5[t] ~ dnorm(mu.rho.5, tau.rho.5)T(-10,10)  ep.rho.6[t] ~ dnorm(mu.rho.6, tau.rho.6)T(-10,10)  ep.psi.b[t] ~ dnorm(mu.psi.b, tau.psi.b)T(-10,10)  ep.psi.s[t] ~ dnorm(mu.psi.s, tau.psi.s)T(-10,10)  ep.p.y[t] ~ dnorm(mu.p.y, tau.p.y)T(-10,10)  ep.p.p[t] ~ dnorm(mu.p.p, tau.p.p)T(-10,10)    } #t  mean.phi.0 ~ dunif(0,1)  mu.phi.0 <- log(mean.phi.0 / (1-mean.phi.0))  mean.phi.2 ~ dunif(0,1)  mu.phi.2 <- log(mean.phi.2 / (1-mean.phi.2))  tau.phi.0 <- pow(sigma.phi.0, -2)  sigma.phi.0 ~ dunif(0,10)  var.phi.0 <- pow(sigma.phi.0, 2)  tau.phi.2 <- pow(sigma.phi.2, -2)  sigma.phi.2 ~ dunif(0,10)  var.phi.2 <- pow(sigma.phi.2, 2)  mean.rho.3 ~ dunif(0,1)  mu.rho.3 <- log(mean.rho.3 / (1-mean.rho.3))  mean.rho.4 ~ dunif(0,1)  mu.rho.4 <- log(mean.rho.4 / (1-mean.rho.4))  mean.rho.5 ~ dunif(0,1)  mu.rho.5 <- log(mean.rho.5 / (1-mean.rho.5))  mean.rho.6 ~ dunif(0,1)  mu.rho.6 <- log(mean.rho.6 / (1-mean.rho.6))  tau.rho.3 <- pow(sigma.rho.3, -2)  sigma.rho.3 ~ dunif(0,10)  var.rho.3 <- pow(sigma.rho.3, 2)  tau.rho.4 <- pow(sigma.rho.4, -2)  sigma.rho.4 ~ dunif(0,10)  var.rho.4 <- pow(sigma.rho.4, 2)  tau.rho.5 <- pow(sigma.rho.5, -2)  sigma.rho.5 ~ dunif(0,10)  var.rho.5 <- pow(sigma.rho.5, 2)  tau.rho.6 <- pow(sigma.rho.6, -2)  sigma.rho.6 ~ dunif(0,10)  var.rho.6 <- pow(sigma.rho.6, 2)  mean.psi.b ~ dunif(0,1)  mu.psi.b <- log(mean.psi.b / (1-mean.psi.b))  mean.psi.s ~ dunif(0,1)  mu.psi.s <- log(mean.psi.s / (1-mean.psi.s))  tau.psi.b <- pow(sigma.psi.b, -2)  sigma.psi.b ~ dunif(0,10)  var.psi.b <- pow(sigma.psi.b, 2)  tau.psi.s <- pow(sigma.psi.s, -2)  sigma.psi.s ~ dunif(0,10)  var.psi.s <- pow(sigma.psi.s, 2)  mean.p.y ~ dunif(0,1)  mu.p.y <- log(mean.p.y / (1-mean.p.y))  mean.p.p ~ dunif(0,1)  mu.p.p <- log(mean.p.p / (1-mean.p.p))  tau.p.y <- pow(sigma.p.y, -2)  sigma.p.y ~ dunif(0,10)  var.p.y <- pow(sigma.p.y, 2)  tau.p.p <- pow(sigma.p.p, -2)  sigma.p.p ~ dunif(0,10)  var.p.p <- pow(sigma.p.p, 2)  p.bs ~ dunif(0,1)  for (t in 1:n.occasions){  log(pi.f[t]) <- ep.pi.f[t]  log(pi.e[t]) <- ep.pi.e[t]  ep.pi.f[t] ~ dnorm(mu.pi.f, tau.pi.f)T(-10,10)  ep.pi.e[t] ~ dnorm(mu.pi.e, tau.pi.e)T(-10,10)    nrNI[t] ~ dunif(-5,1000)  NI[t] <- round(nrNI[t])  } #t  mean.pi.f ~ dunif(0, 2)  mu.pi.f <- log(mean.pi.f)  mean.pi.e ~ dunif(0, 2)  mu.pi.e <- log(mean.pi.e)  tau.pi.f <- pow(sigma.pi.f, -2)  sigma.pi.f ~ dunif(0,10)  var.pi.f <- pow(sigma.pi.f, 2)  tau.pi.e <- pow(sigma.pi.e, -2)  sigma.pi.e ~ dunif(0,10)  var.pi.e <- pow(sigma.pi.e, 2)  nrNY ~ dnorm(506,0.01)T(0,)  nrNP2 ~ dnorm(361,0.01)T(0,)  nrNP3 ~ dnorm(279,0.01)T(0,)  nrNP4 ~ dnorm(149,0.01)T(0,)  nrNP5 ~ dnorm(63,0.01)T(0,)  nrNP6 ~ dnorm(19,0.01)T(0,)  nrNF ~ dnorm(264,0.01)T(0,)  nrNE ~ dnorm(1814,0.01)T(0,)  nrNS ~ dnorm(264,0.01)T(0,)  NY[1] <- round(nrNY)  NP2[1] <- round(nrNP2)  NP3[1] <- round(nrNP3)  NP4[1] <- round(nrNP4)  NP5[1] <- round(nrNP5)  NP6[1] <- round(nrNP6)  NF[1] <- round(nrNF)  NE[1] <- round(nrNE)  NS[1] <- round(nrNS)  NB[1] <- NF[1]+NE[1]  tau.obs ~ dgamma(0.001, 0.001)  var.obs <- 1/tau.obs  sigma.obs <- pow(var.obs,0.5)  # -------------------------------------------------------  # LIKELIHOOD OF THE STATE-SPACE MODEL FOR COUNT DATA  # -------------------------------------------------------  ## State process  for (t in 1:(n.occasions-1)) {  NY[t+1] ~ dpois(mu1[t])  mu1[t] <- NF[t] * pi.f[t] * phi.0[t] + NE[t] * pi.e[t] * phi.0[t]  NP2[t+1] ~ dbin(mu2[t], NY[t])  mu2[t] <- phi.0[t]  NP3[t+1] ~ dbin(mu3[t], NP2[t])  mu3[t] <- phi.2[t] * (1-rho.3[t])  NP4[t+1] ~ dbin(mu4[t], NP3[t])  mu4[t] <- phi.2[t] * (1-rho.4[t])  NP5[t+1] ~ dbin(mu5[t], NP4[t])  mu5[t] <- phi.2[t] * (1-rho.5[t])  NP6[t+1] ~ dbin(mu6[t], NP5[t])  mu6[t] <- phi.2[t] * (1-rho.6[t])  NF3[t+1] ~ dbin(mu7[t], NP2[t])  mu7[t] <- phi.2[t] * rho.3[t]  NF4[t+1] ~ dbin(mu8[t], NP3[t])  mu8[t] <- phi.2[t] * rho.4[t]  NF5[t+1] ~ dbin(mu9[t], NP4[t])  mu9[t] <- phi.2[t] * rho.5[t]  NF6[t+1] ~ dbin(mu10[t], NP5[t])  mu10[t] <- phi.2[t] * rho.6[t]  NF7[t+1] ~ dbin(mu11[t], NP6[t])  mu11[t] <- phi.2[t]  # Note: IM[t] is given in another loop (that covers all time steps)  NEF[t+1] ~ dbin(mu12[t], NF[t])  mu12[t] <- phi.2[t] * psi.b[t]  NEE[t+1] ~ dbin(mu13[t], NE[t])  mu13[t] <- phi.2[t] * psi.b[t]  NES[t+1] ~ dbin(mu14[t], NS[t])  mu14[t] <- phi.2[t] * psi.s[t]  NSF[t+1] ~ dbin(mu15[t], NF[t])  mu15[t] <- phi.2[t] * (1-psi.b[t])  NSE[t+1] ~ dbin(mu16[t], NE[t])  mu16[t] <- phi.2[t] * (1-psi.b[t])  NSS[t+1] ~ dbin(mu17[t], NS[t])  mu17[t] <- phi.2[t] * (1-psi.s[t])  } #t  ## Observation process  for (t in 2:n.occasions) {  NF[t] <- NF3[t] + NF4[t] + NF5[t] + NF6[t] + NF7[t] + NI[t]  NE[t] <- NEF[t] + NEE[t] + NES[t]  NS[t] <- NSF[t] + NSE[t] + NSS[t]  NB[t] <- NF[t] + NE[t] # total breeding population size  } #t    for (t in 1:n.occasions) {  lNB[t] <- log(NB[t])  lC[t] <- log(C[t])  lC[t] ~ dnorm(lNB[t], tau.obs)    }    #---------------------------------------------------------  # LIKELIHOOD FOR REPRODUCTION DATA: POISSON REGRESSIONS  #---------------------------------------------------------  for (t in 1:n.occasions) {    JF[t] ~ dpois(mu.f[t])  log(mu.f[t]) <- log(RF[t]) + log(pi.f[t]*2)    JE[t] ~ dpois(mu.e[t])  log(mu.e[t]) <- log(RE[t]) + log(pi.e[t]*2)  JU[t] ~ dpois(rho.u[t])  log(mu.u[t]) <- log(RU[t])+log(2*(pi.f[t]*prop[t]+pi.e[t]*(1-prop[t])))    prop[t] <- NF[t]/NB[t]    } #t  # --------------------------------------------------------  # LIKELIHOOD OF THE MULTISTATE CAPTURE-RECAPTURE MODEL  # --------------------------------------------------------  ## Define state-transition and observation matrices  # Define probabilities of state Z(t+1) given Z(t)  for (t in 1:(n.occasions-1)){  ps[1,t,1] <- 0  ps[1,t,2] <- phi.0[t]  ps[1,t,3] <- 0  ps[1,t,4] <- 0  ps[1,t,5] <- 0  ps[1,t,6] <- 0  ps[1,t,7] <- 0  ps[1,t,8] <- 0  ps[1,t,9] <- 0  ps[1,t,10] <- 0  ps[1,t,11] <- 1-phi.0[t]    ps[2,t,1] <- 0  ps[2,t,2] <- 0  ps[2,t,3] <- phi.0[t]  ps[2,t,4] <- 0  ps[2,t,5] <- 0  ps[2,t,6] <- 0  ps[2,t,7] <- 0  ps[2,t,8] <- 0  ps[2,t,9] <- 0  ps[2,t,10] <- 0  ps[2,t,11] <- 1-phi.0[t]    ps[3,t,1] <- 0  ps[3,t,2] <- 0  ps[3,t,3] <- 0  ps[3,t,4] <- phi.2[t] * (1-rho.3[t])  ps[3,t,5] <- 0  ps[3,t,6] <- 0  ps[3,t,7] <- 0  ps[3,t,8] <- phi.2[t] * rho.3[t]  ps[3,t,9] <- 0  ps[3,t,10] <- 0  ps[3,t,11] <- 1-phi.2[t]  ps[4,t,1] <- 0  ps[4,t,2] <- 0  ps[4,t,3] <- 0  ps[4,t,4] <- 0  ps[4,t,5] <- phi.2[t] * (1-rho.4[t])  ps[4,t,6] <- 0  ps[4,t,7] <- 0  ps[4,t,8] <- phi.2[t] * rho.4[t]  ps[4,t,9] <- 0  ps[4,t,10] <- 0  ps[4,t,11] <- 1-phi.2[t]    ps[5,t,1] <- 0  ps[5,t,2] <- 0  ps[5,t,3] <- 0  ps[5,t,4] <- 0  ps[5,t,5] <- 0  ps[5,t,6] <- phi.2[t] * (1-rho.5[t])  ps[5,t,7] <- 0  ps[5,t,8] <- phi.2[t] * rho.5[t]  ps[5,t,9] <- 0  ps[5,t,10] <- 0  ps[5,t,11] <- 1-phi.2[t]    ps[6,t,1] <- 0  ps[6,t,2] <- 0  ps[6,t,3] <- 0  ps[6,t,4] <- 0  ps[6,t,5] <- 0  ps[6,t,6] <- 0  ps[6,t,7] <- phi.2[t] * (1-rho.6[t])  ps[6,t,8] <- phi.2[t] * rho.6[t]  ps[6,t,9] <- 0  ps[6,t,10] <- 0  ps[6,t,11] <- 1-phi.2[t]    ps[7,t,1] <- 0  ps[7,t,2] <- 0  ps[7,t,3] <- 0  ps[7,t,4] <- 0  ps[7,t,5] <- 0  ps[7,t,6] <- 0  ps[7,t,7] <- 0  ps[7,t,8] <- phi.2[t]  ps[7,t,9] <- 0  ps[7,t,10] <- 0  ps[7,t,11] <- 1-phi.2[t]    ps[8,t,1] <- 0  ps[8,t,2] <- 0  ps[8,t,3] <- 0  ps[8,t,4] <- 0  ps[8,t,5] <- 0  ps[8,t,6] <- 0  ps[8,t,7] <- 0  ps[8,t,8] <- 0  ps[8,t,9] <- phi.2[t] * psi.b[t]  ps[8,t,10] <- phi.2[t] * (1-psi.b[t])  ps[8,t,11] <- 1-phi.2[t]    ps[9,t,1] <- 0  ps[9,t,2] <- 0  ps[9,t,3] <- 0  ps[9,t,4] <- 0  ps[9,t,5] <- 0  ps[9,t,6] <- 0  ps[9,t,7] <- 0  ps[9,t,8] <- 0  ps[9,t,9] <- phi.2[t] * psi.b[t]  ps[9,t,10] <- phi.2[t] * (1-psi.b[t])  ps[9,t,11] <- 1-phi.2[t]  ps[10,t,1] <- 0  ps[10,t,2] <- 0  ps[10,t,3] <- 0  ps[10,t,4] <- 0  ps[10,t,5] <- 0  ps[10,t,6] <- 0  ps[10,t,7] <- 0  ps[10,t,8] <- 0  ps[10,t,9] <- phi.2[t] * psi.s[t]  ps[10,t,10] <- phi.2[t] * (1-psi.s[t])  ps[10,t,11] <- 1-phi.2[t]    ps[11,t,1] <- 0  ps[11,t,2] <- 0  ps[11,t,3] <- 0  ps[11,t,4] <- 0  ps[11,t,5] <- 0  ps[11,t,6] <- 0  ps[11,t,7] <- 0  ps[11,t,8] <- 0  ps[11,t,9] <- 0  ps[11,t,10] <- 0  ps[11,t,11] <- 1  # Define probabilities of O(t) given Z(t)  po[1,t,1] <- 1  po[1,t,2] <- 0  po[1,t,3] <- 0  po[1,t,4] <- 0  po[1,t,5] <- 0  po[1,t,6] <- 0    po[2,t,1] <- 0  po[2,t,2] <- p.y[t]  po[2,t,3] <- 0  po[2,t,4] <- 0  po[2,t,5] <- 0  po[2,t,6] <- 1-p.y[t]  po[3,t,1] <- 0  po[3,t,2] <- p.p[t]  po[3,t,3] <- 0  po[3,t,4] <- 0  po[3,t,5] <- 0  po[3,t,6] <- 1-p.p[t]    po[4,t,1] <- 0  po[4,t,2] <- p.p[t]  po[4,t,3] <- 0  po[4,t,4] <- 0  po[4,t,5] <- 0  po[4,t,6] <- 1-p.p[t]  po[5,t,1] <- 0  po[5,t,2] <- p.p[t]  po[5,t,3] <- 0  po[5,t,4] <- 0  po[5,t,5] <- 0  po[5,t,6] <- 1-p.p[t]    po[6,t,1] <- 0  po[6,t,2] <- p.p[t]  po[6,t,3] <- 0  po[6,t,4] <- 0  po[6,t,5] <- 0  po[6,t,6] <- 1-p.p[t]    po[7,t,1] <- 0  po[7,t,2] <- p.p[t]  po[7,t,3] <- 0  po[7,t,4] <- 0  po[7,t,5] <- 0  po[7,t,6] <- 1-p.p[t]    po[8,t,1] <- 0  po[8,t,2] <- 0  po[8,t,3] <- p.bs  po[8,t,4] <- 0  po[8,t,5] <- 0  po[8,t,6] <- 1-p.bs    po[9,t,1] <- 0  po[9,t,2] <- 0  po[9,t,3] <- 0  po[9,t,4] <- p.bs  po[9,t,5] <- 0  po[9,t,6] <- 1-p.bs    po[10,t,1] <- 0  po[10,t,2] <- 0  po[10,t,3] <- 0  po[10,t,4] <- 0  po[10,t,5] <- p.bs  po[10,t,6] <- 1-p.bs  po[11,t,1] <- 0  po[11,t,2] <- 0  po[11,t,3] <- 0  po[11,t,4] <- 0  po[11,t,5] <- 0  po[11,t,6] <- 1  } #t    # Likelihood  for (i in 1:n.ind){  # Define latent state at first capture in the histories  z[i,f[i]] <- rs[i]  # the vector f contains the year of first capture for each individual  # the vector rs contains the state at first capture for each individual    for (t in (f[i]+1):n.occasions){  # State process: draw z(t) given z(t-1)  z[i,t] ~ dcat(ps[z[i,t-1],t-1,])  # Observation process: draw o(t) given z(t)  o[i,t] ~ dcat(po[z[i,t],t-1,])  } #t  } #i  } #model |
|  |

### S2.8 References

Baker, M. B., & Rao, S. (2004). Incremental costs and benefits shape natal dispersal: theory and example with Hemilepistus reaumuri. *Ecology, 85*(4), 1039-1051. <https://doi.org/10.1890/02-0507>

Barbraud, C., & Delord, K. (2021). Selection against immigrants in wild seabird populations. Ecology Letters, 24(1), 84-93. <https://doi.org/10.1111/ele.13624>

Bonte, D., Van Dyck, H., Bullock, J. M., Coulon, A., Delgado, M., Gibbs, M., ... & Travis, J. M. (2012). Costs of dispersal. *Biological Reviews, 87*(2), 290-312. <https://doi.org/10.1111/j.1469-185X.2011.00201.x>

Burgess, S. C., Treml, E. A., & Marshall, D. J. (2012). How do dispersal costs and habitat selection influence realized population connectivity?. *Ecology, 93*(6), 1378-1387. <https://doi.org/10.1890/11-1656.1>

Burnham, K. P., & White, G. C. (2002). Evaluation of some random effects methodology applicable to bird ringing data. *Journal of Applied Statistics, 29*(1-4), 245-264. <https://doi.org/10.1080/02664760120108755>

Clobert, J., M. Baguette, T. G. Benton, J. M. Bullock, and S. Ducatez. 2012. *Dispersal ecology and evolution*. Oxford University Press, Oxford, UK.

Frederiksen, M., Moe, B., Daunt, F., Phillips, R. A., Barrett, R. T., Bogdanova, M. I., ... & Anker‐Nilssen, T. (2012). Multicolony tracking reveals the winter distribution of a pelagic seabird on an ocean basin scale. *Diversity and distributions, 18*(6), 530-542. <https://doi.org/10.1111/j.1472-4642.2011.00864.x>

Gimenez, O., Rossi, V., Choquet, R., Dehais, C., Doris, B., Varella, H., ... & Pradel, R. (2007). State-space modelling of data on marked individuals. *Ecological Modelling, 206*(3-4), 431-438. <https://doi.org/10.1016/j.ecolmodel.2007.03.040>

Kéry, M., & Schaub, M. (2011). *Bayesian population analysis using WinBUGS: a hierarchical perspective*. Academic Press, London, UK.

Lebreton, J. D., Nichols, J. D., Barker, R. J., Pradel, R., & Spendelow, J. A. (2009). Modeling individual animal histories with multistate capture–recapture models. *Advances in Ecological Research, 41*, 87-173. <https://doi.org/10.1016/S0065-2504(09)00403-6>

Lenormand, T. (2002). Gene flow and the limits to natural selection. Trends in Ecology & *Evolution, 17*(4), 183-189. <https://doi.org/10.1016/S0065-2504(09)00403-6>

Oro, D., Tavecchia, G., & Genovart, M. (2011). Comparing demographic parameters for philopatric and immigrant individuals in a long-lived bird adapted to unstable habitats. Oecologia, 165(4), 935-945. <https://doi.org/10.1007/s00442-010-1773-3>

Phillips, B. L., Brown, G. P., Travis, J. M., & Shine, R. (2008). Reid’s paradox revisited: the evolution of dispersal kernels during range expansion. *The American Naturalist, 172*(S1), S34-S48. <https://doi.org/10.1086/588255>

Ponchon, A., Gremillet, D., Doligez, B., Chambert, T., Tveraa, T., González‐Solís, J., & Boulinier, T. (2013). Tracking prospecting movements involved in breeding habitat selection: insights, pitfalls and perspectives. *Methods in Ecology and Evolution, 4*(2), 143-150. <https://doi.org/10.1111/j.2041-210x.2012.00259.x>

Szostek, K. L., Schaub, M., & Becker, P. H. (2014). Immigrants are attracted by local pre‐breeders and recruits in a seabird colony. *Journal of Animal Ecology, 83*(5), 1015-1024. <https://doi.org/10.1111/1365-2656.12206>

De Valpine, P., & Hastings, A. (2002). Fitting population models incorporating process noise and observation error. *Ecological Monographs, 72*(1), 57-76. [https://doi.org/10.1890/0012-9615(2002)072[0057:FPMIPN]2.0.CO;2](https://doi.org/10.1890/0012-9615(2002)072%5b0057:FPMIPN%5d2.0.CO;2)

## APPENDIX S3

## Summaries of the posterior distributions

**Contents:**

**S3.1 Foreword of the section (p. 36)**

**S3.2 Demographic parameters (p. 38)**

**S3.3 Resighting probabilities and observation errors on count data (p. 42)**

**S3.4 References (p. 43)**

### S3.1 Foreword of the section

The model was run from R (R Core Team 2016) using package *rjags* (Plummer, 2015). We ran 20 chains, discarded the first 15,000 iterations and used the subsequent 35,000 iterations for posterior exploration (700,000 samples − chains were not thinned). Convergence was assessed using the Brooks-Gelman-Rubin diagnostic $\hat{\text{R}}$ (Brooks & Gelman, 1998; see below) and achieved with all $\hat{\text{R}}$<1.02. Monte Carlo standard errors were small enough to report posterior means of demographic rates with three decimal place precision (see below). Each 95% credible interval (‘95%CRI’) was calculated as the smallest interval containing 95% of the posterior samples (‘highest posterior density method’).

This appendix contains summaries for the following demographic parameters: local survival, local recruitment, breeding rate of former breeders and skippers, breeding success (Fig. S9 to S12). Second, we give summaries for resighting probabilities (Fig. S13) and observation error for count data. Due to the very large number of parameters involved, we provide only a graphical summary for year-specific parameters (with posterior means and 95% credible intervals in each year). When year variation was specified with random effects (see Appendix S2), we provide temporal means and standard deviations (along with 95%CI between brackets) as a note to the figure. We used the R packages CODA (Plummer et al., 2006) and MCMCglmm (Hadfield, 2010) for post-processing of the MCMC chains.

We also give some details on the Brooks-Gelman-Rubin diagnostic $\hat{\text{R}}$, the Monte Carlo standard error (MCSE), and the effective sample size (ESS). In brief, $\hat{\text{R}}$ is a measure of convergence of the MCMC chains (Brooks & Gelman, 1998); the closer to one, the better. In practice, one considers that convergence is achieved when $\hat{\text{R}}$<1.1. MCSE is a measure of the error due to sampling, because MCMC chains are dependent sequences of pseudo-random draws (Lunn et al. 2012). This error increases with autocorrelation between MCMC samples and decreases with the number of samples. MCSE thus indicates the appropriate decimal place precision: the actual mean is approximately within ±2×MCSE around the posterior mean with probability 0.95, within ±2.6×MCSE with probability 0.99, etc. The ESS quantifies the number of independent samples that would contain the same information as the dependent MCMC samples (Lunn et al,. 2012). According to Raftery & Lewis (1992), ESS=4000 is sufficient for well-behaved posterior distributions to provide the 2.5% quantiles within $\text{±}$0.005 with probability 0.95 (i.e. reported 95% credible intervals then have posterior probability within [0.94,0.96]).

### S3.2 Demographic parameters


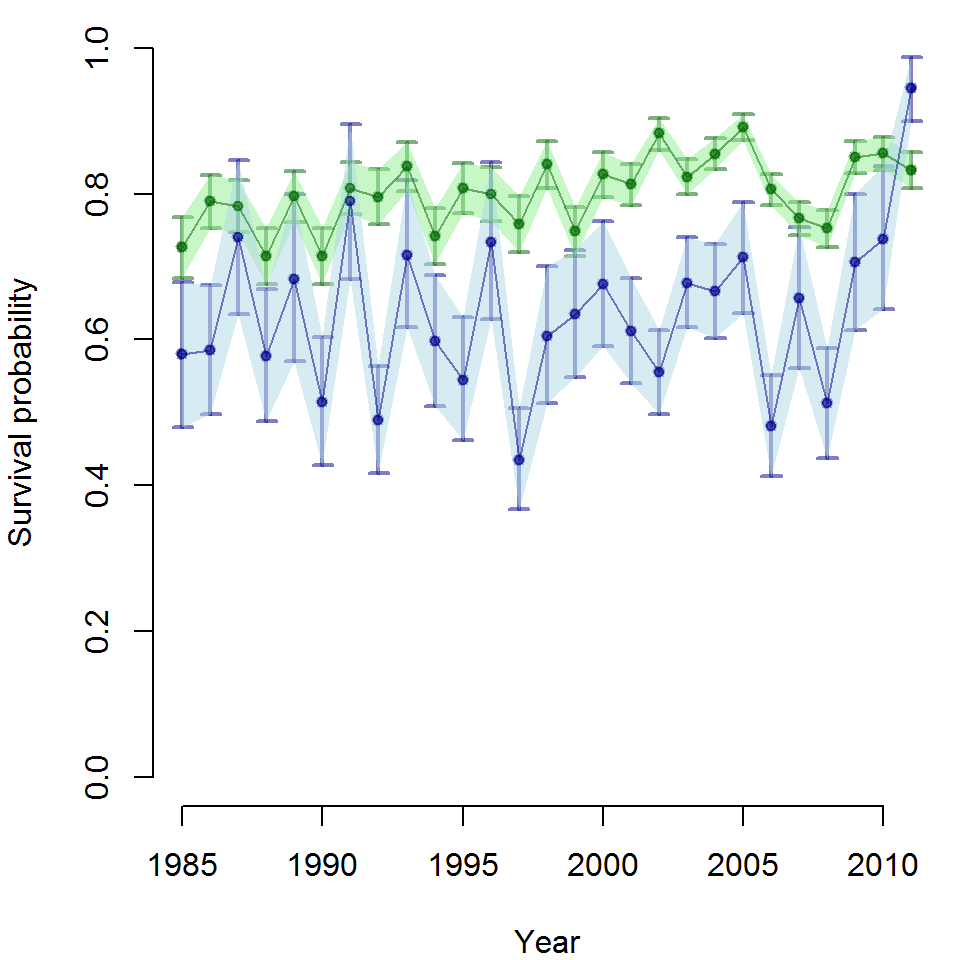


**Figure S9**. Estimates of kittiwake apparent survival probabilities across 1985−2011 in the Cap Sizun population. Annual survival between age 0 and age 2 is in blue. Annual survival between from age 2 is in green. Points indicate posterior means. Color backgrounds and segments indicate 95%CI. The mean annual survival at age 0 and age 1 ($\bar{\text{ϕ}_{\text{0}}}$) was 0.65 [0.59,0.71], temporal standard deviation on the logit scale ($\text{σ}_{\text{ϕ}_{\text{0}}}$) was 0.69 [0.44,0.98]. The mean annual survival from age 2 ($\bar{\text{ϕ}_{\text{2}}}$) was 0.81 [0.78,0.83], temporal standard deviation on the logit scale ($\text{σ}_{\text{ϕ}_{\text{2}}}$) was 0.35 [0.25,0.46]. All $\hat{\text{R}}$ < 1.002, all MCSE ≤ 0.001, all ESS > 7547.


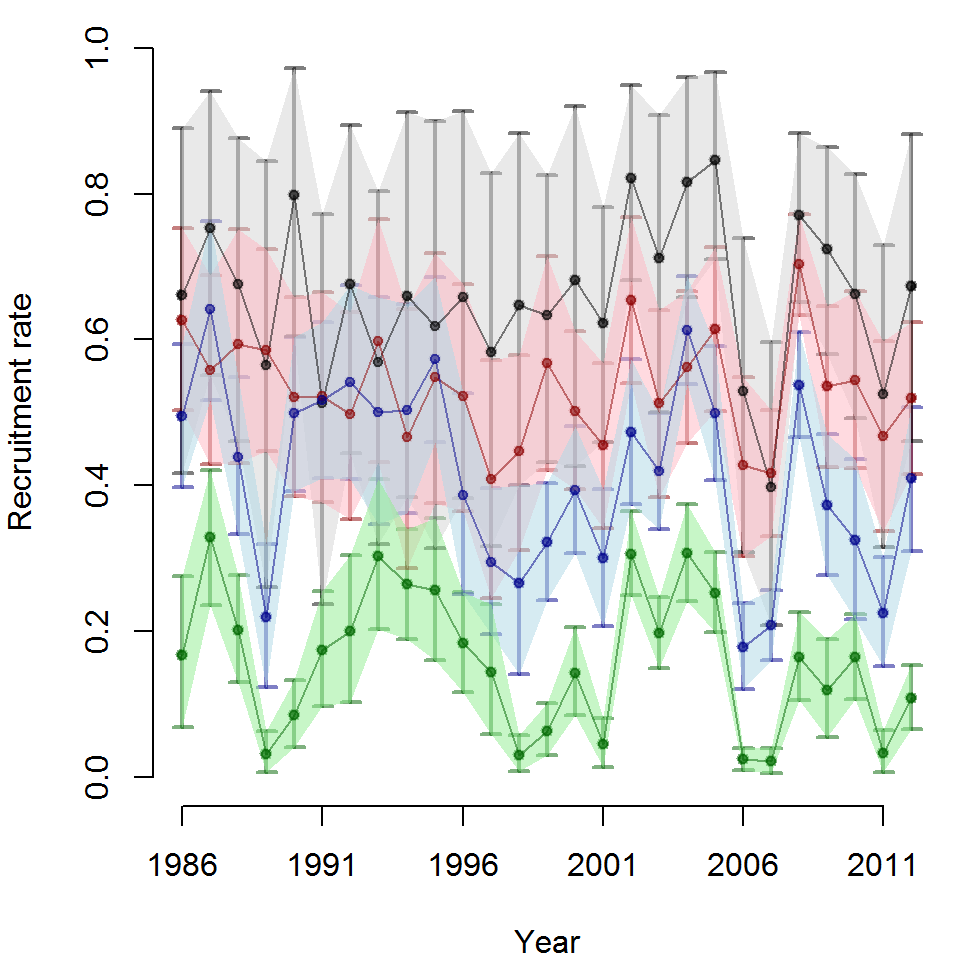


**Figure S10**. Estimates of kittiwake local recruitment probability across 1986−2012 in the Cap Sizun population. Recruitment at age 3 is in green, recruitment at age 4 is in blue, recruitment at age 5 is in red, recruitment at age 6 is in grey; recruitment rate at age 7 is 1. Color backgrounds and segments indicate 95%CI. The mean recruitment rate at age 3 was $\bar{\text{ρ}_{\text{3}}}$ = 0.13 [0.08,0.18], with temporal standard deviation (on the logit scale) $\text{σ}_{\text{ρ}_{\text{3}}}$ = 1.11 [0.77,1.48]. The mean recruitment rate at age 3 was $\bar{\text{ρ}_{\text{4}}}$ = 0.41 [0.34,0.47], with temporal standard deviation $\text{σ}_{\text{ρ}_{\text{4}}}$ = 0.66 [0.46,0.88]. The mean recruitment rate at age 3 was $\bar{\text{ρ}_{\text{5}}}$ = 0.53 [0.48,0.59], with temporal standard deviation $\text{σ}_{\text{ρ}_{\text{5}}}$ = 0.43 [0.24,0.63]. The mean recruitment rate at age 3 was $\bar{\text{ρ}_{\text{6}}}$ = 0.67 [0.58,0.76], with temporal standard deviation $\text{σ}_{\text{ρ}_{\text{6}}}$ = 0.78 [0.38,0.1.23]. All $\hat{\text{R}}$ < 1.002, all MCSE ≤ 0.001, all ESS > 13003.


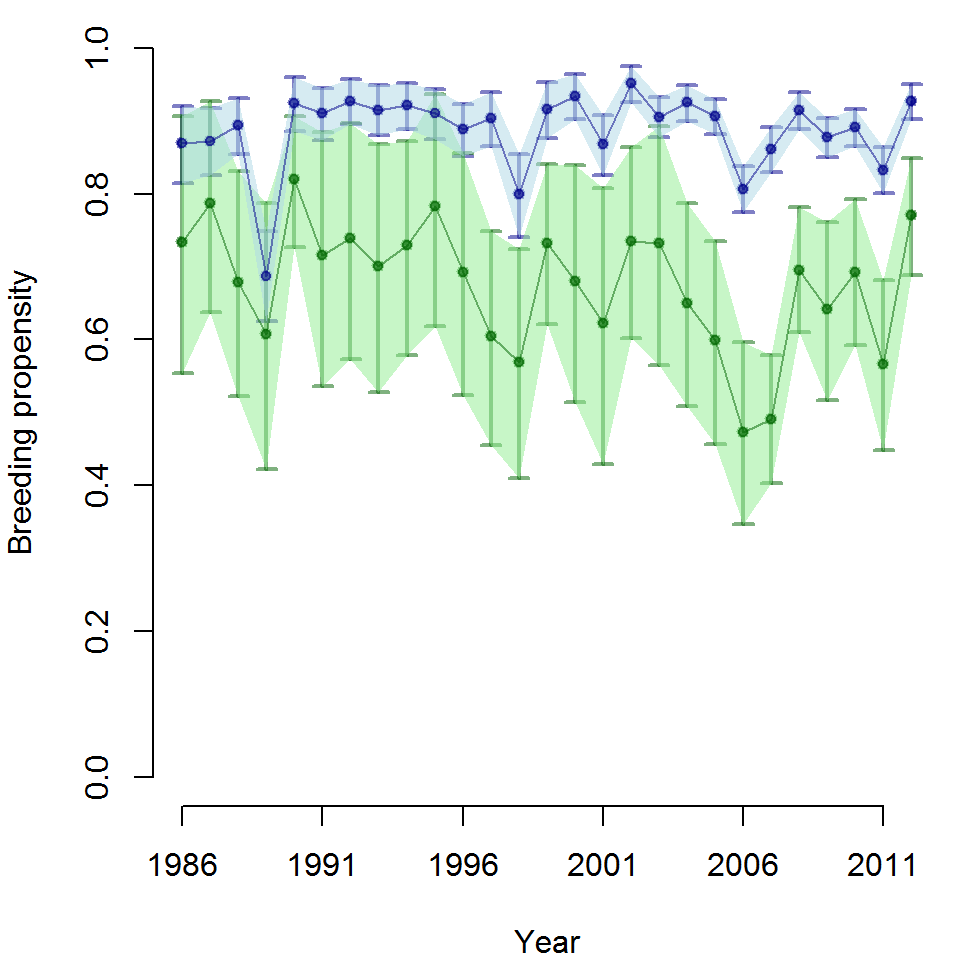


**Figure S11**. Estimates of kittiwake breeding rate across 1986−2012 in the Cap Sizun population. Breeding propensity of former breeders is in blue. Breeding rate of former skippers is in green. Points indicate posterior means. Color backgrounds and segments indicate 95%CI. The mean breeding rate of former breeder ($\bar{\text{ψ}_{\text{b}}}$) was 0.90 [0.87,0.92], temporal standard deviation on the logit scale ($\text{σ}_{\text{ψ}_{\text{b}}}$) was 0.53 [0.37,0.71]. The mean annual breeding rate of former skippers ($\bar{\text{ψ}_{\text{s}}}$) was 0.69 [0.62,0.75], temporal standard deviation on the logit scale ($\text{σ}_{\text{ψ}_{\text{s}}}$) was 0.57 [0.33,0.85]. All $\hat{\text{R}}$ < 1.002, all MCSE ≤ 0.001, all ESS > 9218.


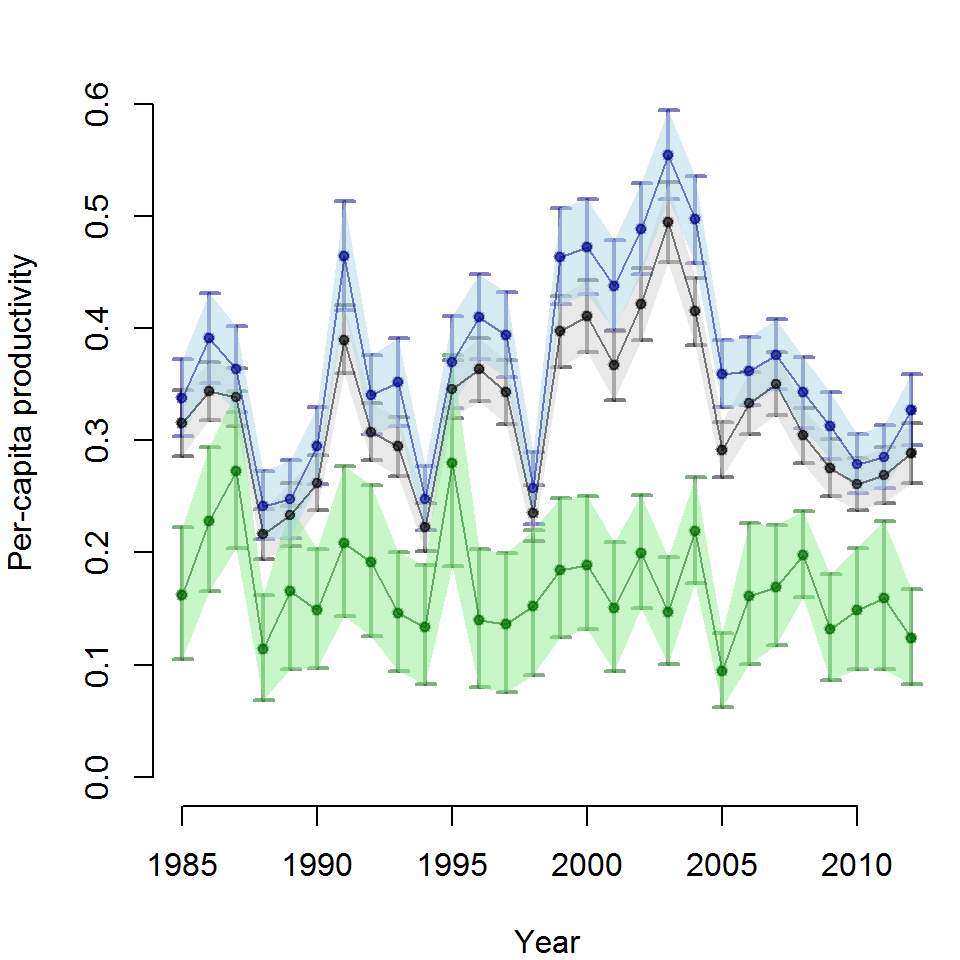


**Figure S12**. Estimates of kittiwake *per capita* breeding success rates across 1986−2012 in the Cap Sizun population. The breeding success of pairs formed by two former breeders is in blue. The breeding success of pairs formed by two first-time breeders is in green. The breeding success of pairs form by two breeders of different breeding experience, or at least one breeder of unknown status is in grey. This latter breeding success was assumed to be an average of breeding success of inexperienced and experienced pairs weighted by their respective proportion among breeders. Points indicate posterior means. Color backgrounds and segments indicate 95%CI. The mean breeding success of first-time breeders ($\bar{\text{π}_{\text{f}}}$) was 0.16 [0.14,0.19], temporal standard deviation on the log scale ($\text{σ}_{\text{π}_{\text{f}}}$) was 0.32 [0.20,0.46]. The mean breeding success of experienced breeders ($\bar{\text{π}_{\text{e}}}$) was 0.36 [0.33,0.40], temporal standard deviation on the log scale ($\text{σ}_{\text{π}_{\text{e}}}$) was 0.25 [0.18,0.32]. All $\hat{\text{R}}$ < 1.002, all MCSE < 0.001, all ESS > 14557.

### S3.3 Resighting probabilities and observation errors on count data


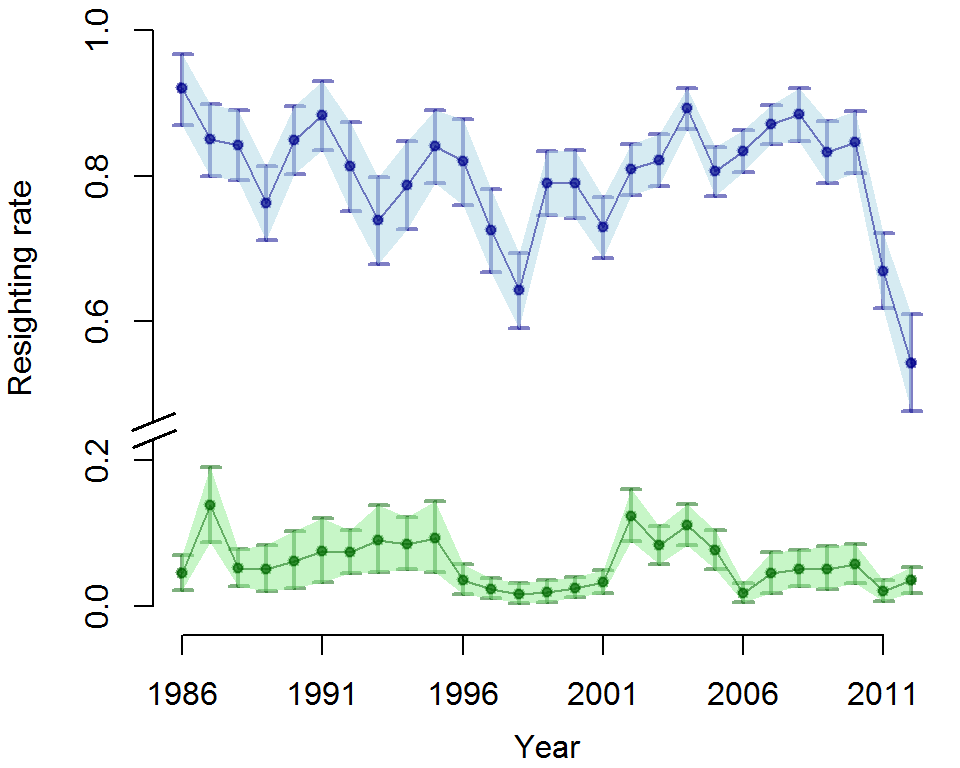


**Figure S13**. Estimates of kittiwake resighting probability across 1986−2011 in the Cap Sizun population, for yearlings (green) and prebreeders (blue). Points indicate posterior means. Color backgrounds and segments indicate 95%CI. The mean resighting probability of yearlings ($\bar{\text{p}_{\text{y}}}$) was 0.050 [0.036,0.065], temporal standard deviation on the logit scale ($\text{σ}_{\text{p}_{\text{y}}}$) was 0.81 [0.78,0.84]. The mean resighting probability of prebreeders ($\bar{\text{p}_{\text{p}}}$) was 0.81 [0.78,0.83], temporal standard deviation on the logit scale ($\text{σ}_{\text{p}_{\text{p}}}$) was 0.56 [0.39,0.75]. All $\hat{\text{R}}$ < 1.001, all MCSE ≤ 0.0005, all ESS > 15295.

Resighting probability of breeders and skippers ($\text{p}_{\text{bs}}$ − not shown in Figure S13) was 0.998 [0.997,0.999] ($\hat{\text{R}}$ < 1.0001, MCSE < 0.0001, ESS = 348976).

Observation error for count data on the log scale ($\text{σ}_{\text{obs}}$) was 0.051 [0.016,0.097] ($\hat{\text{R}}$ = 1.017, MCSE < 0.001, ESS = 1282).

### S3.4 References

Brooks, S. P., & Gelman, A. (1998). General methods for monitoring convergence of iterative simulations. *Journal of computational and graphical statistics, 7*(4), 434-455. <https://doi.org/10.1080/10618600.1998.10474787#.YC5tXehKizV>

Hadfield, J. D. (2010). MCMC methods for multi-response generalized linear mixed models: the MCMCglmm R package. *Journal of statistical software, 33*(2), 1-22. <https://10.18637/jss.v033.i02>

Lunn, D., C. Jackson, N. Best, A. Thomas, and D. Spiegelhalter. 2012. *The BUGS book: A practical introduction to Bayesian analysis*. CRC press, New York, USA.

Plummer, M., Best, N., Cowles, K., & Vines, K. (2006). CODA: convergence diagnosis and output analysis for MCMC. *R news, 6*(1), 7-11.

Raftery, A. E., and S. Lewis. 1992. How many iterations in the Gibbs sampler? Pages 763–773 *in* Bernardo J. M., Berger J. O., Dawid A. P., and Smith A. F. M., editors. *Bayesian Statistics* (4^th^ ed.). Oxford University Press, Oxford, UK.

## APPENDIX S4

## Posterior predictive checks

**Contents:**

**S4.1 Foreword of the section (p. 44)**

**S4.2 Posterior predictive checks for count and reproduction data (p. 44)**

**S4.3 References (p. 53)**

### S4.1 Foreword of the section

Data replication (i.e. predictions derived from posterior samples of the model), and other calculations involved in posterior checks were performed aside from the main MCMC sampling to save computer resources. We used the same number of iterations in the burnin and monitoring phase as for estimation of parameters (see *Materials and Methods*) but we used only 10 chains, yielding a total of 3.5×10^5^ samples at the end. Hereafter we describe methods to get discrepancy measures and posterior-predictive p-values for the submodels for count data and reproduction data.

### S4.2 Posterior predictive checks for the submodels for count and reproduction data

***S4.2.1 Methods***

Following Gelman et al. (1996), we used the χ² discrepancy metric to assess the overall goodness of fit. The χ² discrepancy metric ($\text{D}_{\text{χ}^{\text{2}}}$) is the sum of squared Pearson residuals; $\text{D}_{\text{χ}^{\text{2}}}^{\text{obs}}$ quantifies the distance of observed data to the model, and $\text{D}_{\text{χ}^{\text{2}}}^{\text{rep}}$ quantifies the distance of replicated data to the model:

$$D_{\chi^{2}}^{obs}=\sum_{i=1}^{n} \frac{{(y_{i}^{obs} - E(y_{i}|\theta))}^{2}}{Var(y_{i}|\theta)}$$

$$D_{\chi^{2}}^{rep}=\sum_{i=1}^{n} \frac{{(y_{i}^{rep} - E(y_{i}|\theta))}^{2}}{Var(y_{i}|\theta)}$$

where *n* is the number of observations, $\text{y}$ designates the response variable, $\text{y}_{\text{i}}^{\text{obs}}$ is the *i*th observation, $\text{y}_{\text{i}}^{\text{rep}}$ is the prediction (replicate) for the *i*th observation, $\text{θ}$ is the parameter vector (i.e. the model), *E* is the expectation (i.e. $\text{E}\text{(}\text{y}_{\text{i}}\text{|θ}\text{)}$ is the model expectation for $\text{y}_{\text{i}}$) and *Var* is the variance (i.e. $\text{Var}\text{(}\text{y}_{\text{i}}\text{|θ}\text{)}$ is the variance of the response variable according to the model).

The piece of code to get data replicates and discrepancy measures for the submodels for count data and reproduction data is given below (this code is to be added to the original code given in Appendix S2):

| ## This piece of code has to be added to the main code (see Appendix S2)  for (t in 1:n.occasion) {  ## Calculations for posterior predictive checks for count data:    # data replicates  pred.lC[t] ~ dnorm(lNB[t],tau.obs)    # discrepancy measures  # distances from replicates to the model  D.lC[t,1] <- ((pred.lC[t]-lNB[t])*(pred.lC[t]-lNB[t]))/var.obs  # distances from observations to the model  D.lC[t,2] <- ((lC[t]-lNB[t])*(lC[t]-lNB[t]))/var.obs  ## Calculations for posterior predictive checks for reproduction data:  # data replicates  pred.JF[t] ~ dpois(mu.f[t])  pred.JE[t] ~ dpois(mu.e[t])  pred.JU[t] ~ dpois(mu.u[t])  # discrepancy measures  # distances from replicates to the model  D.JF[t,1] <- ((pred.JF[t]-mu.f[t])*(pred.JF[t]-mu.f[t]))/mu.f[t]  D.JE[t,1] <- ((pred.JB[t]-mu.e[t])*(pred.JE[t]-mu.e[t]))/mu.e[t]  D.JU[t,1] <- ((pred.JU[t]-mu.u[t])*(pred.JU[t]-mu.u[t]))/mu.u[t]  # distances from observations to the model  D.JF[t,2] <- ((JF[t]-mu.f[t])*(JF[t]-mu.f[t]))/mu.f[t]  D.JE[t,2] <- ((JB[t]-mu.e[t])*(JB[t]-mu.e[t]))/mu.e[t]  D.JU[t,2] <- ((JU[t]-mu.u[t])*(JU[t]-mu.u[t]))/mu.u[t]  } #t  # overall PPC  sumchi2[1,1] <- sum(D.C[,1])  sumchi2[2,1] <- sum(D.C[,2])  sumchi2[1,2] <- sum(D.JF[,1])  sumchi2[2,2] <- sum(D.JF[,2])  sumchi2[1,3] <- sum(D.JE[,1])  sumchi2[2,3] <- sum(D.JE[,2])  sumchi2[1,4] <- sum(D.JU[,1])  sumchi2[2,4] <- sum(D.JU[,2]) |
| --- |

The posterior predictive p-value (Gelman et al., 1996; ‘*PPp-value*’) is the probability that the distance of observed data to the model is greater than the distance of replicated data to the model (a value close to 0.5 suggests a model with a good fit, whereas a value close to 0 or 1 indicates substantial lack of fit):

*PPp-value =* Pr ($D_{\chi^{2}}^{rep} > D_{\chi^{2}}^{obs}$).

We *computed posterior predictive p-values across* all years and for each year, in the state-space model for count data and the Poisson regressions for reproduction data. Cross-validation (Green et al. 2009) was impracticable because of the huge computation time involved.

***S4.2.1 Results***

Fig. S14 provides a graphical assessment of the amount of $D_{\chi^{2}}^{rep}$ values higher than $D_{\chi^{2}}^{obs}$ values (both summed across all years in the data) for the state-space model and the three Poisson regressions, with corresponding *PPP-values*. Table S1 provides the *PPP-values* in each year. Further, we provide posterior predictive distributions (i.e. distribution of replicates) for each response in each year, plotted against the observation (Fig. S15 to 18).

Most *PPP-values* were close to 0.5 and away from 0 and 1, and none indicated a substantial lack of fit. Observed values were always clearly within the distribution of predicted values and usually at the median.


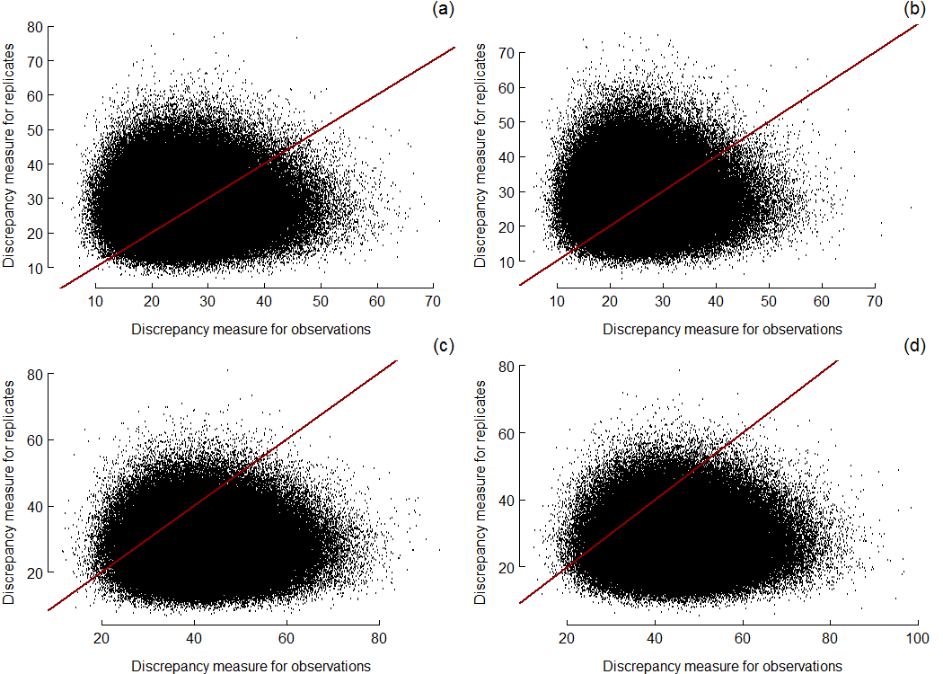


**Figure S14**. Discrepancy measures for replicates against discrepancy measures for observations summed across all years in all posterior samples. Panel (a) is for count data: *PPP-value =* 0.55. Panel (b) is for breeding success of pairs formed by two first-time breeders: *PPP-value =* 0.55. Panel (c) is for breeding success of pairs formed by two experienced breeders: *PPP-value =* 0.11. Panel (d) is for breeding success of pairs formed by two breeders of different experience, or at least one breeder of unknown experience: *PPP-value =* 0.08. In red is the x=y line. The relatively low values *PPP-values* in (c) and (d) − although not indicative of a lack of fit − seem to stem from one or two years in particular, for which the fit was less satisfying than in the other years (see Table S1, Fig. S17, S18).

**Table S1**. Posterior predictive p-values associated with χ^2^ discrepancies for the different data: breeding population counts, and breeding success of (F) pairs formed by two first-time breeders, (E) pairs formed by two experienced breeders, (U) pairs formed by two breeders of different experience or at least one breeder of unknown experience.

| Year | Data | | | |
| --- | --- | --- | --- | --- |
|  | Counts | Breeding success | | |
|  |  | F | E | U |
| 1985 | 0.90 | 0.53 | 0.34 | 0.45 |
| 1986 | 0.52 | 0.47 | 0.62 | 0.54 |
| 1987 | 0.50 | 0.36 | 0.59 | 0.55 |
| 1988 | 0.53 | 0.46 | 0.46 | 0.37 |
| 1989 | 0.52 | 0.48 | 0.51 | 0.38 |
| 1990 | 0.48 | 0.55 | 0.46 | 0.45 |
| 1991 | 0.52 | 0.49 | 0.59 | 0.53 |
| 1992 | 0.48 | 0.53 | 0.54 | 0.52 |
| 1993 | 0.43 | 0.51 | 0.22 | 0.32 |
| 1994 | 0.47 | 0.38 | 0.31 | 0.50 |
| 1995 | 0.51 | 0.38 | 0.04 | 0.11 |
| 1996 | 0.45 | 0.39 | 0.47 | 0.45 |
| 1997 | 0.53 | 0.54 | 0.37 | 0.45 |
| 1998 | 0.45 | 0.45 | 0.33 | 0.23 |
| 1999 | 0.48 | 0.52 | 0.58 | 0.52 |
| 2000 | 0.50 | 0.50 | 0.29 | 0.25 |
| 2001 | 0.54 | 0.50 | 0.56 | 0.52 |
| 2002 | 0.55 | 0.50 | 0.57 | 0.51 |
| 2003 | 0.38 | 0.49 | 0.49 | 0.35 |
| 2004 | 0.53 | 0.38 | 0.28 | 0.29 |
| 2005 | 0.52 | 0.38 | 0.40 | 0.37 |
| 2006 | 0.50 | 0.45 | 0.15 | 0.08 |
| 2007 | 0.55 | 0.52 | 0.53 | 0.56 |
| 2008 | 0.54 | 0.45 | 0.41 | 0.34 |
| 2009 | 0.52 | 0.55 | 0.33 | 0.20 |
| 2010 | 0.57 | 0.52 | 0.53 | 0.60 |
| 2011 | 0.55 | 0.56 | 0.56 | 0.61 |
| 2012 | 0.45 | 0.52 | 0.36 | 0.28 |


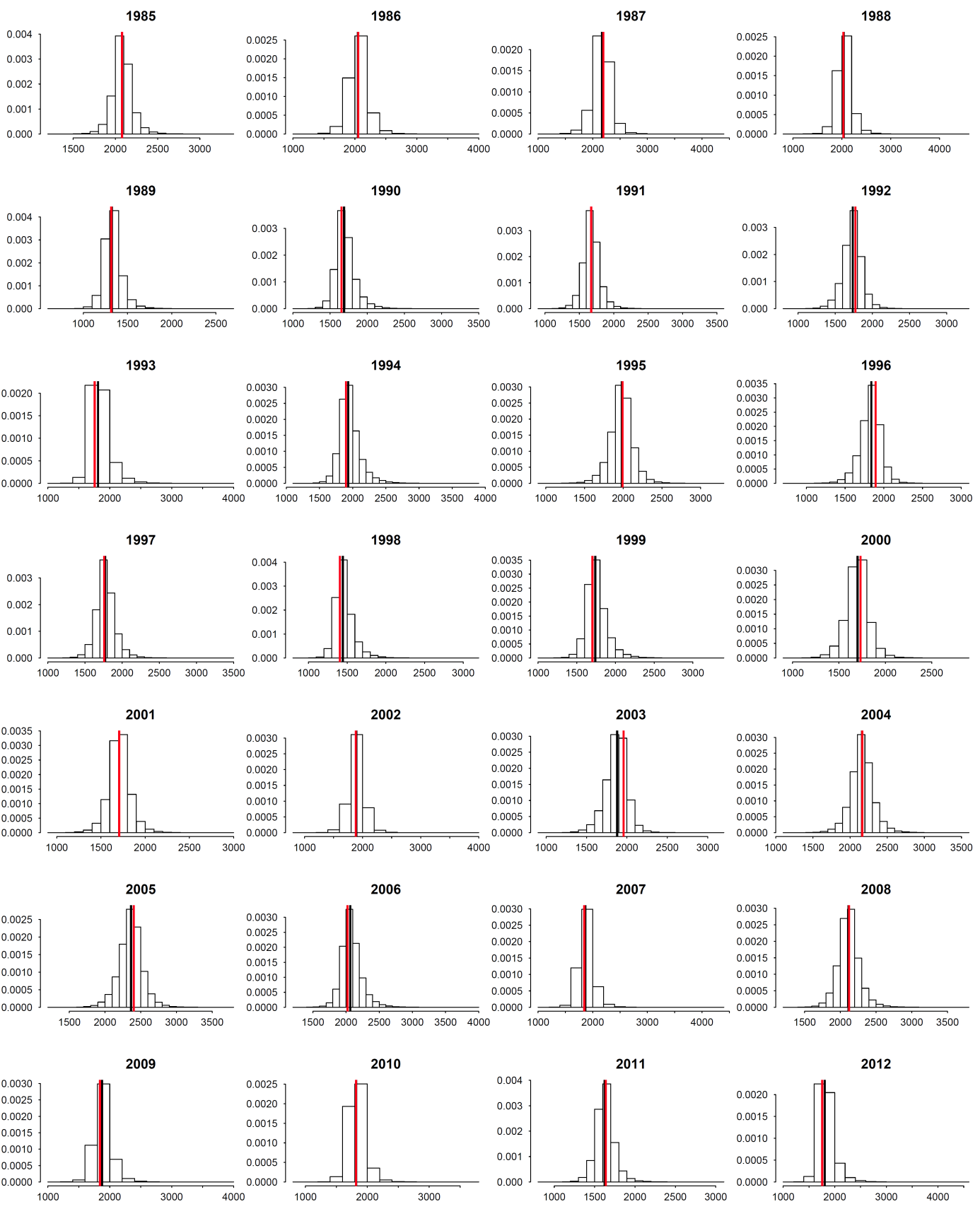


**Figure S15**. Posterior predictive distribution of the breeding population count in each year across the study period. In abscissa of the histogram is the value, in ordinate is the density. The red line indicates the observed value, the bold black line indicates the median.


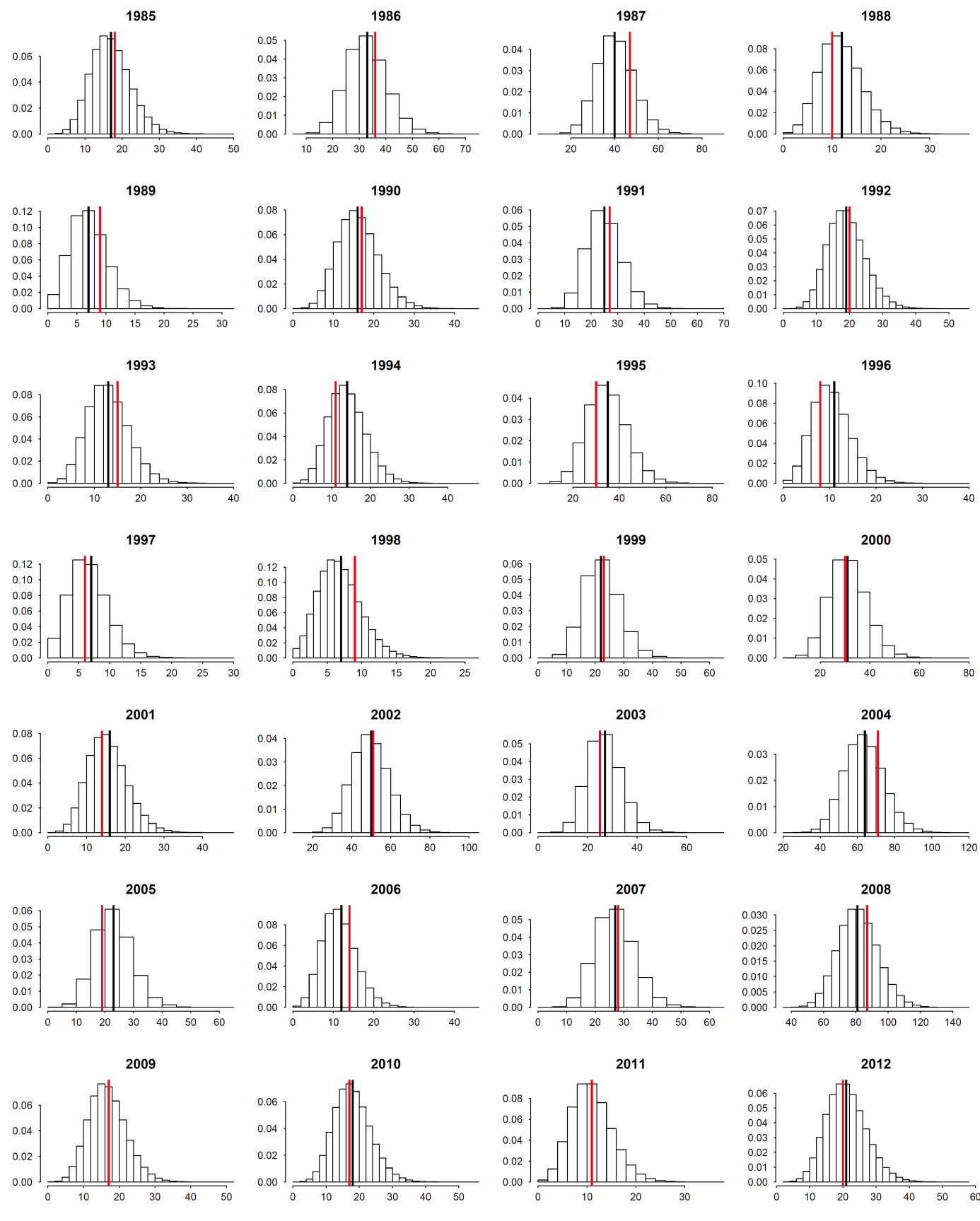


**Figure S16**. Posterior predictive distribution of the fledgling count from breeding pairs composed of two first-time breeders in each year across the study period. In abscissa of the histogram is the value, in ordinate is the density. The red line indicates the observed value, the bold black line indicates the median.


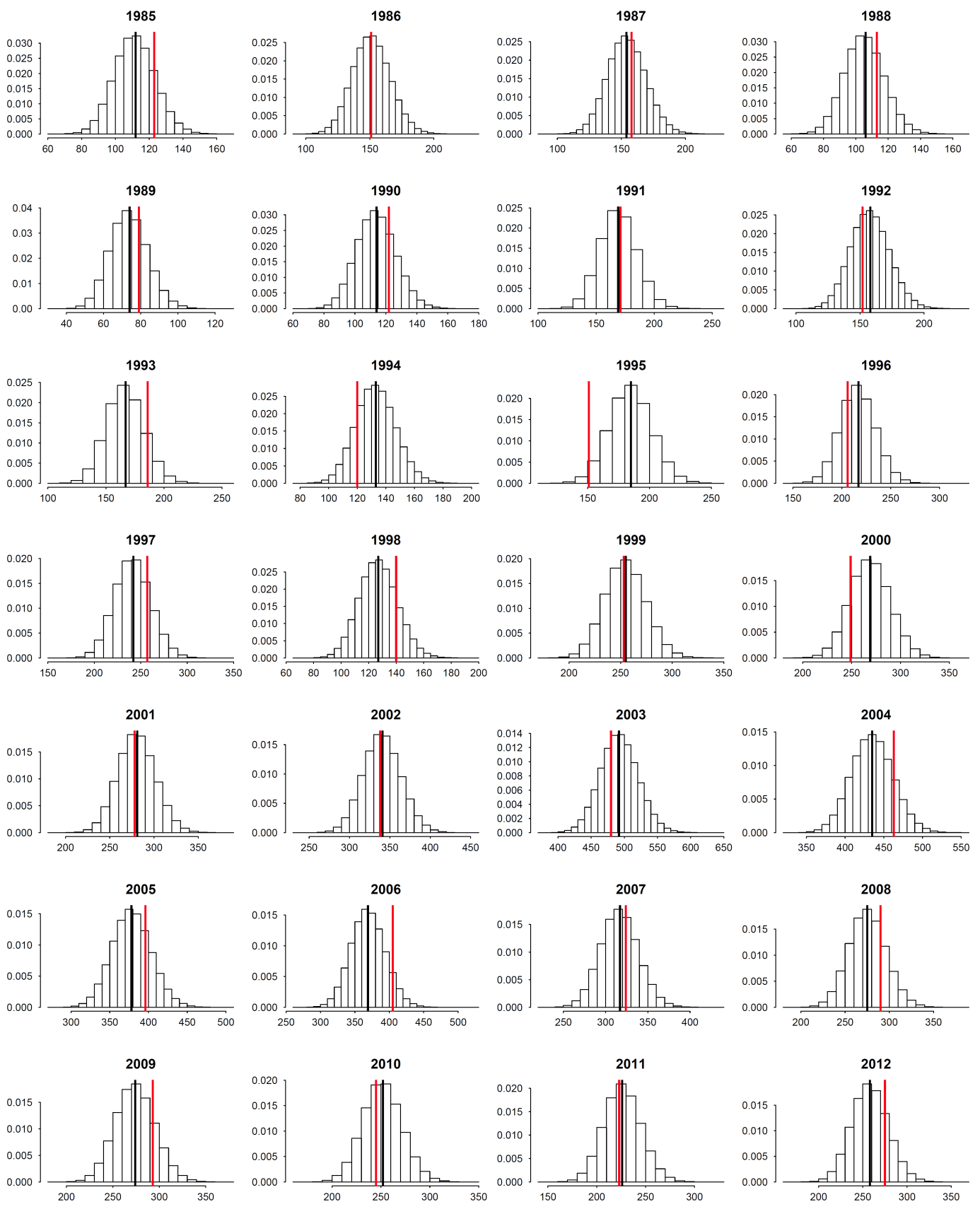


**Figure S17**. Posterior predictive distribution of the fledgling count from breeding pairs composed of two experienced breeders in each year across the study period. In abscissa of the histogram is the value, in ordinate is the density. The red line indicates the observed value, the bold black line indicates the median.


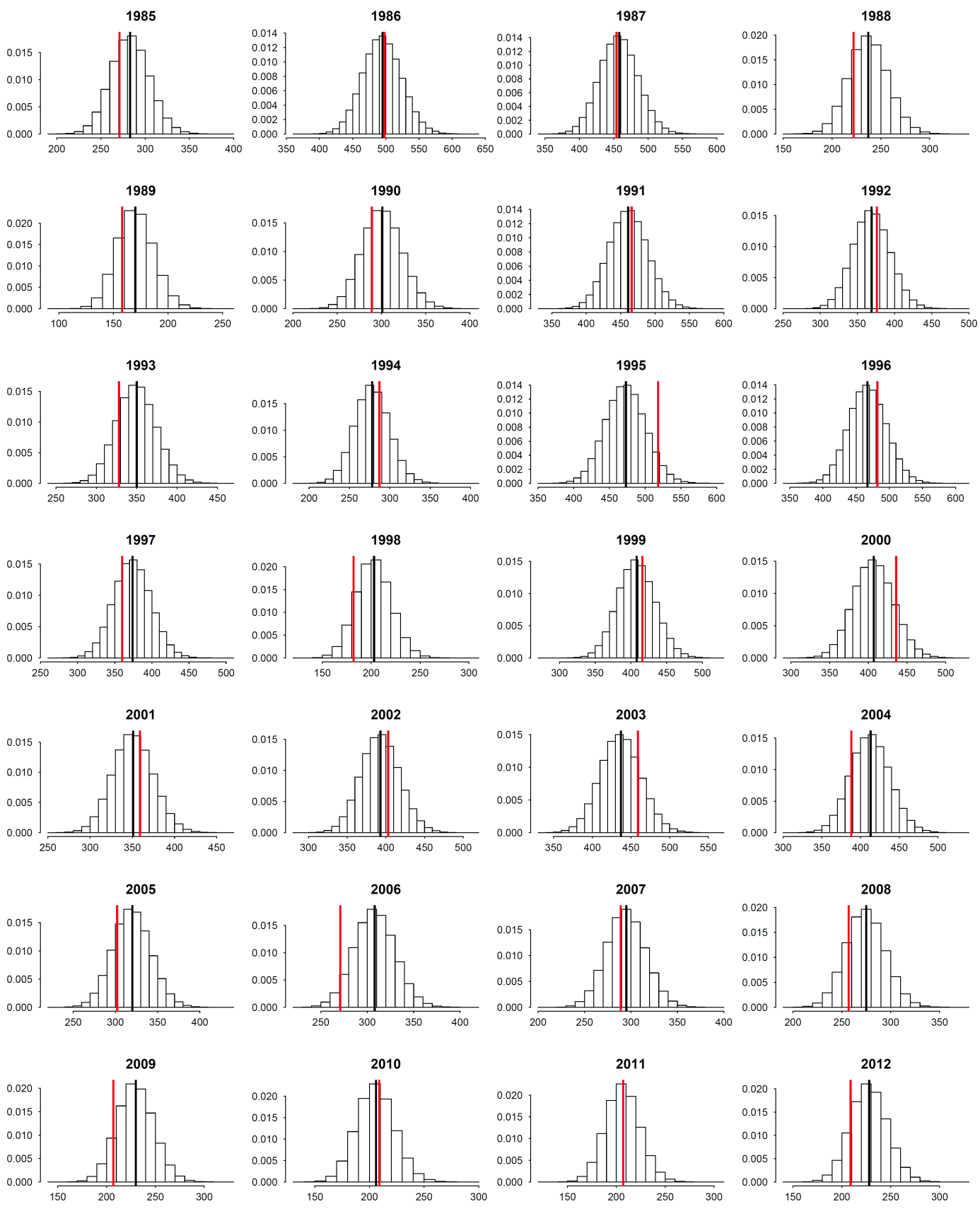


**Figure S18**. Posterior predictive distribution of the fledgling count from breeding pairs composed of either two breeders of different experience, or at least one breeder of unknown experience, in each year across the study period. In abscissa of the histogram is the value, in ordinate is the density. The red line indicates the observed value, the bold black line indicates the median.

### S4.3 References

Green, M. J., Medley, G. F., & Browne, W. J. (2009). Use of posterior predictive assessments to evaluate model fit in multilevel logistic regression. *Veterinary research, 40*(4), 1-10. <https://doi.org/10.1051/vetres/2009013>

Greenhill, B., Ward, M. D., & Sacks, A. (2011). The separation plot: A new visual method for evaluating the fit of binary models. *American Journal of Political Science, 55*(4), 991-1002. <https://doi.org/10.1111/j.1540-5907.2011.00525.x>

Gelman, A., Meng, X. L., & Stern, H. (1996). Posterior predictive assessment of model fitness via realized discrepancies. *Statistica sinica*, 733-760.

## APPENDIX S5

## Specification and results of the derived analyses

Several explanations provided in this appendix refer to parameters defined in Appendix S2 (details of the integrated population model).

**Contents:**

**S5.1 Growth rate of the breeding population (p. 54)**

**S5.2 Hypothetical population projections without the pulse of immigrants (p. 55)**

**S5.3 Calculation details and posterior distributions of derived parameters (p. 56)**

**S5.4 Partial correlation analyses (p. 59)**

**S5.5 Predictability in key demographic features (p. 64)**

**S5.6 References (p. 66)**

### S5.1 Growth rate of the breeding population

The annual breeding population growth rate in year *t* (*λ_t_*) is defined as follows:

$$\text{λ}_{\text{t}}\text{ = }\frac{\text{N}_{\text{F,t}}\text{ +}\text{ N}_{\text{E,t}}}{\text{N}_{\text{F,t-1}}\text{ +}\text{ N}_{\text{E,t-1}}}\text{ = }\frac{\text{N}_{\text{B,t}}}{\text{N}_{\text{B,t-1}}}$$

where $\text{N}_{\text{B,t}}$ is the number of breeders in year *t*.

If we consider a geometric growth of the population with a constant (average) growth rate ($\bar{\lambda}$) from the starting number of breeders in 1985, then we have:

$$\text{N}_{\text{B,t}}\text{ = }\text{N}_{\text{B,1985}}\text{ }{\bar{\text{λ}}}^{\text{t}}$$

with *t* ϵ {1986,...,2012}.

Using a log-transformation, we have:

${\text{log(}\text{N}}_{\text{B,t}}\text{)=}{\text{log(}\text{N}}_{\text{B,1985}}\text{)+}\text{t}\text{ log(}\bar{\text{λ}}\text{)}$ .

Thus, the slope of an ordinary least squares regression line of log($\text{N}_{\text{B},\text{t}}$) (*t* ϵ {1985,...,2012}) against *t* will be $\text{log(}\bar{\text{λ}}\text{)}$, and the intercept will be ${\text{log(}\text{N}}_{\text{B}\text{,1985}}\text{ )}$. The exponential slope of the regression line is thus $\bar{\text{λ}}$.

Accordingly, we performed an ordinary least squares regression of $\text{N}_{\text{B},\text{t}}$ against *t* in each posterior sample to get a posterior distribution of $\bar{\text{λ}}$. Results are given in the main text of the paper.

### S5.2 Hypothetical population projections without the pulse of immigrants

To evaluate the importance of immigration, we computed additional population projections without the pulse of immigrants were performed following the projection equation (see Appendix S2) but without adding the number of immigrants to the number of first-time breeders each year. The equation was thus written as follows:

$\left[ \begin{matrix} \text{N}_{\text{Y}} \\ \text{N}_{\text{P2}} \\ \text{N}_{\text{P3}} \\ \text{N}_{\text{P4}} \\ \text{N}_{\text{P5}} \\ \text{N}_{\text{P6}} \\ \text{N}_{\text{F}} \\ \text{N}_{\text{E}} \\ \text{N}_{\text{S}} \end{matrix} \right]_{\text{t}}\text{=}\text{ }\left[ \begin{matrix} 0 & 0 & 0 & 0 & 0 & 0 & \text{ϕ}_{0}\text{π}_{\text{f}} & \text{ϕ}_{0}\text{π}_{\text{e}} & 0 \\ \text{ϕ}_{0} & 0 & 0 & 0 & 0 & 0 & 0 & 0 & 0 \\ 0 & \text{ϕ}_{\text{2}}\text{(1-}\text{ρ}_{\text{3}}\text{)} & 0 & 0 & 0 & 0 & 0 & 0 & 0 \\ 0 & 0 & \text{ϕ}_{\text{2}}\text{(1-}\text{ρ}_{\text{4}}\text{)} & 0 & 0 & 0 & 0 & 0 & 0 \\ 0 & 0 & 0 & \text{ϕ}_{\text{2}}\text{(1-}\text{ρ}_{\text{5}}\text{)} & 0 & 0 & 0 & 0 & 0 \\ 0 & 0 & 0 & 0 & \text{ϕ}_{\text{2}}\text{(1-}\text{ρ}_{\text{6}}\text{)} & 0 & 0 & 0 & 0 \\ 0 & \text{ϕ}_{\text{2}}\text{ρ}_{\text{3}} & \text{ϕ}_{\text{2}}\text{ρ}_{\text{4}} & \text{ϕ}_{\text{2}}\text{ρ}_{\text{5}} & \text{ϕ}_{\text{2}}\text{ρ}_{\text{6}} & \text{ϕ}_{\text{2}} & 0 & 0 & 0 \\ 0 & 0 & 0 & 0 & 0 & 0 & \text{ϕ}_{\text{2}}\text{ψ}_{\text{b}} & \text{ϕ}_{\text{2}}\text{ψ}_{\text{b}} & \text{ϕ}_{\text{2}}\text{ψ}_{\text{s}} \\ 0 & 0 & 0 & 0 & 0 & 0 & \text{ϕ}_{\text{2}}\text{(1-}\text{ψ}_{\text{b}}\text{)} & \text{ϕ}_{\text{2}}\text{(1-}\text{ψ}_{\text{b}}\text{)} & \text{ϕ}_{\text{2}}\text{(1-}\text{ψ}_{\text{s}}\text{)} \end{matrix} \right]_{\text{t-1}}\left[ \begin{matrix} \text{N}_{\text{Y}} \\ \text{N}_{\text{P2}} \\ \text{N}_{\text{P3}} \\ \text{N}_{\text{P4}} \\ \text{N}_{\text{P5}} \\ \text{N}_{\text{P6}} \\ \text{N}_{\text{F}} \\ \text{N}_{\text{E}} \\ \text{N}_{\text{S}} \end{matrix} \right]_{\text{t-1}}$.

For the population projection, we started from the initial vector of state-specific population sizes and used year-specific demographic parameters estimated with each posterior sample. We followed the same method to calculate the average breeding population growth rate across 1985-2012. Results are given in the main text of the paper.

The projections suggest that the average breeding population growth rate would have been 0.86 [0.84,0.88] without immigration. Therefore, the population would have declined from 2078 [2050,2104] in 1985 to 37 [18,56] breeding pairs in 2012.

### S5.3 Calculation details and posterior distributions of derived parameters

The formula used to calculate the annual growth rate between year *t*-1 and year *t* (also given at the beginning of this appendix) was:

$$\text{λ}_{\text{t}}\text{ = }\frac{\text{N}_{\text{F,t}}\text{ +}\text{ N}_{\text{E,t}}}{\text{N}_{\text{F,t-1}}\text{ +}\text{ N}_{\text{E,t-1}}} .$$

We defined the integrative recruitment rate as the proportion of first-time breeders among individuals that have never bred before within all age classes that could recruit individuals in the current year *t* (age 3, ..., 6). In other words, it is the number of local first-time breeders in the current year *t* (i.e. first-time breeders minus immigrants) divided by the number of prebreeders of age 3, ..., 6 plus the number of local first-time breeders. The formula used to calculate the integrative recruitment rate $\text{ρ}_{\text{all}}$ was thus:

$$\text{ρ}_{\text{all}}\text{ =} \frac{\text{N}_{\text{F,t}}\text{ -}\text{ N}_{\text{I,t}}}{\text{N}_{\text{P3,t}}\text{ +}\text{ N}_{\text{P4,t}}\text{ +}\text{ N}_{\text{P5,t}}\text{ +}\text{ N}_{\text{P6,t}}\text{ +} \text{N}_{\text{F,t}}\text{ -}\text{ N}_{\text{I,t}}} .$$

We calculated the annual proportion of immigrants (*ω_t_*) as the ratio of the number of immigrants and the number of breeders in the current year *t*:

$$\text{ω}_{\text{t}}\text{ = }\frac{\text{ N}_{\text{I,t}}}{\text{N}_{\text{F,t}}\text{ +}\text{ N}_{\text{E,t}}} .$$

We defined the number of present nonbreeders as the number of prebreeders (age 2, ..., 6) plus the number of skippers present in the area. Thus, we added the number of skippers to the number of prebreeders multiplied by the resighting rate of prebreeders in the current year. We did not correct the number of skippers by their resighting rate because it was virtually equal to 1. The formula used to calculate the number of nonbreeders $\text{N}_{\text{PN,t}}$ was thus:

$$\text{N}_{\text{PN,t}}\text{= }{\text{p}_{\text{p}}\text{(}\text{N}}_{\text{P3,t}}\text{ +}\text{ N}_{\text{P4,t}}\text{ +}\text{ N}_{\text{P5,t}}\text{ +}\text{ N}_{\text{P6,t}}\text{) + }\text{N}_{\text{PN,t}} .$$

We defined the population breeding success *П_t_* as the *per nest* (i.e. twice *per capita*) average of the breeding success of first-time breeders and experienced breeders weighted by their respective proportion in the population:

$$\text{П}_{\text{t}}\text{=2}\left( {\text{ }\text{π}}_{\text{f,t}}\frac{\text{N}_{\text{F,t}}}{\text{N}_{\text{F,t}}\text{+}\text{N}_{\text{E,t}}}\text{+}\text{π}_{\text{e,t}}\frac{\text{N}_{\text{E,t}}}{\text{N}_{\text{F,t}}\text{+}\text{N}_{\text{E,t}}} \right) .$$

Graphical summaries of the year-specific growth rates, integrative recruitment rates, the immigration rates, and the number of nonbreeders present (Fig S19, S20, S21, S22, respectively) are provided below.


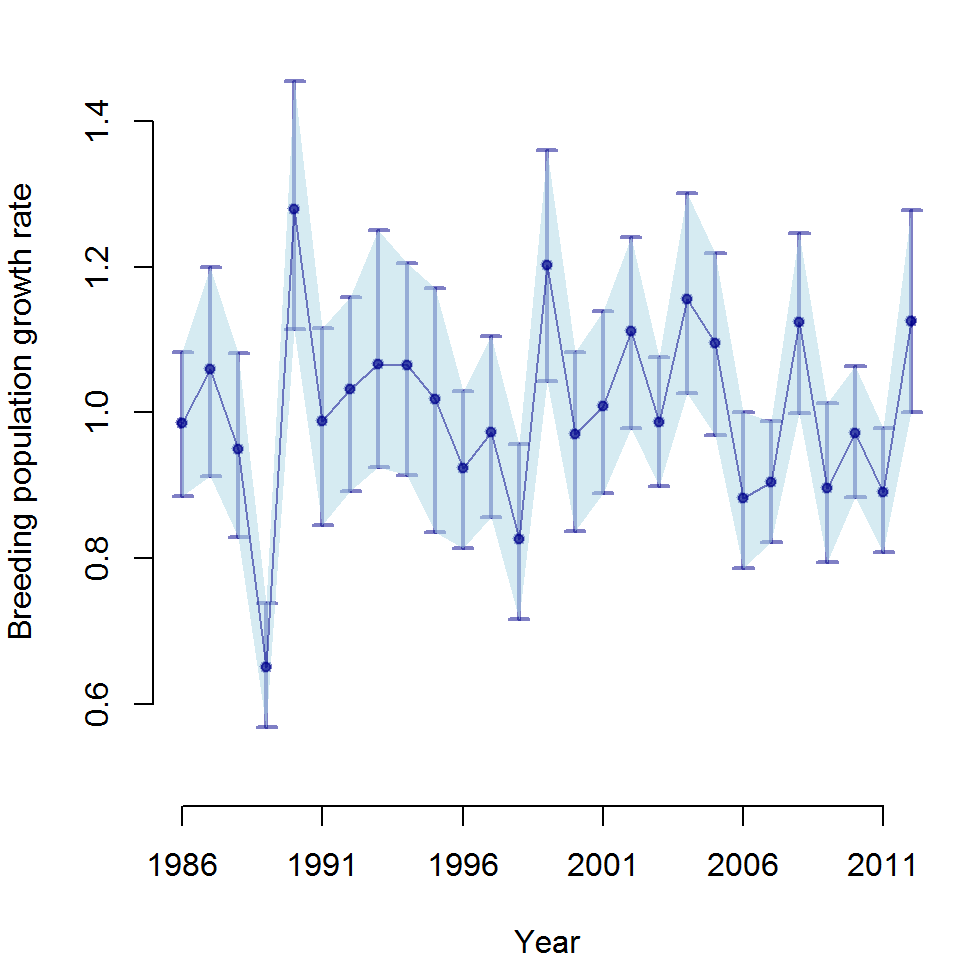


**Figure S19**. Estimates of kittiwake annual breeding population growth rates across 1986−2012 in the Cap Sizun population. Color backgrounds and segments indicate 95%CI.


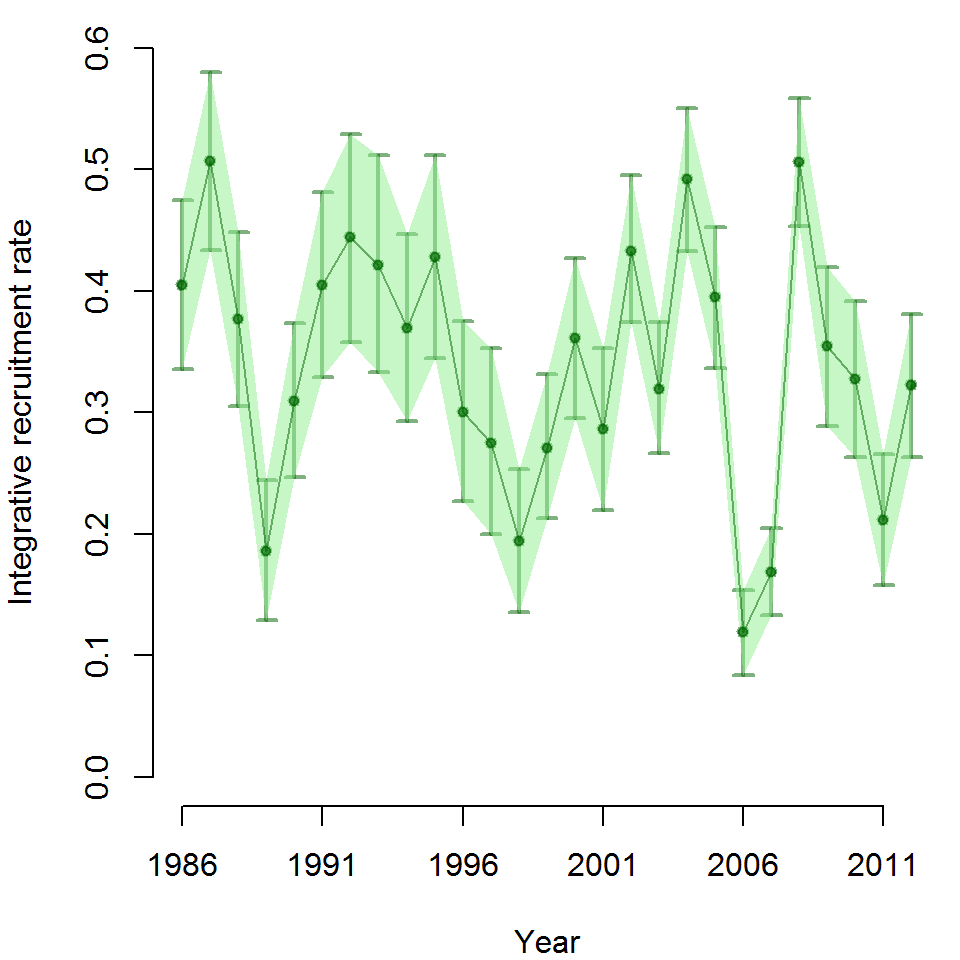


**Figure S20**. Estimates of kittiwake integrative recruitment rates across 1986−2012 in the Cap Sizun population. Color backgrounds and segments indicate 95%CI.


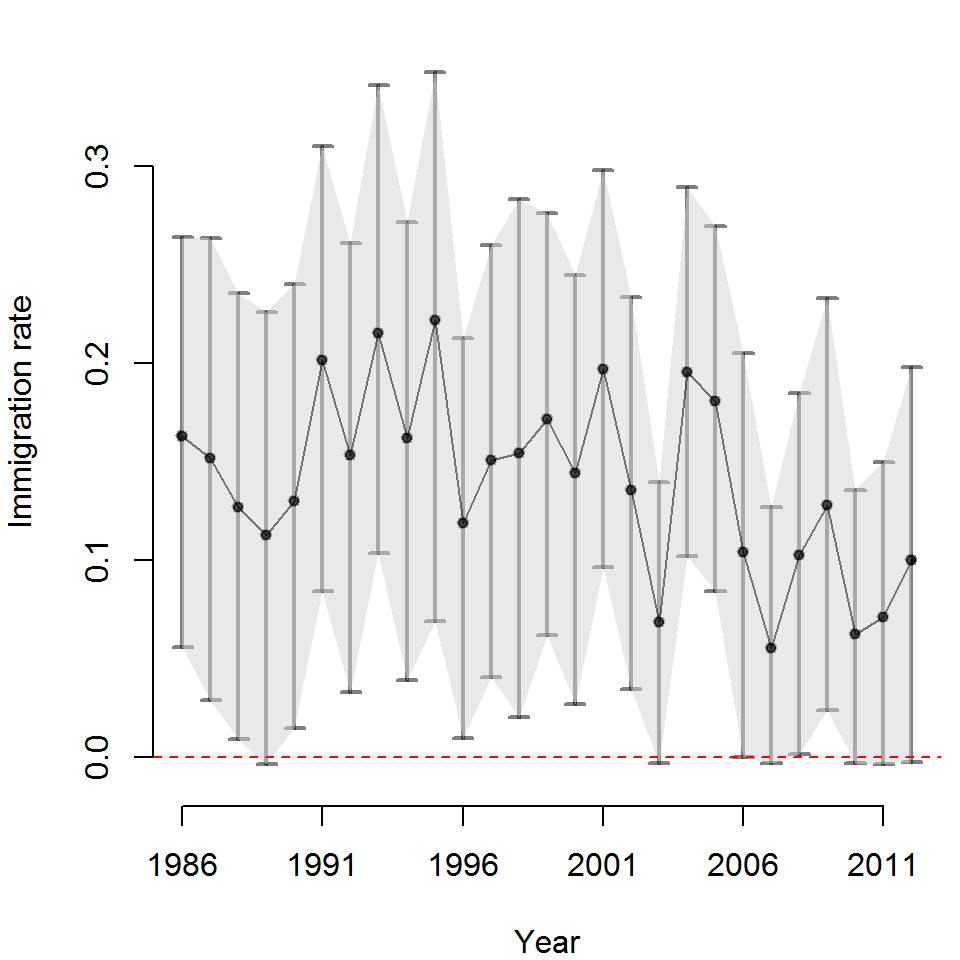


**Figure S21**. Estimates of kittiwake immigration rates across 1986−2012 in the Cap Sizun population. Grey backgrounds and segments indicate 95%CI.


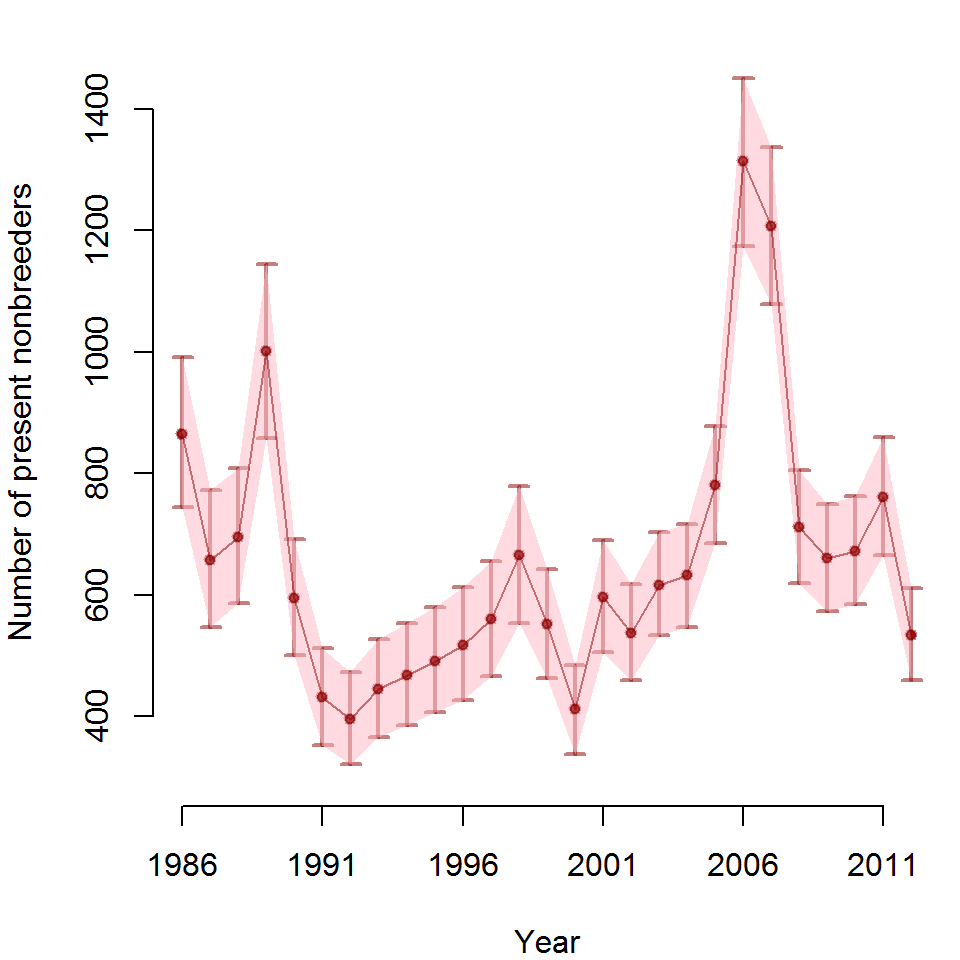


**Figure S22**. Estimates of kittiwake numbers of present nonbreeders across 1986−2012 in the Cap Sizun population. Color backgrounds and segments indicate 95%CI.

### S5.4 Partial correlation analyses

***S5.4.1 Additional details on the method***

Partial correlation measures the correlation between two random variables, with the effect of a set of control random variables removed. More precisely, a partial correlation between variables X and Y while controlling for *n* variables Z_1_, ..., Z_n_ is the correlation between the residuals of two linear regressions: (i) a regression with X as the response and Z_1_, ..., Z_n_ as the explanatory variables, and (ii) a regression with Y as the response and Z_1_, ..., Z_n_ as the explanatory variables.

Accordingly, to calculate each partial correlation between two focal variables from the model, we computed (in each posterior sample): (i) one ordinary least squares (OLS) regression between the first focal variable and a set of control variable, (ii) one OLS regression between the second focal variable and the same set of control variable. Then we calculated the correlation between the residuals of the two latter regressions. We also computed an OLS regression between the residuals of the two latter regressions to add the partial regression line on the partial residual plot (see e.g. Fig. 4 of main text).

***S5.4.1 Additional details on the results***

Hereafter we provide partial residual plots for partial correlation which had a 95%CRI that included zero (that were not provided in the main text). Fig. S23 is for partial correlations in the purpose of examining contributions to the breeding population growth rate, and Fig. S24 is for partial correlations in the purpose of examining individual breeding motivations. We also provide partial regression plots for partial correlation between the number of immigrants instead of the immigration rate) in year *t* and either population breeding success at *t-1*, the number of breeders at *t-1*, or the number of nonbreeders present at *t-1* (Fig. S25).

Note here that we checked whether the positive partial correlation between breeding rate of former breeders and population breeding success (Fig. 4f) was driven by the single point with especially low breeding rate and population breeding success compared to all other points (i.e. the point down to the left on Fig. 4f). We thus computed the partial correlation excluding this point. This estimate was still providing conclusive evidence of a positive relationship (mean and 95%CRI: 0.21 [-0.01,0.42], probability that the coefficient is positive: 0.97). This demonstrated the robustness of our conclusion regarding occurrence of a positive relationship between breeding rate of former breeders and population breeding success (while controlling for the number of breeders and the number of present nonbreeders).

Finally, we also derived the posterior distribution of the correlations between number of competitors in year *t* and population breeding success in year *t+1* (Table S2).


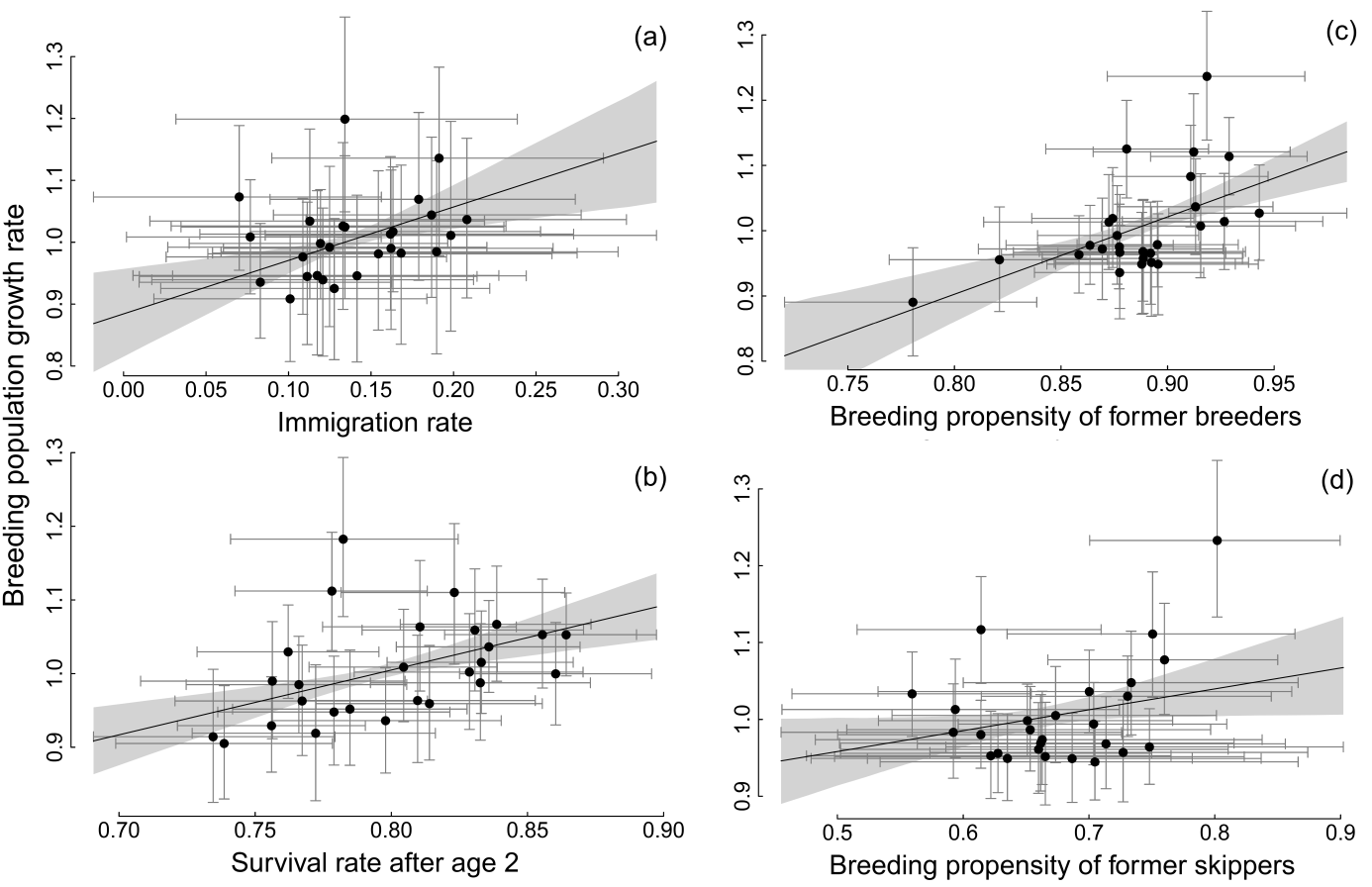


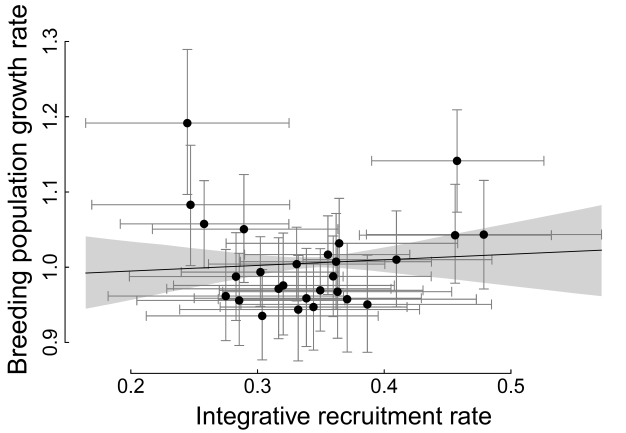


**Figure S23**. Partial residual plot for the partial correlation between the growth rate and integrative recruitment rate (while controlling for the effect of adult survival, population breeding success, and breeding rate of former breeders and skippers). The partial correlation was 0.08 [-0.20,0.36] (posterior mean [95%CI]). Residuals were centered around the variable mean to rescale variation within the original range. Points indicate posterior means and segments indicate 95% credible intervals. The solid line is the posterior mean of the corresponding OLS regression line, along with the 95% credible interval in grey background.


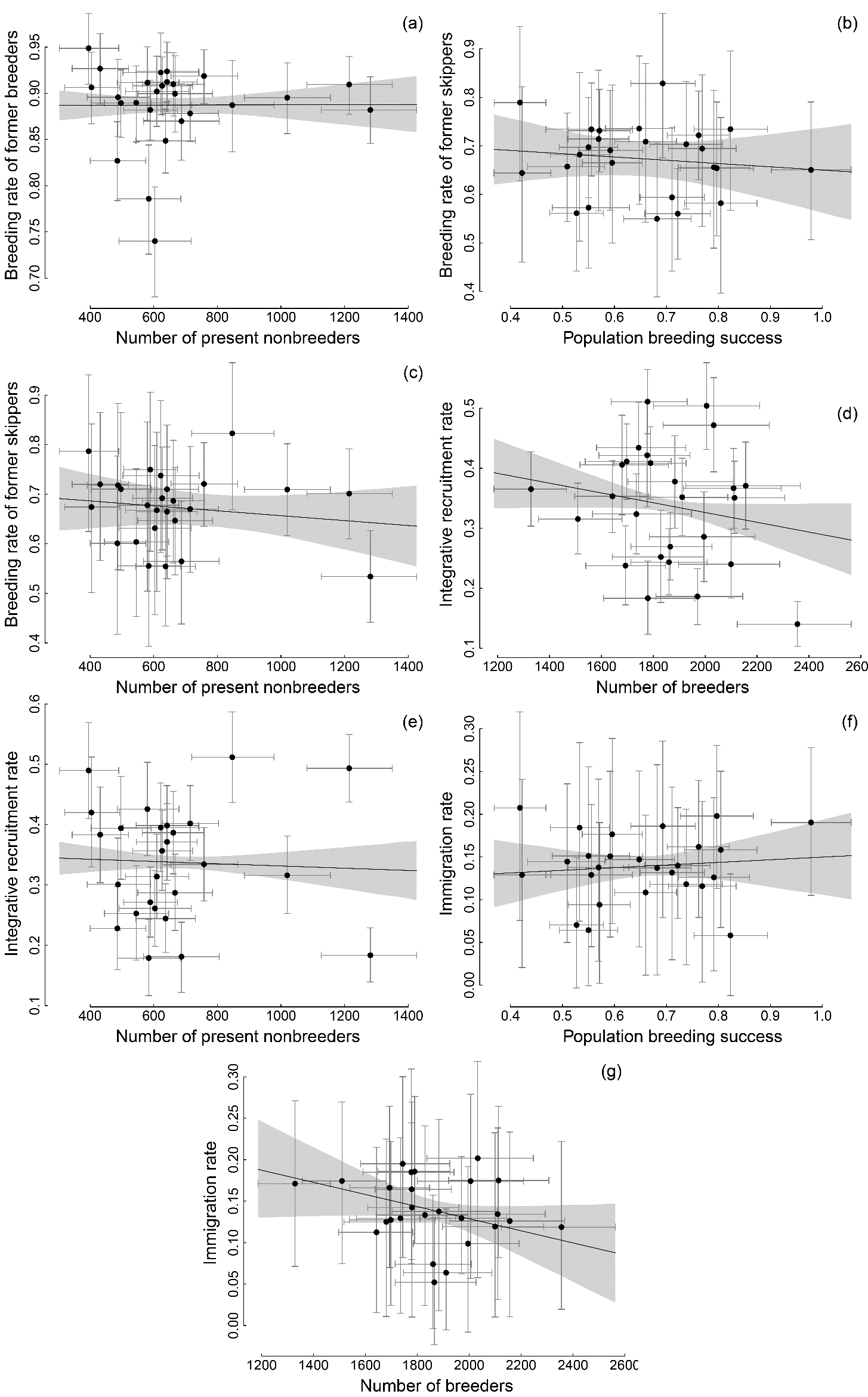


**Figure S24**. Graphical summary of the associations between breeding propensity in year *t* (y-axis) and population breeding success (mean number of fledglings per nest) or conspecific competitor abundances (number of breeder or present nonbreeders) in year *t-1* (x-axis). Relationships presented here are those with weak or no evidence for a positive or negative partial correlation. These are partial residual plots representing partial correlations of a response variable (y-axis) against one covariate (x-axis), while controlling for the remaining covariates; residuals were centred on the variable mean to rescale variation within the original range. Points: posterior means of rescaled residuals; segments: 95%CRIs. Solid line: posterior mean of regression line; grey background: 95%CRI. Numerical summaries are in Table 1.


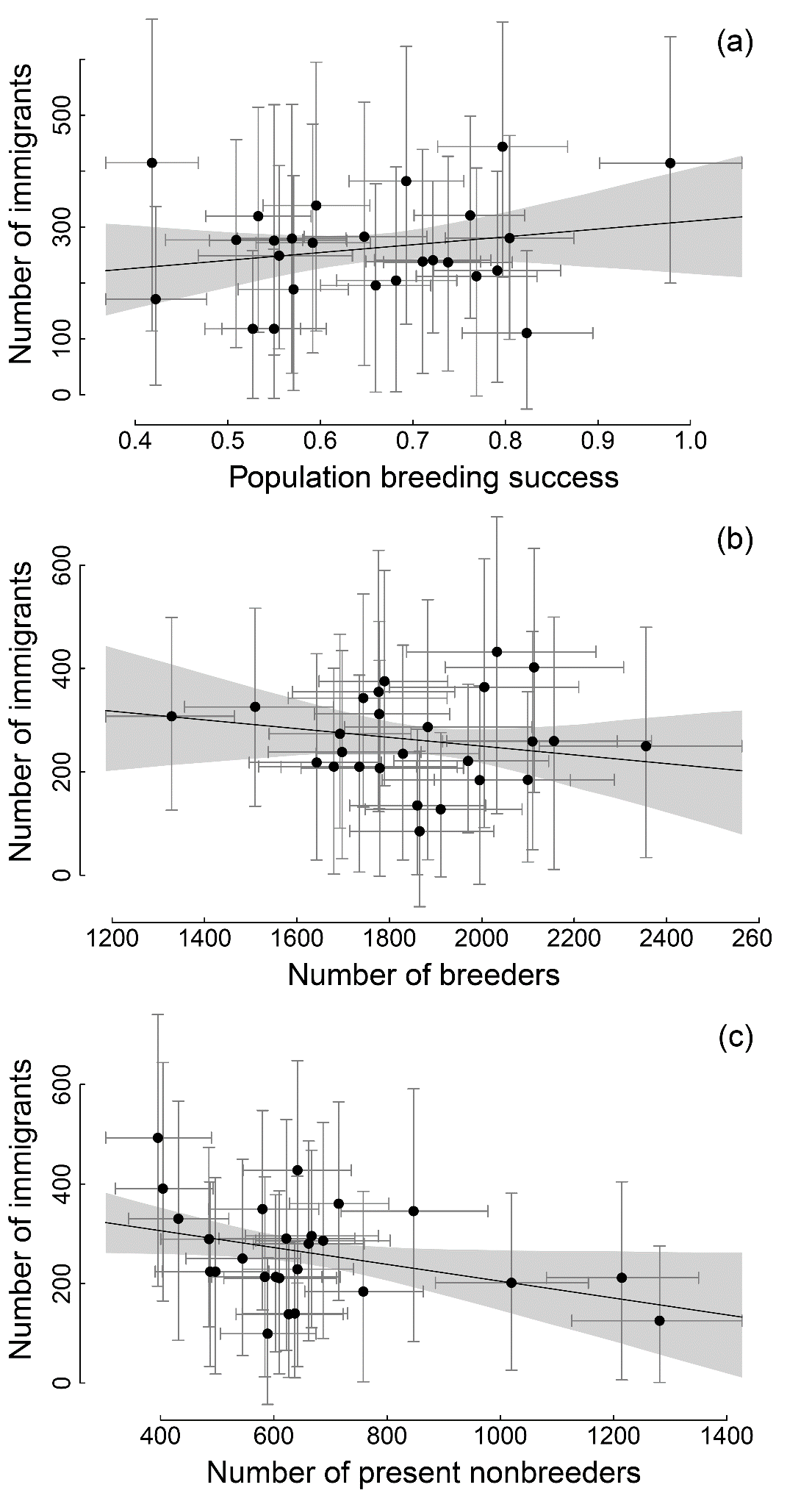


**Figure S25**. Partial residual plots of the relationships between the number of immigrants in year *t* and (a) population breeding success at *t-1*, (b) number of breeders at *t-1*, (c) number of nonbreeders present at *t-1.* The partial correlation was (a) 0.15 [-0.13,0.41], (b) -0.14 [-0.41, 0.14], and (c) -0.28 [-0.53,0.01]. See further details in *Materials and Methods*, and partial correlation values in *Results*. Specifications are the same as for Fig. S23.

**Table S2**. Pearson’s correlation coefficients between the number of competitors in the population and breeding success.

| Number of competitors considered | | Correlation *ρ* with population breeding success at *t+1* | | |
| --- | --- | --- | --- | --- |
|  |  | Mean | 95%CI | Pr(*ρ*>0) |
| In year *t* | Breeders | -0.12 | [-0.30,0.05] | 0.07 |
|  | Present nonbreeders | -0.11 | [-0.22,0.00] | 0.03 |
| In year *t+1* | Breeders | 0.13 | [-0.15,0.30] | 0.88 |
|  | Present nonbreeders | -0.17 | [-0.29,-0.05] | 0.00 |

### S5.5 Predictability in key demographic features

***S5.5.1 Temporal autocorrelation in demographic features***

To evaluate predictability in demographic features, we calculated the temporal autocorrelation in key demographic features: population breeding success, and number of breeders and number of nonbreeders present. Population breeding success was calculated as the *per nest* average breeding success of inexperienced and experienced breeders weighted by their respective proportion among breeders (see Appendix S2 for calculation details and Appendix S3 for a graphical summary of this population breeding success across years). More precisely, we calculated the sample autocorrelation function (the correlation of a random variable with itself at different points in time; Cryer & Chan, 2008, Schumway & Stoffer, 2011) at lag 1 year in each posterior sample. The formula of the sample autocorrelation function at lag *k* (*r*(*k*)) is the following (results are given in the article):

$$r(k) = \frac{\sum_{t} (x_{t}-\bar{x})(x_{t+k}-\bar{x})}{\sum_{t} {(x_{t}-\bar{x})}^{2}}$$

where $\text{x}_{\text{t}}$ is the value observed in the time series of a random variable in year *t*, and $\bar{\text{x}}$ is the average across the time series.

The corresponding results suggested that the population breeding success, number of breeders, and number of prebreeders plus skippers present, were predictable from one year to the next (respectively, autocorrelation coefficients at lag one year: 0.43 [0.34, 0.52], 0.40 [0.20, 0.56] and 0.56 [0.47, 0.65]).

However, first, autocorrelation estimates are known to be biased down for short times series (under approximately 50 times steps; Box & Jenkins, 1976, Huitema & Mckean, 1991). Because they were calculated on time-series lasting only 27 years, the autocorrelation values we reported are very likely to lower than the true autocorrelation. Second, these autocorrelation values should be compared to the confidence interval of the sample autocorrelation function (here at lag 1) for non-autoregressive processes. The usual reference for non-autoregressive processes is a simple white noise or moving average, for which the autocorrelation function at lag 1 in a large series is approximately normally distributed with mean 0 an standard deviation *s_r_*_(1)_:

*s_r_*_(1)_ = $\frac{1}{\sqrt{n}}$

where *n* is the number of observations in the time series.

For a series of 27 observations, the 95% credible interval would thus be approximately 2/$\sqrt{\text{27}}$ = 0.38, which falls below the reported mean posterior values for autocorrelations at lag 1 (see above), but above the lower limit of the 95%CRIs for population breeding success and number of breeders. This 95%CRI for a non-autoregressive is valid for large time series, but the reported autocorrelation values are biased down in small series as ours. Thus, our results suggest that there is some autocorrelation, but our time series are too short for a proper assessment.

***S5.5.2 Correlation among demographic features from one year to the next***

The number of breeders in year *t* was predictive of the number of former breeders among breeders in year *t+1*: the mean correlation derived from posterior samples was 0.75, and 95%CRI was [0.65,0.84]. The number of nonbreeders in year *t* was predictive of the number of local first-time breeders in year *t+1*: correlation was 0.74 [0.65,0.82]. The number of nonbreeders in year *t* was predictive of the number of former skippers among breeders in year *t+1*: correlation was 0.49 [0.38,0.60].

### S5.6 References

Box, G. E., Jenkins, G. M., Reinsel, G. C., & Ljung, G. M. (2015). *Time series analysis: forecasting and control.* John Wiley & Sons, New York, USA*.*.

Cryer, J. D., & Chan, K. S. (2008*). Time series analysis: with applications in R.* Springer*,* New York, USA*.*

Huitema, B. E., & McKean, J. W. (1991). Autocorrelation estimation and inference with small samples. *Psychological Bulletin, 110*(2), 291. <https://doi.org/10.1037/0033-2909.110.2.291>

Shumway, R. H., & Stoffer, D. S. (2011). *Time series analysis and its applications with R examples* (3^rd^ ed.). Springer, New York, USA.
